# Response of reaction mechanisms to electric-field catalysis on carbon nanotubes in microfluidic reactors†

**DOI:** 10.1039/d5sc02934a

**Published:** 2025-05-28

**Authors:** M. Ángeles Gutiérrez López, Alenka Marsalek, Naomi Sakai, Stefan Matile

**Affiliations:** a Department of Organic Chemistry, University of Geneva Geneva Switzerland stefan.matile@unige.ch www.unige.ch/sciences/chiorg/matile/ +41 22 379 6523; b National Centre of Competence in Research (NCCR), Molecular Systems Engineering BPR 1095 Basel Switzerland

## Abstract

If accessible under scalable bulk conditions, remote control of charge translocation during a molecular transformation with oriented external electric fields promises to make a major contribution to sustainable organic synthesis. Here, we show that the combination of electric-field catalysis with anion–π and cation–π catalysis on carbon nanotubes in electromicrofluidic devices can influence reaction mechanisms under scalable bulk conditions. At high voltage, epoxide-opening ether cyclizations that do not occur without electric fields proceed to completion. Sensitivity to the orientation of the applied field indicates the nature of the rate-limiting motif in the transition state. Increasing magnitude of the electric field can change reaction mechanisms and accelerate the intrinsically disfavored pathways. Substrate positioning on the polarized nanotube surfaces enhances electric-field control over reaction mechanism. These results support the promise of electric-field anion–π and cation–π catalysis on carbon nanotubes in electromicrofluidic devices for use in organic synthesis.

## Abstract 2

Scalable electric-field catalysis on carbon nanotubes in electromicrofluidic reactors is shown to accelerate and direct reactions toward intrinsically disfavored products.

## Graphical abstract

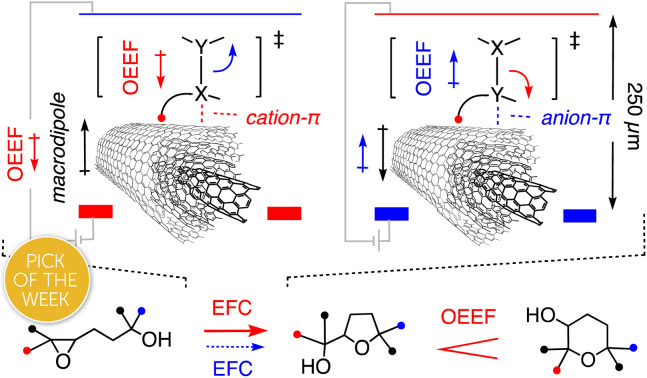

The perspective of accelerating and directing the movement of electrons during a reaction with oriented external electric fields (OEEFs) has attracted much attention because it promises to impact organic synthesis fundamentally (Fig. 1a and b).^1–19^ Both theory and experimental evidence in biology support these great expectations from electric-field catalysis (EFC).^1–19^ Recently, we realized that most of the many practical problems that have prevented systematic development of EFC under scalable bulk conditions so far could possibly be addressed by using multiwalled carbon nanotubes (MWCNTs)^20–25^ in electromicrofluidic devices (Fig. 1a and b).^26^ Drop-casted on the graphite electrode surface, the polarization of MWCNTs by the applied OEEF induces strong macrodipoles^27^ for strong cation–π^28,29^ and anion–π interactions,^25,27,30–34^ respectively, depending on the orientation of the field. These cation–π and anion–π interactions then support the applied OEEF to accelerate and direct the movement of electrons during the reaction of interest. Moreover, the formation of Gouy–Chapman–Stern electrical double layers (EDL)^35^ already in polar solvents will shorten the effective distance between formal electrodes from the 250 μm set by the reactor's dimensions to a few nm between one electrical layer and the oppositely charged electrode. This will produce effective local OEEFs that are at least three orders of magnitude higher than the apparent applied OEEF.^3^

**Fig. 1:**
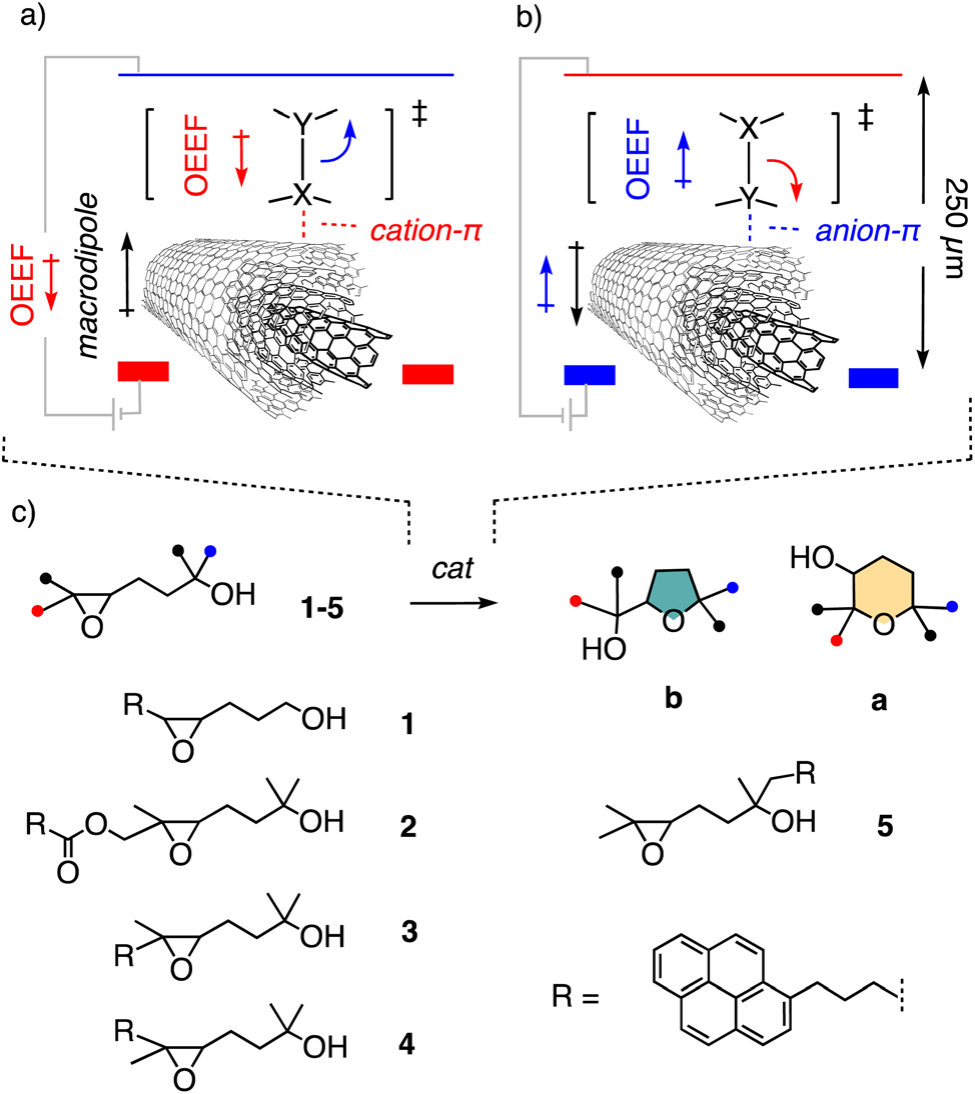
(a) Electric-field cation–π and (b) anion–π catalysis to cleave the bond *X*–*Y* on MWCNTs in electromicrofluidic devices (OEEF = oriented external electric field). (c) Structure of previous (1) and new substrates (2–5) and products a (anti-Baldwin) and b (Baldwin) of epoxide-opening ether cyclization.

This envisioned use of electromicrofluidic reactors to elaborate on (an/cat)ion–π EFC fundamentally differs from the redox chemistry the reactors were made for.^36–40^ Control experiments confirmed that oxidation of hydroquinone (*E*_ox_ = 400 mV *vs.* SCE) and (auto)oxidative aromatization of terpinines are negligible within voltages up to *V* = ±5.0 V.^41^ Analogous to the relation of ion–π and electron transfer processes, ion–π EFC is expected to occur below the threshold of electron transfer and follow the principles of supramolecular chemistry rather than redox chemistry.

In the selected, commercially available reactor, the electrodes (5 × 5 cm^2^) are separated by a 0.25 mm fluorinated ethylene propylene foil with the flow channel, which results in a reactor volume of 0.3 mL and an exposed electrode surface area of 12 cm^2^.^26^ With continuous flow applied, a parabolic flow profile is expected, with radial diffusion in microchannels assuring uniform velocity,^42^ and decreasing flow rates thus primarily serve to increase reaction times.^26,41^ Drop-casted MWCNTs increase surface area and conductivity,^43–45^ and contribute to high effective local OEEFs as described above (Fig. 1).

The existence and relevance of anion–π EFC on MWCNTs in electromicrofluidic reactors have been explored with epoxide-opening ether cyclization, a reaction of importance in chemistry and biology^46–49^ (Fig. 1c).^26^ The cyclization of substrates like 1 can afford either the *exo*-product 1b, favored according to the Eschenmoser–Dunitz–Baldwin guidelines,^50–53^ or the ring-expanded “anti-Baldwin” *endo*-product 1a. Substrate 1 is equipped with a pyrene interfacer, which has been essential to increase contact time on the MWCNTs, *i.e.*, stabilize formal catalyst–substrate complexes.^26^ Without voltage applied, cyclization of 1 essentially did not occur during one passage through the electromicrofluidic reactor (Fig. 1c).^26^ With applied voltage, exclusive formation of the intrinsically favored Baldwin product 1b was observed. In the following, we use the same epoxide-opening ether cyclization to explore the possibility of identifying and manipulating reaction mechanisms with EFC, particularly to access intrinsically disfavored products.

Substrates 2–5 were prepared by target-oriented synthesis in up to 13 steps (Schemes 1, S1–S3†). For example, racemic *trans* epoxide 3 was synthesized from 1,4-butanediol 6 and pyrenebutyric acid 7 through synthetic intermediates 8–19. The key Wittig reaction between 10 and 11 gave 12 as a mixture of (*E*)/(*Z*) isomers in ∼1 : 1 ratio. The isomers were separated by preparative chiral HPLC on the level of intermediate 17, three steps from the end, and the (*Z*) isomer of 17 was used to prepare *cis* epoxide 4. The *trans* configuration of substrate 3 and *cis* configuration of substrate 4 were confirmed by NOESY NMR spectroscopy. The pnictogen-bonding catalyst 20 ^54–56^ was confirmed as a catalyst of choice in practice to violate the Baldwin rules most efficiently and convert substrate 3 to the anti-Baldwin product 3a in 80% yield, together with 14% of the otherwise favored 3b. The NMR and HPLC signatures of Baldwin and anti-Baldwin products of all substrates 2–5 were recorded and used to elaborate on EFC.

**Scheme 1:**
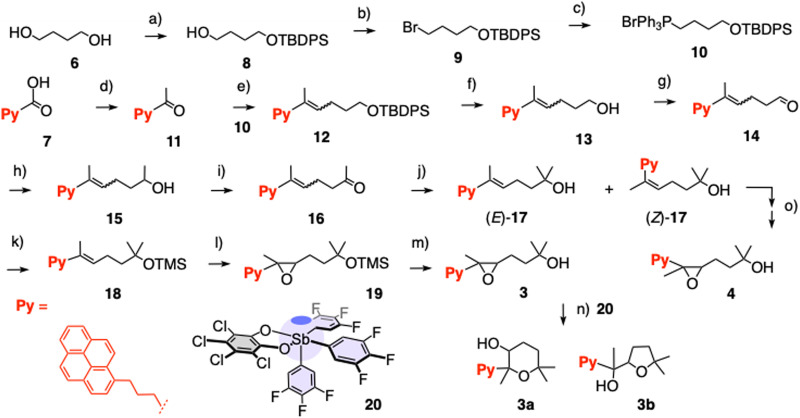
Synthesis of substrate 3 and products 3a and 3b. (a) 1. NaH, THF, 0 °C, 30 min; 2. TBDPSCl, THF, 0 °C, 2 h, quant. (b) PPh_3_, CBr_4_, CH_2_Cl_2_, 0 °C to RT, 2 h, 56%. (c) PPh_3_, toluene, 150 °C, 15 h, 56%. (d) MeLi, THF, −78 °C to RT, 3 h, 70%; (e) 1. 10, LiHDMS, THF, −78 °C to 0 °C, 30 min; 2. 11, −78 °C to RT, 15 h, 40%. (f) TBAF, THF, 0 °C to RT, 2 h, 91%; (g) DMP, CH_2_Cl_2_, 0 °C to RT, 3 h, 61%. (h) MeMgBr, dry Et_2_O, 0 °C to RT, 1 h, quant. (i) DMP, CH_2_Cl_2_, 0 °C to RT, 3 h, 78%. (j) MeMgBr, dry Et_2_O, 0 °C to RT, 1 h, 83% (*E* + *Z*). (k) DMAP, Et_3_N, TMSCl, CH_2_Cl_2_, RT, 1 h, 82%. (l) *m*-CPBA, CH_2_Cl_2_, 0 °C to RT, 1 h, 83%. (m) TBAF, THF, 0 °C to RT, 2 h, 94%. (n) 20, CH_2_Cl_2_, RT, 30 min, 95% (81% 3a, 14% 3b). (o) See ESI.†

EFC of epoxide-opening ether cyclizations is conceivable in combination with anion–π and cation–π catalysis following either concerted S_N_2- or stepwise S_N_1-type mechanisms (Fig. 2a). Activation of nucleophiles and leaving groups with (partial) alcoholate–π interactions and electric fields in transition state TS-1 account for an S_N_2-type mechanism with negative fields. However, while deprotonation of the alcohol boosts nucleophilicity, anion–π interactions of the resulting alcoholate with the MWCNT should weaken reactivity, exceeding nonetheless that of the original alcohol. Substrates with weakened nucleophiles, activated epoxides or both will open the epoxide before the nucleophile reacts. This will cause a shift from the concerted S_N_2-type mechanism in TS-1 to a stepwise S_N_1-type mechanism in TS-2, leading to the reactive intermediate RI-1. The same shift of mechanism could possibly be expected from stronger anion–π interactions and electric fields.

**Fig. 2:**
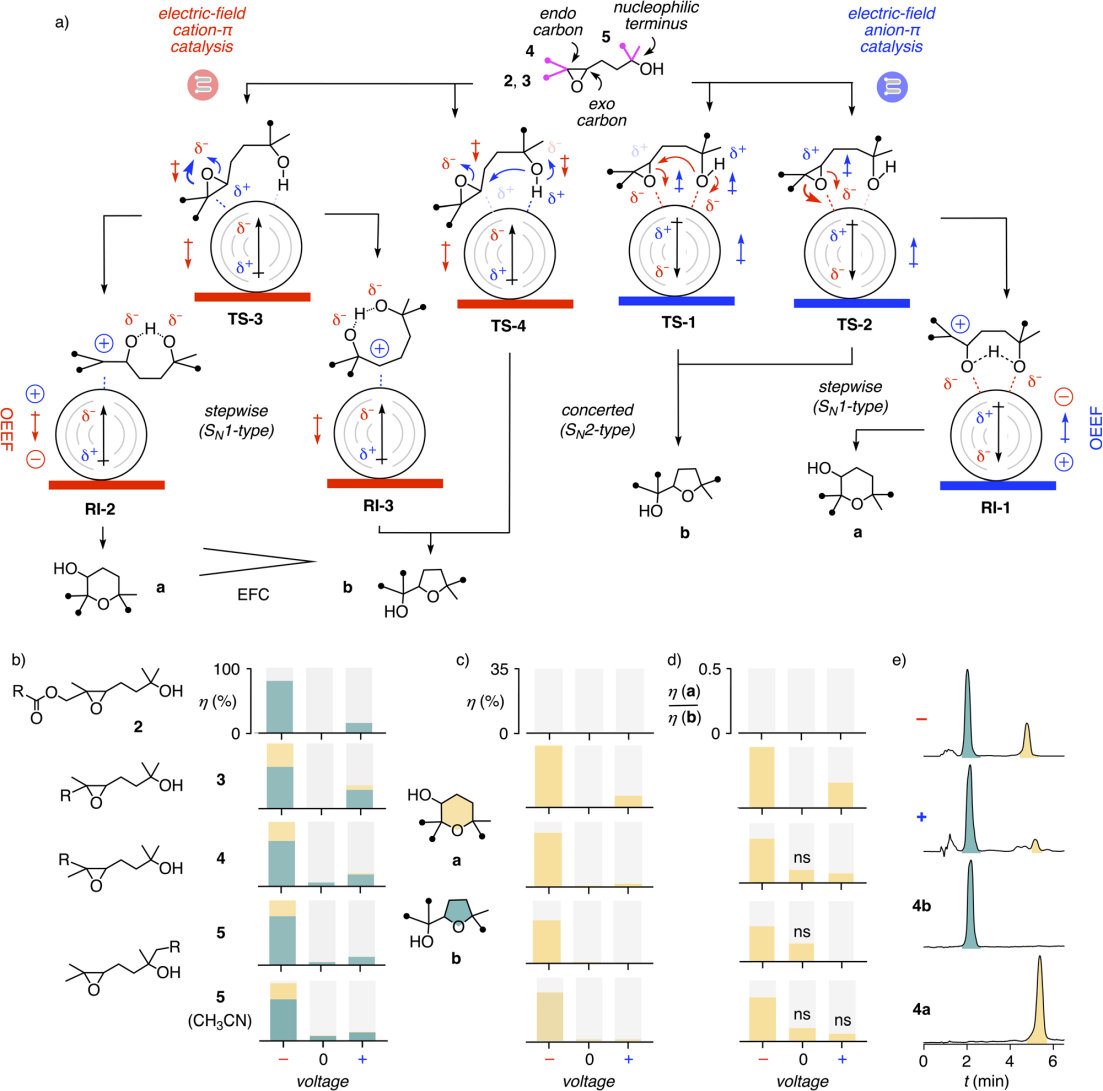
(a) Selected conceivable mechanisms to cyclize substrates 2–5 with cation–π (red) and anion–π (blue) EFC on MWCNTs in electromicrofluidic devices. Red/blue thick lines indicate graphite electrodes, concentric rings MWCNTs, with oriented macrodipoles induced by the OEEFs indicated as black arrows. Filled circles in molecules indicate either Me groups or pyrene interfacers in 2–5. (b) Dependence of conversion *η* and chemoselectivity a (yellow) *vs.*b (teal) on voltage applied to MWCNT-coated electromicrofluidic reactors (dry PC (except bottom: CH_3_CN), 50 mM (2) or 25 mM (3–5), 15 μL min^−1^, *V* ∼±3.0 V, see Fig. 3). (c) Voltage dependence of the yield of a. (d) Voltage dependence of the product ratio a/b. (ns) Due to very low yields, a/b ratios are not significant. (e) Representative HPLC traces of product mixtures obtained from 4 at negative and positive voltage compared to standard samples (top to bottom).

EFC combined with cation–π interactions could preferably stabilize carbocation intermediates as in RI-2 and, less preferred, RI-3. These intermediates are part of stepwise S_N_1-type mechanisms. They are accessed from TS-3, where the epoxide opens before the nucleophile reacts. The alternative concerted S_N_2-type mechanism in TS-4 was also conceivable with cation–π EFC. Deprotonation of the alcohol nucleophile by cation–π interactions could be harder, but the alcoholates in TS-4, repelled by the π-basic nanotube surfaces, would be more reactive than the ones stabilized by alcoholate–π interactions in TS-1 at inverted fields.

The previously reported EFC of epoxide-opening ether cyclization of 1 into only the Baldwin product 1b is likely to occur by concerted S_N_2-type mechanisms.^26^ To break the Baldwin rules, substrate 2 was considered first (Fig. 1c). Compared to the original 1, three methyls were added to inactivate the nucleophile and access tertiary carbocation intermediates like RI-1 and RI-2, and a cleavable ester was inserted in the tether to the pyrene interfacer (Fig. 1c and 2).

Anion–π catalysis on MWCNT suspensions in *o*-dichlorobenzene (ODCB) showed increasing conversion into 2b with increasing MWCNT concentration, reaching a rate enhancement re = 55 with 9 mol% MWCNTs (Fig. 3a). On MWCNTs in electromicrofluidic reactors, cyclizations failed without electric fields (Fig. 2b). With increasing applied voltage, the products started to emerge. Consistent with previous observations with EFC at STM tips^16^ and the importance of contributions from their EDL,^3^ conversions increased with solvent polarity, reaching ∼80% conversion in dry, polar aprotic propylene carbonate (PC) for one passage through the reactor at high negative voltage (Fig. 2b). Cation–π EFC under negative field gave a much higher conversion than anion–π EFC under positive field (Fig. 2b). Cation–π EFC could occur through either S_N_2-like TS-4 or the S_N_1-like TS-3 (Fig. 2a). The absence of anti-Baldwin product 2a suggested that the proximal ester destabilizes the tertiary carbocation in RI-2.

**Fig. 3:**
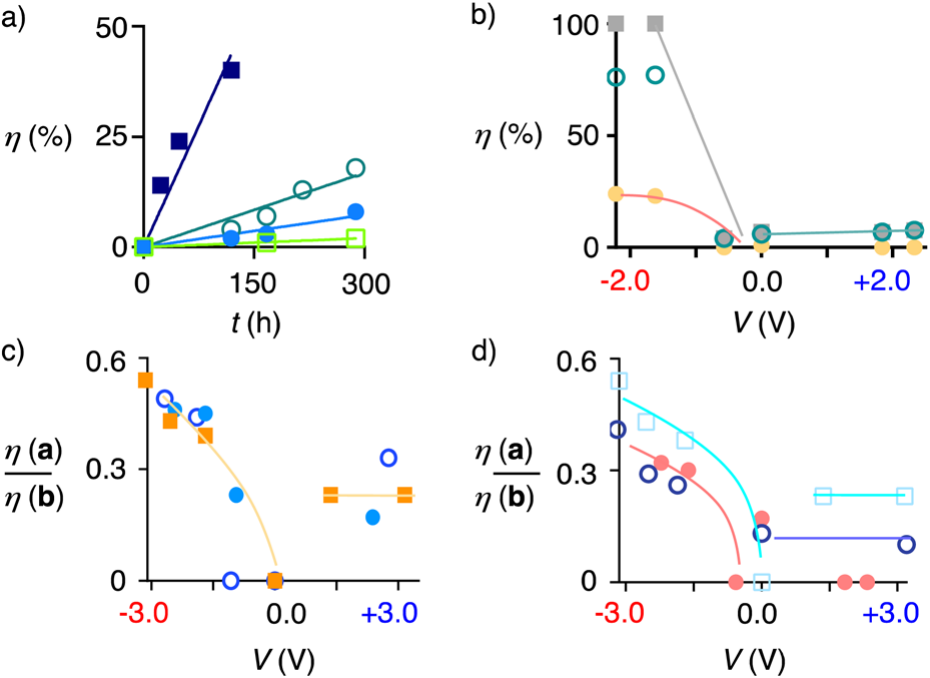
(a) Yield *η* of 2b with time obtained from 2 (100 mM) in the presence of 0 (□), 1 (●), 3 (○), and 9 wt% MWCNTs (■) suspended in ODCB, 40 °C, with linear fit. (b) Voltage dependence of the conversion *η* into 5a (●), 5b (○) and total conversion (■) as a function of external voltage, obtained from 5 (25 mM) in dry PC passing once through the electromicrofluidic reactor (15 μL min^−1^, Pt/Gr* electrodes, 250 μm apart). (c) Voltage dependence of the a/b ratio obtained from 3 in dry PC with 0 (■), 1.0 (●) and 10 (○) eq. of H_2_O. (d) Voltage dependence of the a/b ratio obtained from 3 (□), 4 (○) and 5 (●). Lines are added to guide the eye.

To promote access to RI-2 and enter into the anti-Baldwin region, the *cis*/*trans* isomers 3 and 4 without a cleavable ester in the tether to the pyrene interfacer were designed and synthesized (Schemes 1 and S1†). Results from EFC were similar for the two stereoisomers 3 and 4. Without EFC, cyclization was absent for 3 and negligible for 4 (Fig. 2b). Like for 2, cation–π EFC gave excellent conversion, reaching completion well above *V* ∼−3.0 V, while anion–π EFC was much less efficient, maximizing at *V* ∼+3.0 V with a conversion of *η* = 30% (Fig. 2b).

Unlike 2, electric-field catalyzed cyclization of 3 and 4 gave significant amounts of anti-Baldwin products 3a and 4a (Fig. 2b–d, yellow; Fig. 2e). As with Brønsted and Lewis acids, it has been exceptionally difficult to break the Baldwin rules with anion–π catalysis. Previous best was 10% anti-Baldwin product for the tetramethyl analog of 3 with small-molecule anion–π catalysts^57^ that operate with more complex mechanisms enhanced by autocatalysis.^58^ The 35%, obtained for 3 with cation–π EFC, slightly more than one-third of the total product, more than tripled this old record (Fig. 2c).

Most importantly, a/b-ratios increased significantly with increasing negative voltage (Fig. 3c and d). They were almost insensitive to the presence of water, which was important because water was shown to contribute to other mechanisms of ether cyclizations, including templation^59^ and autocatalysis^58^ (Fig. 3c). Increasing a/b-ratios with increasing voltage supported the idea that EFC on MWCNTs in electromicrofluidic devices can affect the reaction pathways. Namely, increasing cation–π EFC indeed appears to accelerate S_N_1-type cyclization through TS-3 and RI-2 selectively, consistent with a biomimetic^28,29^ stabilization of the tertiary carbocation by cation–π interactions (Fig. 2a).

Although overall much less powerful than above cation–π EFC with 3 and 4, anion–π EFC also provided small quantities of anti-Baldwin products 3a and 4a (Fig. 2b–d and 3d). These results implied field-induced access to TS-2 and RI-1 (Fig. 2a). This apparent shift from TS-1 to TS-2 could originate from the attachment of the pyrene interfacer to the epoxide terminus, strengthening anion–π interactions there and leaving the nucleophile terminus loose.

Tethering the interfacer to the nucleophile terminus could thus strengthen activation of the nucleophile, shift from TS-2 to TS-1 and thus suppress anti-Baldwin traces in anion–π EFC mode. To elaborate on this hypothesis, we designed and synthesized substrate 5 (Scheme S3†). Cation–π EFC was as dominant for 5 as for the other substrates 2–4 (Fig. 2b). The a/b-ratio increased with negative voltage (Fig. 2d and 3d). Consistent with a corresponding shift from TS-3 to TS-4 by nucleophile tethering, the a/b ratio for cation–π EFC of 5 was below that for 3 and similar to 4 (Fig. 2d and 3d). However, at high voltage, the a/b-ratio of 5 increased from PC to acetonitrile (Fig. 2d). These results supported that tighter tethering of the nucleophile rather than the epoxide might indeed shift the mechanism from TS-2 to TS-1 at positive and from TS-3 to TS-4 at negative voltage, lowering the anti-Baldwin product formation at both positive and negative voltage.

The quantitative reproducibility of individual values in experimental replicates was naturally limited, mainly due to differences in the MWCNT coatings. The persistent observation of increasing anti-Baldwin product formation with increasing voltage for different substrates was thus important also to document qualitative reproducibility (Fig. 3c and d). Other key trends, such as the fundamental switch from zero to full conversion by applying electric fields, were fully reproducible as well.

In summary, reaction mechanisms are shown to respond to electric-field catalysis on carbon nanotubes in electromicrofluidic devices. For epoxide-opening ether cyclizations, increasing stabilization of carbocation intermediates by cation–π EFC allows for a shift of the reaction mechanism and selective acceleration of the intrinsically disfavored pathways (here to break the Baldwin rules). Substrate positioning on the polarized aromatic surface is shown to enhance electric-field control over reaction mechanisms (here to suppress traces of anti-Baldwin products with anion–π EFC). These results support the potential of scalable EFC in microfluidic reactors, which opens many perspectives, from sustainable organic synthesis to the origin of life. Current emphasis is on replacement of MWCNTs by other carbon allotropes including unmodified graphite electrodes,^26^ catalyst immobilization and the engineering of Gouy–Chapman–Stern electrical double layers.

## Experimental section

See ESI.† Preliminary results on the topic have been published in a PhD thesis.^60^

## Data availability

Data for this paper are available at Zenodo at https://doi.org/10.5281/zenodo.15343823.

## Author contributions

M. A. G. L. and A. M. performed all synthesis and catalysis, N. S. and S. M. directed the study, all authors contributed to the design of experiments, data interpretation and manuscript writing.

## Conflicts of interest

There are no conflicts to declare.

## Supplementary Material

SC-OLF-D5SC02934A-s001
