# Supplementary material for: Response of reaction mechanisms to electric-field catalysis on carbon nanotubes in microfluidic reactors
Source: Chem Sci. 2025 May 28;16(25):11264–9. doi: 10.1039/d5sc02934a (PMC12117509; doi:10.1039/d5sc02934a)
Supplement: SC-016-D5SC02934A-s001 [file SC-016-D5SC02934A-s001.pdf]

Supplementary Information (SI) for Chemical Science.
This journal is © The Royal Society of Chemistry 2025

Electronic supplementary information

Response of reaction mechanisms to electric-field catalysis on carbon nanotubes in microfluidic reactors

Ángeles Gutiérrez López, Alenka Marsalek, Naomi Sakai, and Stefan Matile*

Department of Organic Chemistry, University of Geneva, CH-1211 Geneva, Switzerland

*Stefan.Matile@unige.ch

Table of contents

1.	Materials and methods	S3
2.	Synthesis	S4
	2.1. Synthesis of substrates	S4
	2.2. Synthesis of product references	S24
3.	Catalysis on MWCNTs in suspension	S31
4.	Microfluidic electric-field catalysis	S33
	4.1. General procedures	S33
	4.2. Dependence on solvents	S36
	4.3. Dependence on water	S38
	4.4. Dependence on substrates	S39
5.	Supplementary references	S42
6.	NMR spectra	S43

1. Materials and methods

As in reference S1. Reagents for synthesis were purchased from Merck, Apollo Scientific, Broadpharm, Sigma-Aldrich and Acros. Flash column chromatography was performed on a Biotage Isolera™ system. Analytical and preparative TLCs were performed on silica gel 60 F^{254} (Merck) and silica gel (SiliCycle, 1000 µm), respectively. Room temperature (RT) stands for 20-25 °C. Melting points (Mp) were measured on a Melting Point M-565 (BUCHI). IR spectra were recorded on a Perkin Elmer, FTIT spectrum two+ (ATR, Golden Gate) and are reported as wavenumbers ν in cm^{-1} with band intensities indicated as s (strong), m (medium), w (weak). ^1H and ^{13}C NMR were recorded (as indicated) either on a Bruker 300 MHz, 400 MHz, or 500 MHz spectrometer and are reported as chemical shifts (δ) in ppm relative to TMS ($\delta = 0$). Spin multiplicities are reported as a singlet (s), doublet (d), triplet (t) and quartet (q), with coupling constants (J) given in Hz, or multiplet (m). Broad peaks are marked as br. ESI-MS was measured using Advion expression CMS and Advion plate express TLC/CMS, reported as *m/z*. Accurate mass determinations using ESI (HR ESI-MS) were performed on Xevo G2-S Tof (Waters). Flow electrochemical experiments were performed using a stand-alone Vapourtec Ion Electrochemical Reactor, with an Aim-TTi EX354RD Dual Power Supply from Thurlbym Thandar Instruments Ltd. Chemyx Fusion 100 Touch Syringe Pumps was used in the flow set-ups. Electrode materials employed were platinum (Pt) and graphite (Gr) purchased from Goodfellow. The electrodes (5 x 5 cm^2) were separated by a 0.25 mm fluorinated ethylene propylene (FEP) spacer resulting in a reactor volume of 0.3 mL, with an exposed electrode surface area of 12 cm^2. All HPLC analyses were performed using a Jasco LC-4000 series HPLC system.

Abbreviations. *m*-CPBA: *meta*-Chloroperoxybenzoic acid; DCC: *N,N'*-dicyclohexylcarbodiimide; DMAP: 4-Dimethylaminopyridine; DMF: *N,N*-Dimethylformamide; DMP: Dess-Martin periodinane; LiHMDS: Lithium bis(trimethylsilyl)amide; MWCNT: Multi-walled carbon nanotube; ODCB: *o*-dichlorobenzene; PC: Propylene carbonate; RT: Room temperature; TBAF: Tetra-*n*-butylammonium fluoride; TBDPSCl: *tert*-Butyl(chloro)diphenylsilane; THF: Tetrahydrofuran; TMSCl: Trimethylchlorosilane.

2. Synthesis

2.1. Synthesis of substrates

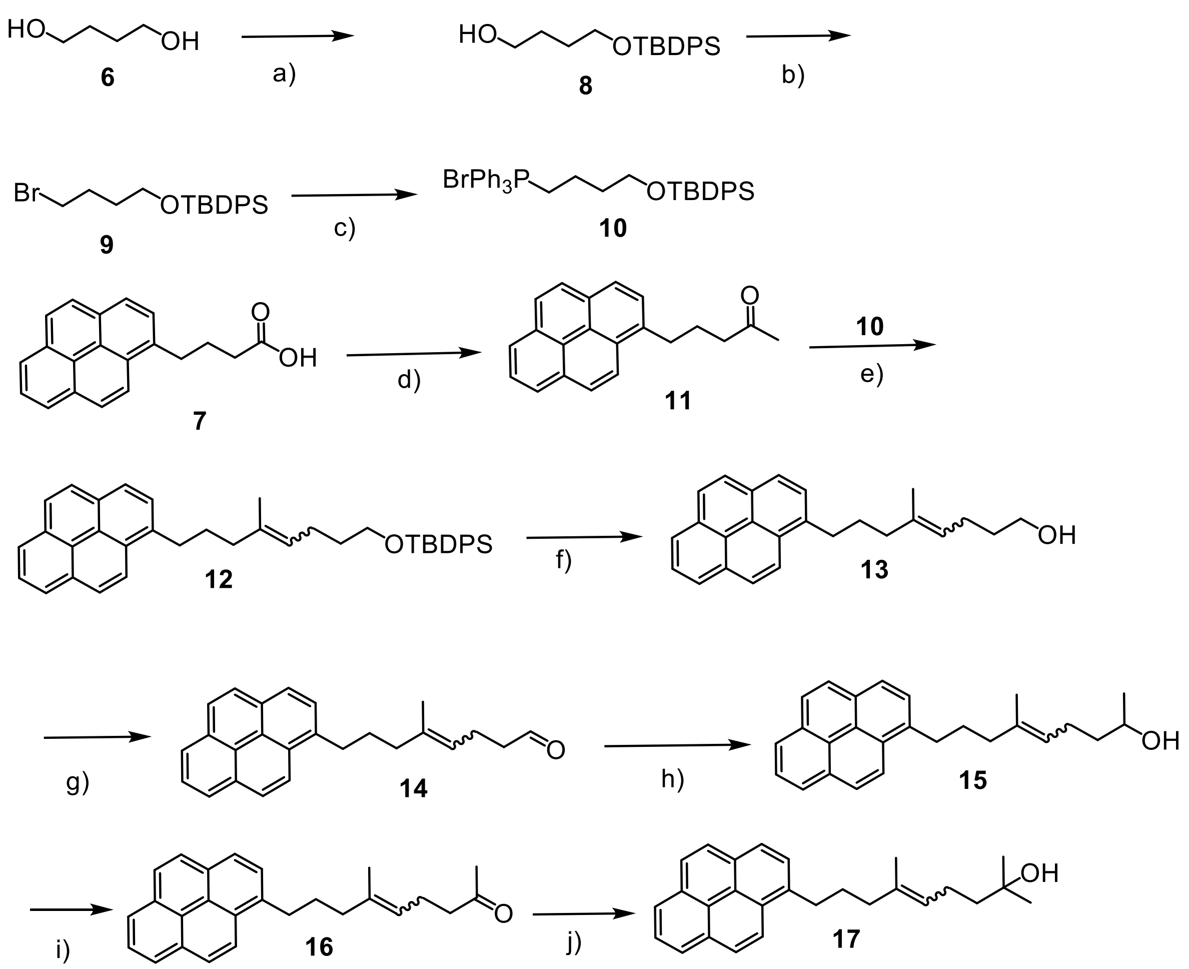

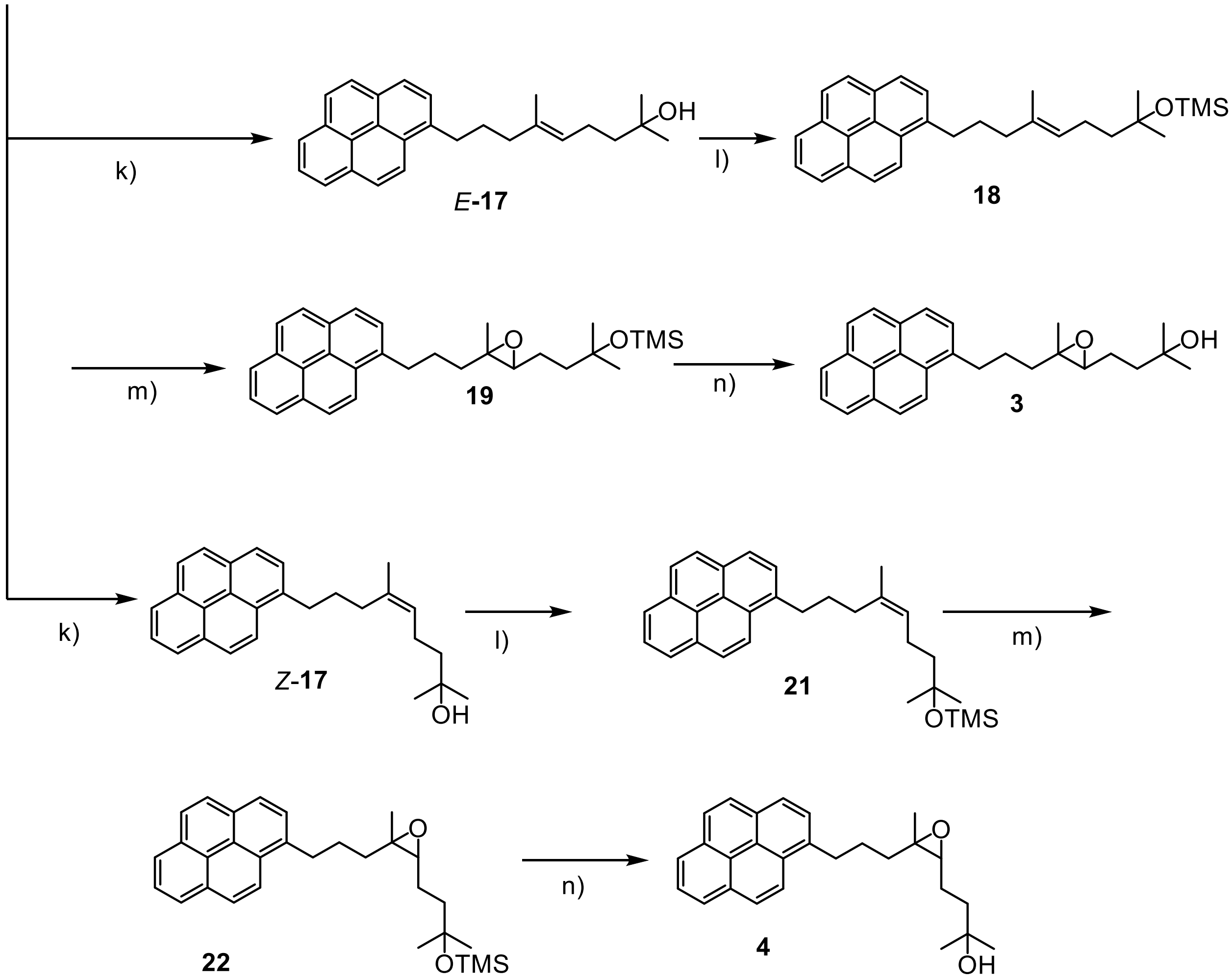

Scheme S1 Synthesis of substrates **3** and **4**. (a) 1. NaH, THF, 0 °C, 30 min; 2. TBDPSCl, THF, 0 °C, 2 h, quant. (b) PPh_3, CBr_4, CH_2Cl_2, 0 °C to RT, 2 h, 56%. (c) PPh_3, toluene, 150 °C, 15 h, 56%. (d) MeLi, THF, -78 °C to RT, 3 h, 70%. (e) 1. **10**, LiHDMS, THF, -78 °C to 0 °C, 30 min; 2. **11**, -78 °C to RT, 15 h, 40%. (f) TBAF, THF, 0 °C to RT, 2 h, 91%. (g) DMP, CH_2Cl_2, 0 °C to RT, 3 h, 61%. (h) MeMgBr, dry Et_2O, 0 °C to RT, 1 h, quant. (i) DMP, CH_2Cl_2, 0 °C to RT, 3 h, 78%. (j) MeMgBr, dry Et_2O, 0 °C to RT, 1 h, 83%. (k) preparative HPLC. (l) DMAP, Et_3N, TMSCl, CH_2Cl_2, RT, 1 h, 82% (**18**), 78% (**21**). (m) *m*-CPBA, CH_2Cl_2, 0 °C to RT, 1 h, 83% **19**, 87% **22**. (n) TBAF, THF, 0 °C to RT, 2 h, 94% **3**, 99% **4**.

Compound 8 was prepared following the reported procedures.[S2]

Compound 9 was prepared following the reported procedures.[S3]

Compound 10 was prepared following the reported procedures.[S4]

Compound 11. A solution of MeLi in THF (1.3 M, 17 mL, 27 mmol) was added dropwise to a solution of **7** (4.00 g, 13.5 mmol) in anhydrous THF (55 mL) at –78 °C under Ar atmosphere. The mixture was stirred for 40 min at the same temperature and then for 2 h at RT. The crude mixture was quenched with sat. aqueous NH_4Cl (50 mL) and then extracted with EtOAc (3 × 50 mL). The organic phase was washed with brine (2 × 50 mL), dried over Na_2SO_4, filtered, and concentrated under vacuum. Purification by flash column chromatography (*n*-pentane/EtOAc 9:1) gave **11** as a colorless solid (2.70 g, 70%). R_f (*n*-pentane/EtOAc 9:1): 0.6; Mp: 85 – 86 °C; IR (neat): 3043 (w, C-H), 2887 (m, C-H), 1708 (s, C=O), 1433 (m, C-H), 1351 (m), 1157 (m, C-O); ^{1}H NMR (500 MHz, CD_2Cl_2): 8.35 (d, $^3J_{H-H}$ = 9.5 Hz, 1H), 8.20 – 8.17 (m, 2H), 8.16 (d, $^3J_{H-H}$ = 9.5 Hz, 1H), 8.14 (d, $^3J_{H-H}$ = 7.5 Hz, 1H), 8.06 (d, $^3J_{H-H}$ = 9.1 Hz, 1H), 8.04 (d, $^3J_{H-H}$ = 9.1 Hz, 1H), 8.01 (t, $^3J_{H-H}$ = 7.5 Hz, 2H), 7.88 (d, $^3J_{H-H}$ = 7.5 Hz, 1H), 3.37 – 3.33 (m, 2H), 2.57 (t, $^3J_{H-H}$ = 7.2 Hz, 2H), 2.15 – 2.07 (m, 2H), 2.11 (s, 3H); ^{13}C NMR (126 MHz, CD_2Cl_2): 207.8 (C=O), 136.8 (C), 131.8 (C), 131.3 (C), 130.3 (C), 129.1 (C), 127.9 (CH), 127.8 (CH), 127.6 (CH), 127.0 (CH), 126.3 (CH), 125.4 (C), 125.3 (C), 125.3 (CH), 125.2 (CH), 125.1 (CH), 123.9 (CH), 43.1 (CH_2), 33.0 (CH_2), 30.1 (CH_2), 25.6 (CH_3); MS (ESI): 309 ($[M+Na]^+$).

Compound 12. Compound **10** (2.8 g, 4.9 mmol) was dissolved in dry THF (36 mL) in an oven-dried Schlenk flask. The flask was evacuated and back-filled with nitrogen for 3 times. Then, the solution was cooled to –78 °C and LiHMDS solution in THF (1.0 M, 10 mL, 10 mmol) was added dropwise via syringe. The mixture was stirred at 0 °C for 30 min followed by the dropwise addition of **11** (1.2 g, 4.1 mmol) at –78 °C. The mixture was warmed up and stirred for 15 h at RT. The reaction mixture was quenched with saturated aqueous NH_4Cl (15 mL), and extracted with EtOAc (3 x 10 mL). The combined organic phases were dried over Na_2SO_4 and concentrated under vacuum. Further purification by flash column chromatography (*n*-pentane/EtOAc 94:6) gave **12** (950 mg, 40%) as a

mixture of *E*/*Z* (≈1:1) isomers. R_f (*n*-pentane/EtOAc 94:6): 0.60; IR (neat): 3042 (w, C-H), 2930 (m, C-H), 1588 (w, C=C), 1427 (w, C-H), 1110 (s, C-O); ^{1}H NMR (500 MHz, CD_2Cl_2; *nn*/*nn* stereo-isomeric peaks): 8.29/8.27 (d, $^3J_{H-H}$ = 7.2 Hz, 1H), 8.18/8.17 (d, $^3J_{H-H}$ = 7.6 Hz, 2H), 8.14 – 8.08 (m, 2H), 8.07 – 8.02 (m, 2H), 8.00/7.99 (t, $^3J_{H-H}$ = 7.5 Hz, 1H), 7.89/7.87 (d, $^3J_{H-H}$ = 7.6 Hz, 1H), 7.68 – 7.64 (m, 4H), 7.40 – 7.32 (m, 6H), 5.21 (t, $^3J_{H-H}$ = 7.2 Hz, 1H), 3.68 (t, $^3J_{H-H}$ = 6.3 Hz, 2H), 3.33 – 3.28 (m, 2H), 2.28 – 2.12 (m, 4H), 1.97 – 1.90 (m, 2H), 1.72/1.64 (s, 3H), 1.66 – 1.61 (m, 2H), 1.05/1.03 (s, 9H); ^{13}C NMR (126 MHz, CD_2Cl_2; *nn*/*nn* stereo-isomeric peaks): 137.9/137.7 (C), 136.0 (2CH), 136.0 (2CH), 135.7/135.5 (C), 134.6 (C), 134.5 (C), 134.2/134.0 (CH), 131.8/131.4 (C), 130.1/130.0 (C), 129.9 (CH), 129.9 (CH), 129.2/128.9 (CH), 128.0 (2CH), 128.0 (CH), 127.9 (CH), 127.8 (CH), 127.6 (CH), 127.5/127.4 (CH), 126.8 (CH), 126.2 (CH), 125.7 (CH), 125.4 (C), 125.2 (CH), 125.1 (CH), 125.0 (CH), 125.0 (CH), 124.0/123.9 (CH), 64.0/63.9 (CH_2), 40.1/32.3 (CH_2), 33.7/33.2 (CH_2), 33.4 (CH_2), 30.7/30.6 (CH_2), 27.1/27.0 ($3CH_3$), 24.7/24.6 (CH_2), 23.5/16.1 (CH_3), 19.5/19.5 (C); MS (ESI): 603 ($[M+Na]^+$).

Compound 13. To a solution of **12** (950 mg, 1.6 mmol) in dry THF (58 mL), a solution of TBAF in THF (1.0 M, 2.1 mL, 2.1 mmol) was added dropwise at 0 °C. The mixture was stirred at RT for 2 h. Afterward, the mixture was concentrated and purified by flash column chromatography (*n*-pentane/EtOAc 4:1) to give **13** (510 mg, 91%) as a colorless oil. R_f (*n*-pentane/EtOAc 4:1): 0.40; IR (neat): 3338 (w, O-H), 3039 (w, C-H), 2931 (s, C-H), 1602 (m, C=C), 1434 (s, C-H), 1055 (s, C-O); ^{1}H NMR (500 MHz, CD_2Cl_2; *nn*/*nn* stereo-isomeric peaks): 8.30/8.28 (d, $^3J_{H-H}$ = 9.3 Hz, 1H), 8.18 – 8.15 (m, 2H), 8.13 – 8.10 (m, 2H), 8.05 – 8.01 (m, 2H), 7.99/7.99 (t, $^3J_{H-H}$ = 7.7 Hz, 1H), 7.90/7.88(d, $^3J_{H-H}$ = 7.8 Hz, 1H), 5.26 – 5.19 (m, 1H), 3.60/3.53 (t, $^3J_{H-H}$ = 6.6 Hz, 2H), 3.35 – 3.29 (m, 2H), 2.25/2.19 (t, $^3J_{H-H}$ = 7.6 Hz, 2H), 2.08 – 2.03 (m, 2H), 1.98 – 1.91 (m, 2H), 1.73/1.65 (d, $^4J_{H-H}$ = 1.3 Hz, 3H), 1.62 – 1.51 (m, 2H); ^{13}C NMR (126 MHz, CD_2Cl_2; *nn*/*nn* stereo-isomeric peaks): 137.8/137.6 (C), 136.0/135.8 (C), 131.8 (C), 131.3 (C), 130.1/130.1 (C), 129.0 (C), 127.9 (CH), 127.7/127.7 (CH), 127.5/127.4 (CH), 126.9/126.8 (CH), 126.2/126.2 (CH), 125.5/124.8 (CH), 125.4 (C), 125.3 (C), 125.2 (CH), 125.2/125.2 (CH), 125.0/125.0 (CH), 123.9/123.9 (CH), 62.9/62.8 (CH_2),

40.1/32.2 (CH_2), 33.7/33.4 (CH_2), 33.5/33.2 (CH_2), 30.6/30.6 (CH_2), 24.7/24.6 (CH_2), 23.6/16.1 (CH_3).

Compound 14. Compound **13** (950 mg, 2.8 mmol) was dissolved in CH_2Cl_2 (14 mL) and DMP (1.3 mL, 4.2 mmol) was added portion wised at 0 °C. Afterward, the mixture was warmed to RT and stirred for 3 h. The mixture was diluted with CH_2Cl_2 (14 mL) and quenched with sat. aqueous $NaHCO_3$ (10 mL). Then, the organic phase was washed with sat. aqueous $Na_2S_2O_3$ solution (2 x 10 mL) and brine (2 x 10 mL). The organic layer was dried over Na_2SO_4, filtered and concentrated under vacuum. The crude residue was purified by flash column chromatography (*n*-pentane/EtOAc 9:1) to give **14** (570 mg, 61%). R_f (*n*-pentane/EtOAc 9:1): 0.70; IR (neat): 3040 (w, C-H), 2934 (m, C-H), 1722 (s, C=O), 1603 (w, C=C), 1435 (w, C-H); ^{1}H NMR (500 MHz, CD_2Cl_2; *nn*/*nn* stereo-isomeric peaks): 9.74/9.68 (t, $^3J_{H\text{-}H}$ = 1.7 Hz, 1H), 8.30/8.29 (d, $^3J_{H\text{-}H}$ = 9.0 Hz, 1H), 8.20 – 8.16 (m, 2H), 8.14 – 8.11 (m, 2H), 8.06 – 8.03 (m, 2H), 8.00/8.00 (t, $^3J_{H\text{-}H}$ = 7.8 Hz, 1H), 7.91/7.89 (d, $^3J_{H\text{-}H}$ = 7.7 Hz, 1H), 5.22 – 5.16 (m, 1H), 3.36 – 3.29 (m, 2H), 2.48 – 2.39 (m, 2H), 2.37 – 2.31 (m, 2H), 2.29 – 2.26/2.21 – 2.18 (m, 2H), 2.01 – 1.92 (m, 2H), 1.74/1.68 (d, $^4J_{H\text{-}H}$ = 1.3 Hz, 3H); ^{13}C NMR (126 MHz, CD_2Cl_2; *nn*/*nn* stereo-isomeric peaks): 202.8/202.7 (C=O), 137.7/137.5 (C), 137.1/136.8 (C), 131.8 (C), 131.3 (C), 130.1/130.1 (C), 129.0 (C), 127.9 (CH), 127.7/127.6 (CH), 127.5/127.4 (CH), 126.9/126.8 (CH), 126.3/126.2 (CH), 125.4 (C), 125.3 (C), 125.2 (2CH), 125.1/125.0 (CH), 124.0/123.1 (CH), 123.9/123.8 (CH), 44.5/44.3 (CH_2), 40.0/32.2 (CH_2), 33.7/33.4 (CH_2), 30.5/30.4 (CH_2), 23.5/16.1 (CH_3), 21.2/21.1 (CH_2); MS (ESI): 363 ($[M+Na]^+$).

Compound 15. To a solution of **14** (570 mg, 1.7 mmol) in dry Et_2O (8 mL), a solution of MeMgBr in Et_2O (3.0 M, 1.1 mL, 3.3 mmol) was added dropwise at 0 °C. After stirring at RT for 1 h, the reaction mixture was quenched with sat. aqueous NH_4Cl (2 mL) and extracted with CH_2Cl_2 (3 x 2 mL). The combined organic phase was washed with brine (2 x 2 mL), dried over Na_2SO_4 and concentrated under vacuum to give **15** (600 mg, quantitative) as a colorless oil. R_f (*n*-pentane/Et_2O 4:1): 0.25; IR (neat): 3337 (w, O-H), 3039 (w, C-H), 2931 (s, C-H), 1602 (w, C=C), 1433 (m, C-H), 1055 (s, C-O); ^{1}H NMR (500 MHz, CD_2Cl_2; *nn*/*nn* stereo-isomeric peaks): 8.30/8.28 (d, $^3J_{H\text{-}H}$ = 9.2

Hz, 1H), 8.18 – 8.15 (m, 2H), 8.13 – 8.10 (m, 2H), 8.03 – 8.01 (m, 2H), 7.98 (t, $^3J_{H\text{-}H}$ = 7.4 Hz, 1H), 7.91/7.90 (d, $^3J_{H\text{-}H}$ = 9.3 Hz, 1H), 5.25 – 5.19 (m, 1H), 3.79 – 3.51 (m, 1H), 3.35 – 3.29 (m, 2H), 2.25/2.19 (t, $^3J_{H\text{-}H}$ = 7.7 Hz, 2H), 2.14 – 2.01 (m, 2H), 1.99 – 1.91 (m, 2H), 1.72/1.66 (s, 3H), 1.49 – 1.35 (m, 2H), 1.17 – 1.14/1.09 – 1.06 (m, 3H); ^{13}C NMR (126 MHz, CD_2Cl_2; *nn*/*nn* stereo-isomeric peaks): 137.8/137.6 (C), 135.8/135.6 (C), 131.8 (C), 131.4 (C), 130.1/130.1 (C), 129.0 (C), 127.9 (CH), 127.7/127.7 (CH), 127.5/127.4 (CH), 126.9/126.8 (CH), 126.2 (CH), 125.6/125.4 (CH), 125.4 (C), 125.3 (C), 125.2 (CH), 125.2 (CH), 125.0/125.0 (CH), 123.9/123.9 (CH), 68.1/62.9 (CH), 40.1/32.2 (CH_2), 40.0/39.7 (CH_2), 33.7/33.4 (CH_2), 30.6/30.5 (CH_2), 24.8/24.7 (CH_2), 23.7/23.7 (CH_3), 23.6/16.1 (CH_3).

Compound 16. Compound **15** (600 mg, 1.7 mmol) was dissolved in CH_2Cl_2 (9.0 mL) and DMP (1.1 g, 2.5 mmol) was added portionwise at 0 °C and the mixture was warmed to RT and stirred for 3 h. Afterward, the mixture was diluted with CH_2Cl_2 (9.0 mL) and quenched with sat. aqueous $NaHCO_3$ (9 mL). Then the organic phase was washed with sat. aqueous $Na_2S_2O_3$ solution (2 x 9 mL) and brine (1 x 9 mL). The organic phase was dried over Na_2SO_4, filtered and concentrated under vacuum. The residue was purified by flash column chromatography (*n*-pentane/EtOAc 4:1) to obtain **16** (520 mg, 87%). R_f (*n*-pentane/EtOAc 4:1): 0.30; IR (neat): 3039 (w, C-H), 2932 (s, C-H), 1713 (s, C=O), 1602 (w, C=C), 1434 (m, C-H); ^{1}H NMR (500 MHz, CD_2Cl_2; *nn*/*nn* stereo-isomeric peaks): 8.30/8.28 (d, $^3J_{H\text{-}H}$ = 8.5 Hz, 1H), 8.19 – 8.16 (m, 2H), 8.14 – 8.11 (m, 2H), 8.06 – 8.02 (m, 2H), 8.00 (d, $^3J_{H\text{-}H}$ = 7.6 Hz, 2H), 7.89 (t, $^3J_{H\text{-}H}$ = 7.8 Hz, 1H), 5.18 – 5.13 (m, 1H), 3.35 – 3.28 (m, 2H), 2.45/2.40 (t, $^3J_{H\text{-}H}$ = 7.5 Hz, 2H), 2.28 – 2.17 (m, 4H), 2.08/2.02 (s, 3H), 1.96 – 1.91 (m, 2H), 1.72/1.66 (s, 3H); ^{13}C NMR (126 MHz, CD_2Cl_2; *nn*/*nn* stereo-isomeric peaks): 208.7/208.6 (C=O), 137.7/137.5 (C), 136.5/136.3 (C), 131.8 (C), 131.3 (C), 130.1/130.0 (C), 129.0 (C), 127.9 (CH), 127.7/127.6 (CH), 127.5/127.4 (CH), 126.8/126.8 (CH), 126.2 (CH), 125.3/125.3 (CH), 125.2 (C), 125.2 (C), 125.1 (CH), 125.0 (CH), 124.3/123.7 (CH), 123.9/123.8 (CH), 44.1/43.9 (CH_2), 40.0/32.1 (CH_2), 33.7/33.3 (CH_2), 30.6/30.4 (CH_2), 30.0/30.0 (CH_3), 23.5/16.0 (CH_3), 22.8/22.6 (CH_2), 14.4 (CH_3).

Compound 17. To a solution of **16** (430 mg, 1.20 mmol) in dry Et_2O (5.7 mL), a solution of MeMgBr in Et_2O (3.0 M, 0.8 mL, 2.4 mmol) was added at 0 °C. Afterward, the mixture was warmed up to RT and stirred for 1 h. The reaction mixture was quenched with sat. aqueous NH_4Cl (2 mL) and extracted with CH_2Cl_2 (3 x 2 mL). The combined organic phase was washed with brine (2 x 2 mL), dried over Na_2SO_4 and concentrated under vacuum. to give **17** (370 mg, 83%) was obtained as a colorless oil. R_f (*n*-pentane/EtOAc 4:1): 0.3; IR (neat): 3382 (w, O-H), 3040 (w, C-H), 2930 (s, C-H), 1603 (w, C=C), 1459 (m, C-H), 1182 (m, C-O); MS (ESI): 393 ($[M+Na]^+$).

Compounds (*E*)-17 and (*Z*)-17. Stereoisomers of compound **17** (370 mg, 1.0 mmol) were separated by preparative HPLC (CHIRALPAK® IA (20 x 250 mm), 12.8 mL/min, 15% EtOAc in *n*-hexane) to give compounds (*E*)-**17** (R_t ~ 12 min, 185 mg) and (*Z*)-**17** (R_t ~ 10 min, 170 mg) as colorless oils.

(*E*)-17. ^{1}H NMR (500 MHz, CD_2Cl_2): 8.30 (d, $^3J_{H\text{-}H}$ = 9.3 Hz, 1H), 8.22 – 8.16 (m, 2H), 8.14 – 8.09 (m, 2H), 8.05 (d, $^3J_{H\text{-}H}$ = 8.9 Hz, 1H), 8.03 (d, $^3J_{H\text{-}H}$ = 8.9 Hz, 1H), 8.02 – 7.98 (m, 1H), 7.90 (d, $^3J_{H\text{-}H}$ = 7.8 Hz, 1H), 5.27 – 5.22 (m, 1H), 3.35 – 3.28 (m, 2H), 2.19 (t, $^3J_{H\text{-}H}$ = 7.5 Hz, 2H), 2.13 – 2.07 (m, 2H), 1.99 – 1.91 (m, 2H), 1.67 (q, $^4J_{H\text{-}H}$ = 0.9 Hz, 3H), 1.52 – 1.47 (m, 2H), 1.19 (s, 6H); ^{13}C NMR (126 MHz, CD_2Cl_2): 137.8 (C), 135.3 (C), 131.8 (C), 131.4 (C), 130.1 (C), 129.0 (C), 127.9 (CH), 127.7 (CH), 127.4 (CH), 126.8 (CH), 126.2 (CH), 125.4 (CH), 125.4 (C), 125.3 (C), 125.2 (CH), 125.2 (CH), 125.0 (CH), 124.0 (CH), 71.0 (C), 44.1 (CH_2), 40.0 (CH_2), 33.4 (CH_2), 30.5 (CH_2), 29.4 ($2CH_3$), 23.4 (CH_2), 16.0 (CH_3).

(*Z*)-17. ^{1}H NMR (500 MHz, CD_2Cl_2): 8.31 (d, $^3J_{H\text{-}H}$ = 9.3 Hz, 1H), 8.20 – 8.16 (m, 2H), 8.15 – 8.11 (m, 2H), 8.05 (d, $^3J_{H\text{-}H}$ = 9.0 Hz, 1H), 8.04 (d, $^3J_{H\text{-}H}$ = 9.0 Hz, 1H), 8.00 (t, $^3J_{H\text{-}H}$ = 7.6 Hz, 1H), 7.91 (d, $^3J_{H\text{-}H}$ = 7.8 Hz, 1H), 5.24 – 5.17 (m, 1H), 3.38 – 3.28 (m, 2H), 2.29 – 2.22 (m, 2H), 2.06 – 1.99 (m, 2H), 1.99 – 1.91 (m, 2H), 1.74 (q, $^4J_{H\text{-}H}$ = 1.3 Hz, 3H), 1.46 – 1.39 (m, 2H), 1.08 (s, 6H); ^{13}C NMR (126 MHz, CD_2Cl_2): 137.6 (C), 135.5 (C), 131.8 (C), 131.3(C), 130.1 (C), 129.0 (C), 127.9 (CH), 127.7 (CH), 127.5 (CH), 126.9 (CH), 126.2 (CH), 126.0 (CH), 125.4 (C), 125.3 (C), 125.2

(2CH), 125.0 (CH), 123.9 (CH), 70.9 (C), 44.4 (CH_2), 33.7 (CH_2), 32.1 (CH_2), 30.6 (CH_2), 29.3 ($2CH_3$), 23.6 (CH_3), 23.3 (CH_2).

a)

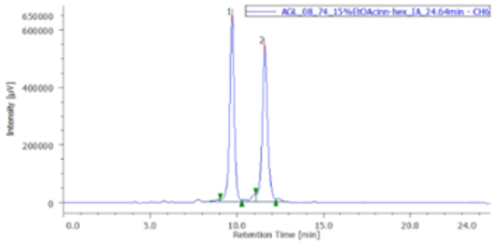

#	Peak Name	CH	tR [min]	Area [µV·sec]	Height [µV]	Area%	Height%
1	Unknown	6	9.717	11710481	647725	49.687	54.421
2	Unknown	6	11.600	11858243	542488	50.313	45.579

b)

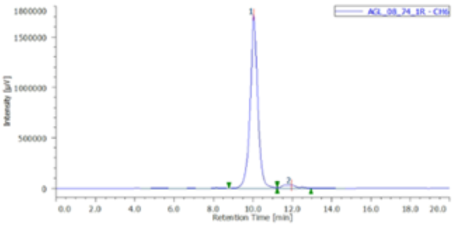

#	Peak Name	CH	tR [min]	Area [µV·sec]	Height [µV]	Area%	Height%
1	Unknown	6	10.033	45897276	1720272	96.467	98.229
2	Unknown	6	11.950	1681173	31016	3.533	1.771

c)

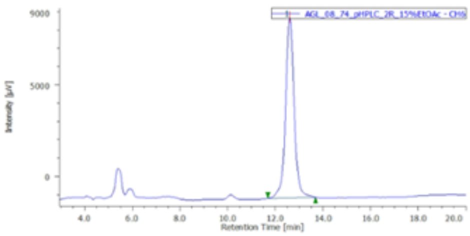

#	Peak Name	CH	tR [min]	Area [µV·sec]	Height [µV]	Area%	Height%
1	Unknown	6	12.600	247941	9869	100.000	100.000

Fig. S1 Analytical HPLC chromatograms of compounds (a) **17**, (b) (*Z*)-**17**, and (c) (*E*)-**17**; CHIRALPAK® IA (4.6 x 250 mm), 0.8 mL/min, 15% EtOAc in *n*-hexane, λ_{abs} = 254 nm.

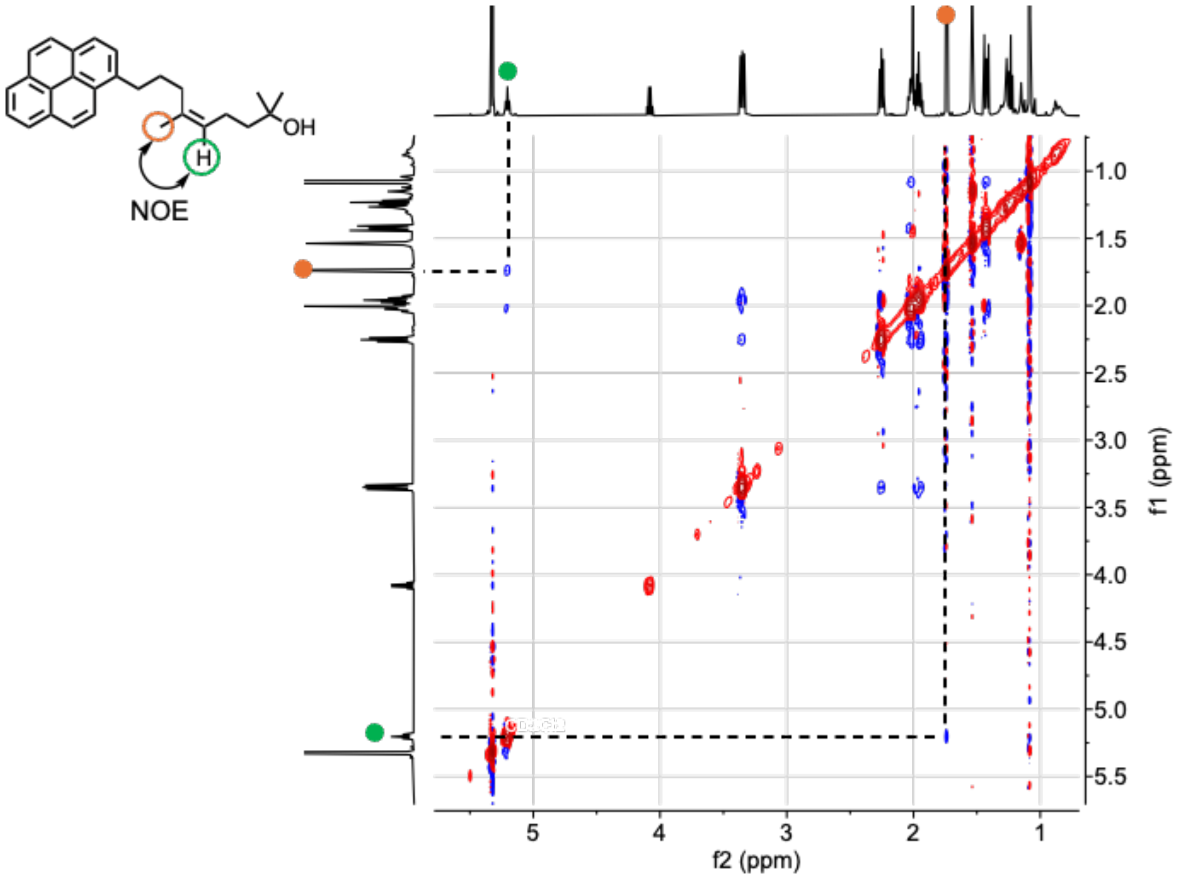

Fig. S2 NOESY spectrum (500 MHz, CD_2Cl_2) of (*Z*)-**17**. Correlations were found between the vinyl proton peak (green) and the vinyl methyl peak (orange), providing evidence for (*Z*)-configuration.

Compound 18. To a solution of (*E*)-**17** (370 mg, 0.99 mmol) and DMAP (12 mg, 98 µmol) in CH_2Cl_2 (4.5 mL) at RT under Ar atmosphere, Et_3N (420 µL, 3.0 mmol) was added followed by TMSCl (0.26 mL, 2.0 mmol). The mixture was stirred at RT for 1 h. Then, water (5 mL) was added to the mixture, and the phases were separated. The organic phase was washed with water (2 x 5 mL) and aqueous HCl solution (1 M, 2 x 5 mL). The organic phase was dried over Na_2SO_4, filtered and concentrated to dryness under reduced pressure to yield **18** (340 mg, 78%) as a colorless oil. R_f (*n*-pentane/EtOAc 4:1): 0.65; IR (neat): 3041 (w, C-H), 2965 (s, C-H), 1603 (w, C=C), 1458 (m, C-H), 1248 (s, C-O), 1048 (s, C-O); ^{1}H NMR (500 MHz, CD_2Cl_2): 8.30 (d, $^3J_{H\text{-}H}$ = 9.3 Hz, 1H), 8.18 (d, $^3J_{H\text{-}H}$ = 7.6 Hz, 1H), 8.17 (d, $^3J_{H\text{-}H}$ = 7.6 Hz, 1H), 8.13 (d, $^3J_{H\text{-}H}$ = 7.7 Hz, 1H), 8.11 (d, $^3J_{H\text{-}H}$ = 9.3 Hz, 1H), 8.04 (d, $^3J_{H\text{-}H}$ = 9.0 Hz, 1H), 8.03 (d, $^3J_{H\text{-}H}$ = 9.0 Hz, 1H), 7.99 (t, $^3J_{H\text{-}H}$ = 7.6 Hz, 1H), 7.91 (d, $^3J_{H\text{-}H}$ = 7.7 Hz, 1H), 5.25 – 5.22 (m, 1H), 3.34 – 3.30 (m, 2H), 2.19 (t, $^3J_{H\text{-}H}$ = 7.4 Hz, 2H), 2.11 – 2.05 (m, 2H), 2.00 – 1.92 (m, 2H), 1.66 (s, 3H), 1.53 – 1.45 (m, 2H), 1.22 (s, 6H), 0.10 (s, 9H); ^{13}C

NMR (126 MHz, CD_2Cl_2): 138.6 (C), 134.7 (C), 131.8 (C), 131.4 (C), 130.1 (C), 129.0 (C), 127.9 (CH), 127.8 (CH), 127.4 (CH), 126.8 (CH), 126.2 (CH), 125.8 (CH), 125.4 (CH), 125.3 (CH), 125.2 (C), 125.1 (C), 125.0 (CH), 124.0 (CH), 74.3 (C), 45.1 (CH_2), 40.1 (CH_2), 33.4 (CH_2), 30.6 (CH_2), 29.9 ($2CH_3$), 23.4 (CH_2), 16.0 (CH_3), 4.1 ($3CH_3$); MS (ESI): 465 ($[M+Na]^+$).

Compound 21. To a solution of (*Z*)-**17** (332 mg, 0.90 mmol) and DMAP (11 mg, 900 mmol) in CH_2Cl_2 (4.0 mL) under Ar atmosphere, Et_3N (380 µL, 2.7 mmol) was added, followed by dropwise addition of TMSCl (0.23 mL, 1.8 mmol) and the mixture was stirred at RT for 1 h. Then, water (5 mL) was added, and the phases were separated. The organic phase was washed with water (2 x 5 mL) and 1 M aqueous solution HCl (2 x 5 mL). The organic phase was dried over Na_2SO_4, filtered and the solvent removed under reduced pressure to yield **21** (320 mg, 82%) as a colorless oil. R_f (*n*-pentane/EtOAc 4:1): 0.65; IR (neat): 3040 (w, C-H), 2963 (s, C-H), 1603 (w, C=C), 1453 (m, C-H), 1247 (s, C-O), 1046 (s, C-O); ^{1}H NMR (500 MHz, CD_2Cl_2): 8.30 (d, $^3J_{H-H}$ = 9.2 Hz, 1H), 8.19 – 8.16 (m, 2H), 8.13 (d, $^3J_{H-H}$ = 7.7 Hz, 1H), 8.11 (d, $^3J_{H-H}$ = 9.2 Hz, 1H), 8.05 (d, $^3J_{H-H}$ = 9.0 Hz, 1H), 8.00 (d, $^3J_{H-H}$ = 9.0 Hz, 1H), 7.99 (t, $^3J_{H-H}$ = 7.7 Hz, 1H), 7.91 (d, $^3J_{H-H}$ = 7.7 Hz, 1H), 5.22 – 5.19 (m, 1H), 3.36 – 3.33 (m, 2H), 2.27 – 2.24 (m, 2H), 2.06 – 2.01 (m, 2H), 1.98 – 1.92 (m, 2H), 1,73 (d, $^4J_{H-H}$ = 1.3 Hz, 3H), 1.44 – 1.42 (m, 2H), 1.14 (s, 6H), 0.08 (s, 9H); ^{13}C NMR (126 MHz, CD_2Cl_2): 137.7 (C), 135.0 (C), 131.8 (C), 131.4 (C), 130.1 (C), 129.0 (C), 127.9 (CH), 127.6 (CH), 127.5 (CH), 126.8 (CH), 126.4 (CH), 126.2 (CH), 125.4 (CH), 125.4 (CH), 125.2 (C), 125.2 (C), 125.0 (CH), 123.9 (CH), 74.2 (C), 45.4 (CH_2), 33.8 (CH), 32.2 (CH), 30.7 (CH_2), 29.9 ($2CH_3$), 23.6 (CH_2), 23.4 (CH_3), 2.6 ($3CH_3$); MS (ESI): 465 ($[M+Na]^+$).

Compound 19. To a solution of **18** (340 mg, 0.78 mmol) in CH_2Cl_2 (13 mL), *m*-CPBA (190 mg, 0.78 mmol) was added portionwise at 0 °C. The mixture was stirred at the same temperature for 1 h and washed with sat. aqueous $NaHCO_3$ solution (2 x 5 mL) and brine (2 x 5 mL). The organic phase was dried over Na_2SO_4 and concentrated under vacuum. Further purification by flash column chromatography (*n*-pentane/EtOAc 47:3) gave **19** (295 mg, 83%). R_f (*n*-pentane/EtOAc 47:3): 0.48; IR (neat): 3040 (w, C-H), 2963 (m, C-H), 1459 (w, C-H), 1248 (s, C-O), 1036 (s, C-O); ^{1}H NMR

(500 MHz, CD_2Cl_2): 8.30 (d, $^3J_{H\text{-}H}$ = 9.3 Hz, 1H), 8.19 (d, $^3J_{H\text{-}H}$ = 7.7 Hz, 1H), 8.18 (d, $^3J_{H\text{-}H}$ = 7.7 Hz, 1H), 8.14 (d, $^3J_{H\text{-}H}$ = 7.8 Hz, 1H), 8.13 (d, $^3J_{H\text{-}H}$ = 9.3 Hz, 1H), 8.05 (d, $^3J_{H\text{-}H}$ = 9.0 Hz, 1H), 8.04 (d, $^3J_{H\text{-}H}$ = 9.0 Hz, 1H), 8.00 (t, $^3J_{H\text{-}H}$ = 7.7 Hz, 1H), 7.90 (d, $^3J_{H\text{-}H}$ = 7.8 Hz, 1H), 3.38 – 3.34 (m, 2H), 2.71 – 2.68 (m, 1H), 1.99 – 1.80 (m, 2H), 1.79 – 1.75 (m, 1H), 1.65 – 1.42 (m, 4H), 1.25 (s, 3H), 1.20 (s, 3H), 1.20 (s, 3H), 0.08 (s, 9H); ^{13}C NMR (126 MHz, CD_2Cl_2): 135.6 (C), 130.2 (C), 129.7 (C), 128.5 (C), 127.3 (C), 126.2 (CH), 126.0 (CH), 125.9 (CH), 125.3 (CH), 124.6 (CH), 123.7 (C), 123.7 (C), 123.6 (CH), 123.5 (CH), 123.4 (CH), 122.4 (CH), 72.2 (C), 61.3 (CH), 59.2 (C), 40.0 (CH_2), 37.5 (CH_2), 32.2 (CH_2), 29.1 (CH_2), 28.1 (CH_2), 26.3 (CH_2), 23.8 ($2CH_3$), 16.4 (CH_3), 0.9 ($3CH_3$); MS (ESI): 481 ($[M+Na]^+$).

Compound 22. To a solution of **21** (320 mg, 0.73 mmol) in CH_2Cl_2 (13 mL), *m*-CPBA (145 mg, 0.59 mmol) was added portionwise at 0 °C. The mixture was stirred at the same temperature for 1 h and washed with sat. aqueous $NaHCO_3$ solution (2 x 5 mL) and brine (2 x 5 mL). The organic phase was dried over Na_2SO_4 and concentrated under vacuum. The crude residue was purified by flash column chromatography (*n*-pentane/EtOAc 47:3) to give **22** (290 mg, 87%). R_f (*n*-pentane/EtOAc 47:3): 0.48; IR (neat): 3041 (w, C-H), 2964 (m, C-H), 1460 (w, C-H), 1248 (s, C-O), 1038 (s, C-O); ^{1}H NMR (500 MHz, CD_2Cl_2): 8.31 (d, $^3J_{H\text{-}H}$ = 9.3 Hz, 1H), 8.19 (d, $^3J_{H\text{-}H}$ = 7.6 Hz, 1H), 8.17 (d, $^3J_{H\text{-}H}$ = 7.6 Hz, 1H), 8.14 (d, $^3J_{H\text{-}H}$ = 7.7 Hz, 1H), 8.13 (d, $^3J_{H\text{-}H}$ = 9.3 Hz, 1H), 8.05 (d, $^3J_{H\text{-}H}$ = 9.0 Hz, 1H), 8.04 (d, $^3J_{H\text{-}H}$ = 9.0 Hz, 1H), 8.01 (t, $^3J_{H\text{-}H}$ = 7.6 Hz, 1H), 7.91 (d, $^3J_{H\text{-}H}$ = 7.7 Hz, 1H), 3.47 – 3.31 (m, 2H), 2.67 – 2.64 (m, 1H), 2.09 – 1.91 (m, 2H), 1.79 – 1.65 (m, 2H), 1.56 – 1.42 (m, 4H), 1.26 (s, 3H), 1.13 (s, 3H), 1.12 (s, 3H), 0.10 (s, 9H); ^{13}C NMR (126 MHz, CD_2Cl_2): 137.2 (C), 131.8 (C), 131.3 (C), 130.2 (C), 129.0 (C), 127.9 (CH), 127.7 (CH), 127.6 (CH), 126.9 (CH), 126.3 (CH), 125.4 (C), 125.3 (C), 125.2 (CH), 125.1 (CH), 123.8 (CH), 73.8 (C), 65.1 (CH), 61.0 (C), 41.7 (CH_2), 34.0 (CH_2), 33.1 (CH_2), 29.9 (CH_2), 29.7 (CH_2), 28.2 (CH_2), 24.0 ($2CH_3$), 22.5 (CH_3), 2.6 ($3CH_3$); MS (ESI): 481.3 ($[M+Na]^+$).

Compound 3. To a solution of **19** (295 mg, 0.64 mmol) in dry THF (1.5 mL) at 0 °C, TBAF solution in THF (1 M, 900 μL, 0.9 mmol) was added dropwise. Afterward, the solution was stirred at

RT and the conversion was monitored by TLC (*n*-pentane/Et_2O 3:7). After 1.5 h, the mixture was diluted with Et_2O (5 mL) and washed with water (2 x 5 mL). The organic phase was dried over Na_2SO_4, filtered and concentrated under vacuum. Further purification by flash column chromatography (*n*-pentane/Et_2O 3:7 to 7:3) gave **3** (234 mg, 94%). R_f (*n*-pentane/Et_2O 3:7): 0.20; IR (neat): 3431 (w, O-H), 3040 (w, C-H), 2965 (s, C-H), 1462 (s, C-H), 1382 (s, C-O), 1152 (s, C-O), 934 (s, C-O); ^{1}H NMR (500 MHz, CD_2Cl_2): 8.30 (d, $^3J_{H-H}$ = 9.2 Hz, 1H), 8.19 (d, $^3J_{H-H}$ = 7.7 Hz, 1H), 8.18 (d, $^3J_{H-H}$ = 7.7 Hz, 1H), 8.14 (d, $^3J_{H-H}$ = 7.8 Hz, 1H), 8.13 (d, $^3J_{H-H}$ = 9.2 Hz, 1H), 8.05 (d, $^3J_{H-H}$ = 9.0 Hz, 1H), 8.04 (d, $^3J_{H-H}$ = 9.0 Hz, 1H), 8.00 (t, $^3J_{H-H}$ = 7.7 Hz, 1H), 7.90 (d, $^3J_{H-H}$ = 7.8 Hz, 1H), 3.40 – 3.30 (m, 2H), 2.72 – 2.69 (m, 1H), 2.01 – 1.89 (m, 2H), 1.82 – 1.76 (m, 1H), 1.63 – 1.48 (m, 5H), 1.27 (s, 3H), 1.18 (s, 3H), 1.17 (s, 3H); ^{13}C NMR (126 MHz, CD_2Cl_2): 136.8 (C), 131.4 (C), 130.9 (C), 129.8 (C), 128.6 (C), 127.5 (CH), 127.3 (CH), 127.2 (CH), 126.5 (CH), 125.9 (CH), 125.0 (C), 124.9 (C), 124.8 (CH), 124.8 (CH), 124.7 (CH), 123.4 (CH), 70.1 (C), 63.4 (CH), 60.8 (C), 40.1 (CH_2), 38.7 (CH_2), 33.4 (CH_2), 29.1 (CH_2), 28.9 (CH_2), 27.5 (CH_2), 23.8 ($2CH_3$), 16.4 (CH_3); HRMS (ESI): calcd. for $C_{27}H_{30}O_2$ ([M+Na]$^+$): 409.2139, found: 409.2117.

Compound 4. To a solution of **22** (290 mg, 0.64 mmol) in dry THF (1.5 mL) at 0 °C, TBAF solution in THF (1.0 M, 890 µL, 0.89 mmol) was added dropwise. Afterward, the solution was stirred at RT, and the conversion was monitored by TLC (*n*-pentane/Et_2O 3:7). After 1.5 h, the mixture was diluted with Et_2O (5 mL) and washed with water (2 x 5 mL). The organic phase was dried over Na_2SO_4, filtered and concentrated under vacuum. Further purification by flash column chromatography (*n*-pentane/Et_2O 3:7 to 7:3) gave **4** (245 mg, 99%). R_f (*n*-pentane/Et_2O 3:7): 0.20; IR (neat): 3434 (w, O-H), 3040 (w, C-H), 2966 (s, C-H), 1465 (m, C-H), 1378 (s, C-O), 952 (s, C-O); ^{1}H NMR (500 MHz, CD_2Cl_2): 8.31 (d, $^3J_{H-H}$ = 9.2 Hz, 1H), 8.19 (d, $^3J_{H-H}$ = 7.6 Hz, 1H), 8.17 (d, $^3J_{H-H}$ = 7.6 Hz, 1H), 8.14 (d, $^3J_{H-H}$ = 7.8 Hz, 1H), 8.13 (d, $^3J_{H-H}$ = 9.3 Hz, 1H), 8.04 (d, $^3J_{H-H}$ = 9.0 Hz, 1H), 8.03 (d, $^3J_{H-H}$ = 9.0 Hz, 1H), 8.00 (t, $^3J_{H-H}$ = 7.6 Hz, 1H), 7.91 (d, $^3J_{H-H}$ = 7.8 Hz, 1H), 3.48 – 3.42 (m, 1H), 3.37 – 3.31 (m, 1H), 2.07 – 1.92 (m, 2H), 1.79 – 1.73 (m, 1H), 1.66 – 1. 85 (m, 1H), 1.48 – 1.36 (m, 4H), 1.27 (s, 3H), 1.05 (s, 3H), 1.04 (s, 3H); ^{13}C NMR (126 MHz, CD_2Cl_2): 137.1

(C), 131.8 (C), 131.3 (C), 130.2 (C), 129.0 (C), 127.9 (CH), 127.8 (CH), 127.6 (CH), 126.9 (CH), 126.3 (CH), 125.4 (C), 125.3 (C), 125.2 (CH), 125.2 (CH), 125.1 (CH), 123.8 (CH), 70.4 (C), 65.1 (CH), 61.5 (C), 40.7 (CH_2), 33.9 (CH_2), 33.1 (CH_2), 29.4 (CH_2), 29.2 (CH_2), 28.2 (CH_2), 23.9 (CH_3), 22.5 ($2CH_3$); HRMS (ESI): calcd. for $C_{27}H_{30}O_2$ ($[M+Na]^+$): 409.2139, found: 409.2117.

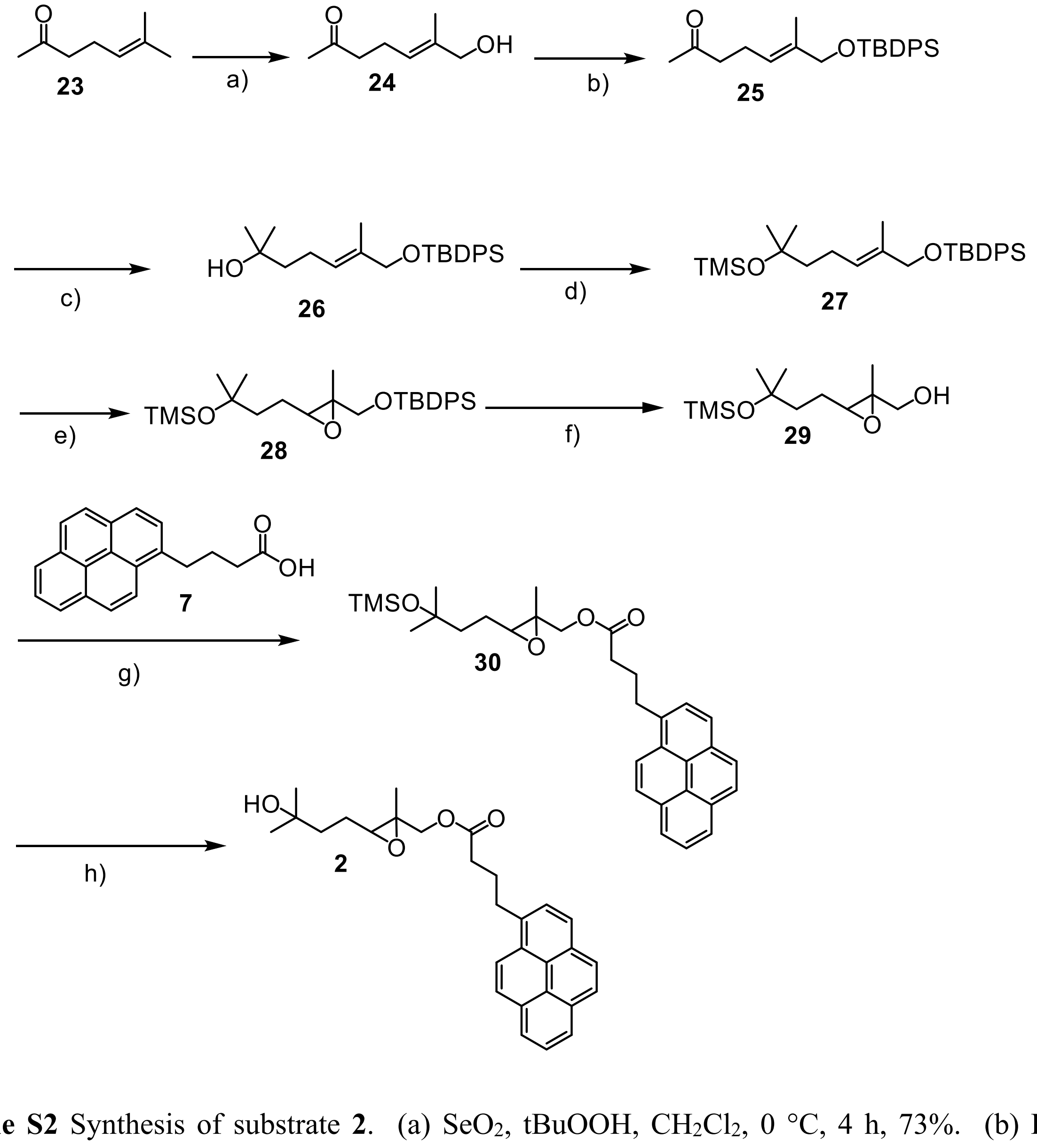

Scheme S2 Synthesis of substrate **2**. (a) SeO_2, tBuOOH, CH_2Cl_2, 0 °C, 4 h, 73%. (b) DMAP, Imidazole, TBDPSCl, RT, 3 h, 65%. (c) MeMgBr, dry THF, 0°C to RT, 2 h, 84%. (d) DMAP, Et_3N, TMSCl, CH_2Cl_2, RT, 2 h, 72%. (e) *m*-CPBA, CH_2Cl_2, 0 °C to RT, 15 h, 57%. (f) TBAF, THF, 0°C to RT, 2 h, 63%. (g) **7**, DMAP, DCC, CH_2Cl_2, RT, 12 h, 97%. (h) TBAF, THF, 0 °C to RT, 2 H, 67%.

Compound 24. was prepared following the reported procedures.[S5]

Compound 25. To a solution of **24** (3.0 g, 21 mmol) in CH_2Cl_2 (29 mL), DMAP (33 mg, 0.27 mmol) and Imidazole (2.2 g, 32 mmol) were added at 0 °C. After 10 minutes, TBDPSCl (8.8 g, 32 mmol) was added to the mixture at the same temperature. The solution was warmed up to RT and stirred for 3 h. The mixture was filtered to remove the solid and then purified by flash column chromatography (*n*-pentane/CH_2Cl_2 4:1 to pure CH_2Cl_2) to obtain **25** (5.21 g, 65%) as a colorless oil. R_f (*n*-pentane/CH_2Cl_2 4:1): 0.4; IR (neat): 3070 (w, C-H), 2930 (w, C-H), 1716 (s, C=O), 1427 (w, C-H), 1360 (w, C-O-C), 1108 (s, C-O); ^{1}H NMR (500 MHz, $CDCl_3$): 7.68 – 7.66 (m, 4H), 7.44 – 7.36 (m, 6H), 5.39 (t, $^3J_{H-H}$ = 8.7 Hz, 1H), 4.03 (s, 2H), 2.46 (t, $^3J_{H-H}$ = 7.5 Hz, 2H), 2.33 – 2.31 (m, 2H), 2.14 (s, 3H), 1.61 (s, 3H), 1.06 (s, 9H); ^{13}C NMR (126 MHz, $CDCl_3$): 209.3 (C=O), 136.2 (4CH), 135.6 (C), 134.2 (2C), 129.8 (2CH), 127.7 (4CH), 122.8 (CH), 69.1 (CH_2), 43.1 (CH_2), 30.7 (CH_3), 25.9 (3CH_3), 22.2 (CH_2), 19.7 (C), 13.9 (CH_3); MS (ESI): 403 ([M+Na]$^+$).

Compound 26. To a solution of **25** (5.2 g, 14 mmol) in dry THF (70 mL), MeMgBr solution in Et_2O (3.0 M, 7.2 mL, 21 mmol) was added at 0 °C. After stirring at RT for 2 h, the reaction mixture was quenched with sat. aqueous NH_4Cl (40 mL) and extracted with CH_2Cl_2 (3 x 40 mL). The combined organic phases were washed with brine (2 x 40 mL), dried over Na_2SO_4 and concentrated in vacuo. The residue was purified by flash column chromatography (pure CH_2Cl_2) to give **26** (4.6 g, 84%) as a colorless oil. R_f (CH_2Cl_2): 0.23; IR (neat): 3373 (w, O-H), 3071 (w, C-H), 2962 (w, C-H), 1427 (w, C-H), 1109 (s, C-O), 1058 (s, C-O); ^{1}H NMR (500 MHz, $CDCl_3$): 7.69 – 7.67 (m, 4H), 7.43 – 7.36 (m, 6H), 5.46 – 5.43 (m, 1H), 4.05 (s, 2H), 2.14 – 2.09 (m, 2H), 1.62 (s, 3H), 1.54 – 1.50 (m, 2H), 1.24 (s, 6H), 1.06 (s, 9H); ^{13}C NMR (126 MHz, $CDCl_3$): 135.9 (4CH), 134.3 (C), 134.0 (2C), 129.9 (2CH), 127.9 (4CH), 124.4 (CH), 72.0 (C), 69.3 (CH_2), 43.6 (CH_2), 29.4 (2CH_3), 27.0 (3CH_3), 22.8 (CH_2), 19.5 (C), 13.6 (CH_3); MS (ESI): 419.3 ([M+Na]$^+$).

Compound 27. To a solution of **26** (4.58 g, 11.5 mmol) and DMAP (142 mg, 1.15 mmol) in CH_2Cl_2 (53 mL) under an inert atmosphere, Et_3N (4.9 mL, 35 mmol) was added, followed by dropwise addition of TMSCl (3.0 mL, 23 mmol). The mixture was stirred at RT for 2 h. The reaction

mixture was diluted with CH_2Cl_2 (10 mL) and quenched by the addition of H_2O. The organic phase was washed with H_2O (2 x 25 mL) and HCl aqueous solution (1 M, 2 x 25 mL). The organic phase was dried over Na_2SO_4, filtered and concentrated under reduced pressure to yield **27** (3.9 g, 72%) as a colorless oil. R_f (*n*-pentane/EtOAc 4:1): 0.4; IR (neat): 3071 (w, C-H), 2960 (w, C-H), 2857 (m, C-H), 1428 (w, C-H), 1110 (s, C-O), 1044 (s, C-O); ^{1}H NMR (500 MHz, $CDCl_3$): 7.69 – 7.68 (m, 4H), 7.42 – 7.36 (m, 6H), 5.42 – 5.41 (m, 1H), 4.05 (s, 2H), 2.11 – 2.06 (m, 2H), 1.62 (s, 3H), 1.48 – 1.44 (m, 2H), 1.23 (s, 6H), 1.06 (s, 9H), 0.12 (s, 9H); ^{13}C NMR (126 MHz, $CDCl_3$): 135.9 (4CH), 134.4 (2C), 133.8 (C), 129.7 (2CH), 127.8 (4CH), 125.7 (CH), 74.0 (C), 69.4 (CH_2), 44.6 (CH_2), 30.0 ($2CH_3$), 27.1 ($3CH_3$), 22.7 (CH_2), 19.5 (C), 13.0 (CH_3), 2.76 ($3CH_3$); MS (ESI): 491.3 ($[M+Na]^+$).

Compound 28. To a solution of **27** (3.9 g, 8.3 mmol) in CH_2Cl_2 (100 mL), *m*-CPBA (2.0 g, 8.3 mmol) was added portionwise at 0 °C. Afterward, the mixture was warmed to RT and stirred for 15 h. The mixture was concentrated under vacuum and purified by flash column chromatography (*n*-pentane to *n*-pentane/EtOAc 4:1) to obtain **28** (2.3 g, 57%) as a colorless oil. R_f (*n*-pentane/EtOAc 4:1): 0.35; IR (neat): 3071 (w, C-H), 2960 (w, C-H), 2857 (m, C-H), 1427 (w, C-H), 1249 (m, C-O), 1111 (s, C-O), 1039 (s, C-O); ^{1}H NMR (500 MHz, $CDCl_3$): 7.68 – 7.66 (m, 4H), 7.44 – 7.36 (m, 6H), 3.65 (d, $^2J_{H\text{-}H}$ = 11 Hz, 1H), 3.58 (d, $^2J_{H\text{-}H}$ = 11 Hz, 1H), 2.78 (t, $^3J_{H\text{-}H}$ = 6.2 Hz, 1H), 1.68 – 1.22 (m, 4H), 1.33 (s, 3H), 1.23 (s, 3H), 1.22 (s, 3H), 1.06 (s, 9H), 0.10 (s, 9H); ^{13}C NMR (126 MHz, $CDCl_3$): 137.1 (4CH), 133.4 (2C), 129.7 (2CH), 127.8 (4CH), 73.4 (C), 68.6 (CH_2), 61,4 (CH), 60.9 (C), 41.1 (CH_2), 30.0 (CH_3), 29.6 (CH_3), 26.8 ($3CH_3$), 23.4 (CH_2), 19.3 (C), 14.2 (CH_3), 2.81 ($3CH_3$); MS (ESI): 507.3 ($[M+Na]^+$).

Compound 29. To a solution of **28** (1.2 g, 2.4 mmol) in dry THF (4.9 mL) at 0 °C, TBAF solution in THF (1.0 M, 1.4 mL, 1.5 mmol) was added dropwise. The mixture was stirred at the same temperature for 1 h before the addition of more TBAF (0.4 mL, 0.5 mmol) and stirred for 30 min at 0 °C. Afterward, the mixture was diluted with Et_2O (10 mL) and washed with water (2 x 10 mL). The organic phase was dried over Na_2SO_4, filtered and concentrated under vacuum. Further purification by flash column chromatography (*n*-pentane/Et_2O 3:7) gave **29** (370 mg, 63%) as a colorless oil. R_f

(n-pentane/Et_2O 3:7): 0.50; IR (neat): 3436 (w, O-H), 2968 (w, C-H), 1459 (w, C-H), 1248 (m, C-O), 1036 (s, C-O); ^{1}H NMR (500 MHz, $CDCl_3$): 3.68 (dd, $^2J_{H-H}$ = 12.1 Hz, $^3J_{H-H}$ = 4.4 Hz, 1H), 3.58 (dd, $^2J_{H-H}$ = 12.1 Hz, $^3J_{H-H}$ = 8.4 Hz, 1H), 3.05 – 3.05 (m, 1H), 1.72 – 1.64 (m, 2H), 1.64- 1.56 (m, 2H), 1.53 – 1.47 (m, 1H), 1.29 (s, 3H), 1.24 (s, 3H), 1.23 (s, 3H), 0.10 (s, 9H); ^{13}C NMR (126 MHz, $CDCl_3$): 73.5 (C), 65.6 (CH_2), 61,1 (C), 60.5 (CH), 41.2 (CH_2), 30.1 (CH_3), 29.7 (CH_3), 23.4 (CH_2), 14.3 (CH_3), 2.7 ($3CH_3$); MS (ESI): 269 ($[M+Na]^+$).

Compound 30. To a solution of **29** (20 mg, 0.08 mmol) in CH_2Cl_2 (380 µL) at RT was added **7** (36 mg, 0.12 mmol) followed by DCC (30 mg, 0.15 mmol) and DMAP (15 mg, 0.12 mmol). After stirring at RT for 12 h, the reaction mixture was diluted with CH_2Cl_2 (5 mL) and filtered. The filtrate was washed with sat. aqueous $NaHCO_3$ (5 mL). The aqueous phase was extracted with CH_2Cl_2 (3 x 10 mL). The combined organic phases were dried over Na_2SO_4 and concentrated. Further purification by flash chromatography (n-pentane/Et_2O 1:1) gave **30** (41 mg, 97%) as a colorless oil. R_f (n-pentane/Et_2O 1:1): 0.55; IR (neat): 3041 (m, C-H), 2966 (m, C-H), 1736 (s, C=O), 1458 (w, C-H), 1248 (m, C-O), 1036 (s, C-O); ^{1}H NMR (500 MHz, $CDCl_3$): 8.31 (d, $^3J_{H-H}$ = 9.1 Hz, 1H), 8.18 (d, $^3J_{H-H}$ = 7.7 Hz, 1H), 8.16 (d, $^3J_{H-H}$ = 7.7 Hz, 1H), 8.13 (d, $^3J_{H-H}$ = 9.1 Hz, 1H), 8.12 (d, $^3J_{H-H}$ = 7.5 Hz, 1H), 8.04 (d, $^3J_{H-H}$ = 10.2 Hz, 1H), 8.02 (d, $^3J_{H-H}$ = 10.2 Hz, 1H), 7.99 (t, $^3J_{H-H}$ = 7.7 Hz, 2H), 7.87 (d, $^3J_{H-H}$ = 7.5 Hz, 1H), 4.21 (d, $^2J_{H-H}$ = 11.8 Hz, 1H), 3.97 (d, $^2J_{H-H}$ = 11.8 Hz, 1H), 3.41 (dd, $^3J_{H-H}$ = 7.5, 6.2 Hz, 2H), 2.90 – 2.88 (m, 1H), 2.51 (t, $^3J_{H-H}$ = 7.5 Hz, 1H), 2.25 – 2.19 (m, 2H), 1.70 – 1.46 (m, 4H), 1.32 (s, 3H), 1.22 (s, 3H), 1.21 (s, 3H), 0.09 (s, 9H); ^{13}C NMR (126 MHz, $CDCl_3$): 172.2 (C=O), 135.8 (C), 131.6 (C), 131.1 (C), 130.2 (C), 128.9 (C), 127.6 (CH), 127.6 (CH), 127.5 (CH), 126.9 (CH), 126.0 (CH), 125.3 (C), 125.1 (C), 125.1 (CH), 125.0 (CH), 125.0 (CH), 123.5 (CH), 73.4 (C), 68.6 (CH_2), 61.7 (CH), 58.7 (C), 41.1 (CH_2), 33.8 (CH_2), 32.9 (CH_2), 30.1 (CH_3), 29.7 (CH_3), 26.9 (CH_2), 23.4 (CH_2), 14.5 (CH_3), 2.69 ($3CH_3$); MS (ESI): 539 ($[M+Na]^+$).

Compound 2. Compound **30** (615 mg, 1.2 mmol) was dissolved in dry THF (2.4 mL), and a solution of TBAF in THF (1.0 M, 715 µL, 715 µmol) was added dropwise at 0 °C. Afterward, the mixture was stirred for 2 h at RT. Then, the solvent was evaporated under vacuum, and the crude

mixture was purified by flash column chromatography (*n*-pentane/Et_2O 1:0 to 0:1) to afford **2** (350 mg, 67%) as a white solid. R_f (pure Et_2O): 0.30; Mp: 84 – 85 °C; IR (neat): 3516 (w, O-H), 2968 (w, C-H), 1727 (s, C=O), 1382 (m, O-H), 1171 (s, C-O), 1159 (s, C-O); ^{1}H NMR (500 MHz, CD_2Cl_2): 8.33 (d, $^3J_{H-H}$ = 9.2 Hz, 1H), 8.19 – 8.16 (m, 2H), 8.14 – 8.12 (m, 2H), 8.04 (d, $^2J_{H-H}$ = 10.7 Hz, 1H), 8.03 (d, $^2J_{H-H}$ = 10.7 Hz, 1H), 8.00 (t, $^3J_{H-H}$ = 7.6 Hz, 1H), 7.89 (d, $^3J_{H-H}$ = 7.8 Hz, 1H), 4.19 (d, $^2J_{H-H}$ = 11.8 Hz, 1H), 3.97 (d, $^2J_{H-H}$ = 11.8 Hz, 1H), 3.40 (dd, $^3J_{H-H}$ = 7.5, 6.1 Hz, 2H), 2.87 – 2.85 (m, 1H), 2.51 (t, $^3J_{H-H}$ = 7.5 Hz, 2H), 2.21 – 2.15 (m, 2H), 1.64 – 1.51 (m, 5H), 1.30 (s, 3H), 1.17 (s, 6H); ^{13}C NMR (126 MHz, $CDCl_3$): 173.3 (C=O), 136.4 (C), 132.4 (C), 131.3 (C), 130.4 (C), 129.6 (C), 127.9 (CH), 127.8 (CH), 127.7 (CH), 127.0 (CH), 126.3 (CH), 125.4 (C), 125.3 (C), 125.2 (CH), 125.1 (CH), 123.8 (CH), 70.5 (C), 68.7 (CH_2), 61.7 (CH), 59.0 (C), 40.5 (CH_2), 34.0 (CH_2), 33.1 (CH_2), 29.5 (CH_3), 29.4 (CH_3), 27.2 (CH_2), 23.7 (CH_2), 14.5 (CH_3); HRMS (ESI): calcd. for $C_{29}H_{32}O_4$ ($[M+Na]^+$): 467.2193, found: 467.2211.

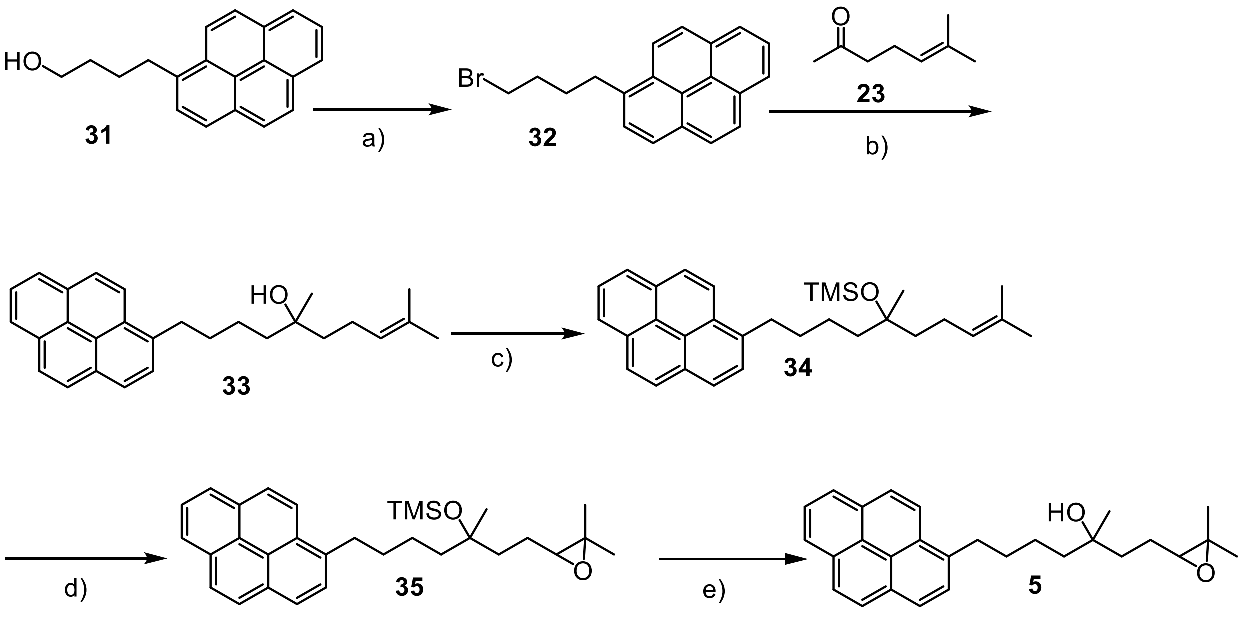

Scheme S3 (a) PPh_3, CBr_4, CH_2Cl_2, RT, 45 min, 90%. (b) Mg, 1,2-Dibromoethane, THF, RT, 30 min, **23**, RT, 15 h, 41%. (c) Et_3N, DMAP, TMSCl, CH_2Cl_2, RT, 2 h, 76%. (d) *m*-CPBA, CH_2Cl_2, 0°C, 30 min, RT, 4h 70%. (e) TBAF, THF, RT, 2 h, 86%.

Compound 32. was synthesized according to the procedure described in the reference.[S6]

Compound 33. To an oven-dried Schlenk flask under Ar atmosphere, Mg metal was added (232 mg, 9.55 mmol), followed by dry THF (5 mL) and 1,2-dibromoethane (1 drop). A solution of **32** (1.52 g, 4.51 mmol) in dry THF (3 mL) was prepared, and a third (1 mL) was added to the Schlenk flask. The mixture was stirred at RT until the flask became warm to the touch. Then, the remaining bromide solution was added dropwise. The solution was stirred for 45 min at RT and monitored by TLC for the consumption of **32**. Another temperature increase (~ 40 °C) followed the remaining Grignard product formation, and the solution turned brownish red. The mixture was then cooled to 0 °C, and compound **23** (1.45 mL, 9.65 mmol) was added. The solution was stirred at RT for 15 h. The mixture was diluted in EtOAc and washed with saturated NH_4Cl (x3) and brine (x1), dried over Na_2SO_4, filtered, and concentrated. The crude mixture was purified by flash column chromatography (40 g silica, linear gradient 0-15% EtOAc in *n*-pentane) to yield **33** as a light brownish-red wax (711 mg, 41%). R_f (*n*-pentane/EtOAc 7:1): 0.56; IR (neat): 3422 (b, O-H), 3040 (w, C-H), 2930 (s, C-H), 2858 (m, C-H), 1587 (w, C-C), 1603 (w, C=C),1457 (m, C-C), 1375 (m, C-H), 1182 (m, C-O), 1113 (m, C-H), 916 (w, C-H); ^{1}H NMR (400 MHz, $CDCl_3$): 8.28 (d, $^3J_{H-H}$ = 9.3 Hz, 1H), 8.18 – 8.14 (m, 2H), 8.11 (d, $^3J_{H-H}$ = 7.7 Hz, 1H), 8.10 (d, $^3J_{H-H}$ = 9.30 Hz, 1H), 8.06 – 7.95 (m, 3H), 7.87 (d, $^3J_{H-H}$ = 7.8 Hz, 1H), 5.13 (m, 1H), 3.41 – 3.32 (m, 2H), 2.09 – 1.98 (m, 2H), 1.93 – 1.78 (m, 2H), 1.68 (s, 3H), 1.60 (s, 3H), 1.58 – 1.46 (m, 6H), 1.19 (s, 3H); ^{13}C NMR (101 MHz, $CDCl_3$): 137.2 (C), 131.9 (C), 131.6 (C), 131.1 (C), 129.9 (C), 128.7 (C), 127.7 (CH), 127.4 (CH), 127.3 (CH), 126.7 (CH), 125.9 (CH), 125.3 (C), 125.2 (C), 125.0 (2xCH), 124.8 (CH), 124.6 (CH), 123.6 (CH), 73.0 (C), 42.0 (CH_2), 41.8 (CH_2), 34.5 (CH_2), 32.7 (CH_2), 27.0 (CH_3), 25.9 (CH_3), 24.3 (CH_2), 22.8 (CH_2), 17.8 (CH_3).

Compound 34. To a solution of **33** (711 mg, 1.85 mmol) in CH_2Cl_2 (10 mL), DMAP (27.1 mg, 222 µmol) and Et_3N (773 µL, 5.55 mmol) were added sequentially at 0 °C. Then, under nitrogen atmosphere, TMSCl (469 µL, 3.70 mmol) was added dropwise at the same temperature. The resulting yellow mixture was stirred at RT for 2 h. Afterward, the crude mixture was washed with 10% citric

acid solution (x3) and brine (x2). The organic phase was dried over Na_2SO_4 and concentrated. The crude residue was finally purified by flash chromatography (25g silica, linear gradient 0-15% CH_2Cl_2 in *n*-pentane) to yield **34** a colourless oil (641 mg, 76%). R_f (Pentane/CH_2Cl_2 7:1): 0.83; IR (neat): 3040 (w, C-H), 2936 (s, C-H), 2860 (m, C-H), 1604 (w, C=C), 1454.3 (m, C-C), 1374 (m, C-H), 1248 (s, C-O), 1182 (w, C-H), 1115 (w, C-H), 1092 (m, C-H), 1043 (s, O-Si), 867 (s, C-H); ^1H NMR (400 MHz, $CDCl_3$): 8.29 (d, $^3J_{H-H}$ = 9.3 Hz, 1H), 8.18 – 8.14 (m, 2H), 8.11 (d, $^3J_{H-H}$ = 7.8 Hz, 1H), 8.10 (d, $^3J_{H-H}$ = 9.3 Hz, 1H), 8.06 – 7.95 (m, 3H), 7.88 (d, $^3J_{H-H}$ = 7.8 Hz, 1H), 5.11 (m, 1H), 3.40 – 3.31 (m, 2H), 1.99 (q, $^3J_{H-H}$ = 7.9 Hz, 2H), 1.91 – 1.78 (m, 2H), 1.68 (s, 3H), 1.59 (s, 3H), 1.58 – 1.38 (m, 6H), 1.20 (s, 3H), 0.10 (s, 9H); ^{13}C NMR (126 MHz, $CDCl_3$): 137.4 (C), 131.6 (C), 131.2 (C), 131.1 (C), 129.9 (C), 128.7 (C), 127.7 (CH), 127.4 (CH), 127.3 (CH), 126.6 (CH), 125.9 (CH), 125.2 (C), 125.2 (C), 125.0 (CH), 124.9 (CH), 124.9 (CH), 124.8 (CH), 123.6 (CH), 76.2 (C), 42.5 (2CH_2), 33.9 (CH_2), 32.7 (CH_2), 27.6 (CH_3), 25.9 (CH_3), 24.6 (CH_2), 23.0 (CH_2), 17.7 (CH_3), 2.8 (3CH_3).

Compound 35. To a solution of **34** (641 mg, 1.40 mmol) in dry CH_2Cl_2 (25 mL), *m*-CPBA (70-75% purity, 346 mg, 1.40 mmol) was added portionwise at 0 °C. Afterward, the solution was left to stir at RT for 2 h (monitored by TLC). The mixture was finally quenched and washed with saturated $NaHCO_3$ (x3) and brine (x1). The organic phase was dried over Na_2SO_4 and concentrated. The crude product was purified by flash chromatography (25 g silica, 50% CH_2Cl_2 in *n*-pentane) to yield **35** as a transparent oil (464 mg, 70%). R_f (*n*-pentane/CH_2Cl_2 1:1): 0.43; IR (neat): 3039 (w, C-H), 2937 (s, C-H), 2860 (m, C-H), 1604 (w, C-C), 1588 (w, C-C), 1491 (w, C-H), 1458 (m, C-H), 1376 (m, C-H), 1321 (w, C-H), 1249 (s, C-O), 1183 (w, C-H), 1138 (w, C-H), 1119 (m, C-O-C), 1044 (s, O-Si), 997 (w, C-H); R_f (*n*-pentane/CH_2Cl_2 1:1): 0.43; ^1H NMR (500 MHz, $CDCl_3$; 0.55/0.46 diastereoisomeric peaks): 8.31 (d, $^3J_{H-H}$ = 9.2 Hz, 1H), 8.21 – 8.11 (m, 2H), 8.11 (d, $^3J_{H-H}$ = 7.7 Hz, 1H), 8.10 (d, $^3J_{H-H}$ = 9.2 Hz, 1H), 8.09 – 7.97 (m, 3H), 7.90 (d, $^3J_{H-H}$ = 7.8 Hz, 1H), 3.41 – 3.30 (m, 2H), 2.68 – 2.59 (m, 1H), 1.90 – 1.80 (m, 2H), 1.69 – 1.41 (m, 8H), 1.26 (s, 3H), 1.21/1.20 (s, 3H), 0.09/0.09 (s, 9H); ^{13}C NMR (126 MHz, $CDCl_3$; *nn*/*nn* diastereomeric peaks): 137.8 (C), 131.8 (C), 131.4 (C), 130.1 (C), 129.0 (C), 127.9 (CH), 127.7 (CH), 127.4 (CH), 126.8 (CH), 126.2 (CH), 125.4 (C), 125.4 (C), 125.2

(CH), 125.1 (CH), 125.0 (CH), 123.9 (CH), 76.1 (C), 64.8 (CH), 58.4 (C), 42.8/42.4 (CH_2), 38.8/38.7 (CH_2), 34.0 (CH_2), 32.9 (CH_2), 27.6/27.4 (CH_3), 25.1 (CH_3), 24.6/24.6 (CH_2), 24.0/23.9 (CH_2), 18.8 (CH_3), 2.8 ($3CH_3$).

Compound 5. To a solution of **35** (464 mg, 982 µmol) in dry THF (20.0 mL), TBAF solution (1.0 M in THF, 982 µL, 980 µmol) was added dropwise at 0 °C. The solution was stirred for 2 h at RT (TLC monitoring). Finally, the solvent was evaporated *in vacuo*, and the crude product was directly purified by flash column chromatography (4 g silica, linear gradient 0-20% EtOAc in *n*-pentane) to yield **5** (338 mg 86%) as a yellow oil. R_f (*n*-pentane/EtOAc 7:1): 0.15; IR (neat): 3446 (b, O-H), 3040 (w, C-H), 2932 (s, C-H), 2861 (m, C-H), 1587 (w, C-C), 1603 (w, C-C), 1488 (w, C-H), 1460 (s, C-H), 1417 (w, C-H), 1322 (w, C-H), 1248 (w, C-H), 1182 (s, C-O), 1120 (s, C-O-C), 1009 (w, C-H), 918 (w, C-H); ^{1}H NMR (400 MHz, CD_2Cl_2; 0.55/0.46 diastereoisomeric peaks): 8.31 (d, $^3J_{H-H}$ = 9.2 Hz, 1H), 8.20 – 8.16 (m, 2H), 8.14 (d, $^3J_{H-H}$ = 3.1 Hz, 1H), 8.12 (d, $^3J_{H-H}$ = 4.6 Hz, 1H), 8.05 – 7.98 (m, 3H), 7.90 (d, $^3J_{H-H}$ = 7.8 Hz, 1H), 3.41 – 3.31 (m, 2H), 2.70 – 2.65 (m, 1H), 1.94 – 1.76 (m, 2H), 1.62 – 1.48 (m, 8H), 1.39-1.35 (2s, 1H), 1.26 (s, 3H), 1.22 (s, 3H), 1.16 (s, 3H); ^{13}C NMR (126 MHz, CD_2Cl_2; *nn*/*nn* diastereomeric peaks): 137.7 (C), 131.8 (C), 131.3 (C), 130.1 (C), 129.0 (C), 127.9 (CH), 127.7 (CH), 127.4 (CH), 126.8 (CH), 126.2 (CH), 125.4 (C), 125.3 (C), 125.2 (CH), 125.2 (CH), 125.0 (CH), 123.9 (CH), 72.4 (C), 64.8 (CH), 58.8 (C), 42.4/42.2 (CH_2), 38.7/38.6 (CH_2), 33.9 (CH_2), 32.9 (CH_2), 27.1/27.0 (CH_3), 25.0 (CH_3), 24.6/24.5 (CH_2), 24.0/24.0 (CH_2), 18.8 (CH_3); HRMS (ESI, +ve) calcld for $C_{28}H_{32}O_2Na$: 423.2295, found: 423.2284.

2.2. Synthesis of product references

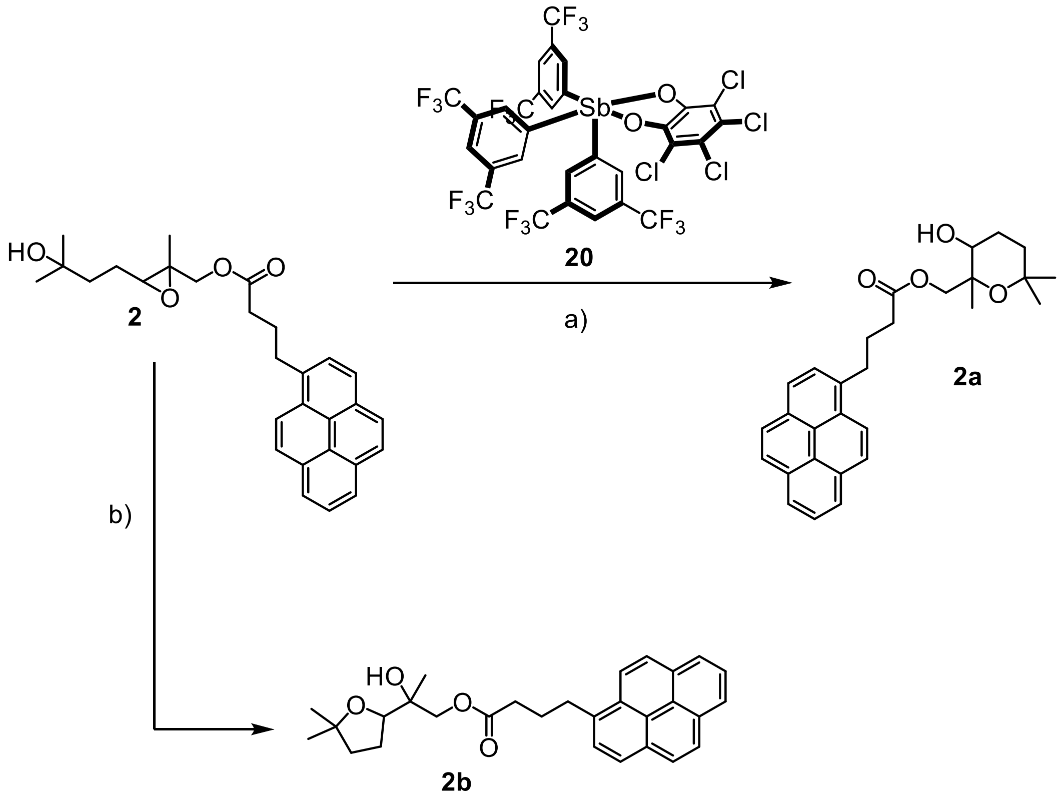

Scheme S4 (a) **20**, CH_2Cl_2, RT, 30 min, 17%; (b) $SbCl_3$, CH_2Cl_2, RT, 2 h, 50%.

Compound 2a. To a solution of **2** (150 mg, 340 µmol) in CH_2Cl_2 (2 mL), **20** (65 mg, 250 µmol) was added. The mixture was stirred for 30 min at RT. Then, the reaction mixture was diluted with CH_2Cl_2 (1 mL) and washed with water (2 x 1 mL) and brine (2 x 1 ml). The organic phase was dried over Na_2SO_4 and concentrated under vacuum. The crude mixture was purified by preparative TLC (*n*-pentane/EtOAc 3:1, developed twice) to give **2a** (25 mg, 17%) as a colorless oil. R_f (*n*-pentane/EtOAc 3:1, twice): 0.3; IR (neat): 3504 (m, O-H), 2966 (m, C-H), 2944 (m, C-H), 1710 (s, C=O), 1185 (s, C-O), 1030 (s, C-O-C), 996 (s, C-O-C); ^{1}H NMR (500 MHz, CD_2Cl_2): 8.33 (d, $^3J_{H-H}$ = 9.2 Hz, 1H), 8.18 (d, $^3J_{H-H}$ = 7.5 Hz, 1H), 8.17 (d, $^3J_{H-H}$ = 7.5 Hz, 1H), 8.13 (d, $^3J_{H-H}$ = 8.6 Hz, 2H), 8.50 (d, $^3J_{H-H}$ = 9.0 Hz, 1H), 8.40 (d, $^3J_{H-H}$ = 9.0 Hz, 1H), 8.00 (t, $^3J_{H-H}$ = 7.5 Hz, 1H), 7.90 (d, $^3J_{H-H}$ = 7.8 Hz, 1H), 4.10 (d, $^2J_{H-H}$ = 11.3 Hz, 1H), 3.91 (d, $^2J_{H-H}$ = 11.3 Hz, 1H), 3.49 (dd, $^3J_{H-H}$ = 10.8, 4.8 Hz, 1H), 3.42 – 3.39 (m, 2H), 2.51 (t, $^3J_{H-H}$ = 7.4 Hz, 2H), 2.33 – 2.16 (m, 2H), 1.77 – 1.66 (m,

4H), 1.22 (s, 3H), 1.18 (s, 3H), 1.10 (s, 3H); ^{13}C NMR (126 MHz, CD_2Cl_2): 174.2 (C=O), 136.4 (C), 131.8 (C), 131.3 (C), 130.4 (C), 129.1 (C), 127.9 (CH), 127.7 (CH), 127.0 (CH), 126.3 (CH), 125.4 (2C), 125.3 (CH), 125.2 (CH), 125.2 (CH), 123.8 (CH), 76.3 (C), 72.1 (C), 70.2 (CH_2), 69.6 (CH), 36.5 (CH_2), 34.2 (CH_2), 33.1 (CH_2), 32.5 (CH_3), 27.8 (CH_3), 27.3 (CH_2), 24.8 (CH_2), 18.3 (CH_3).

Compound 2b. To a solution of **2** (39 mg, 88 μmol) in CH_2Cl_2 (0.5 mL), $SbCl_3$ (2 mg, 9 μmol) was added. Then, the mixture was stirred for 2 h at RT. The reaction mixture was diluted with CH_2Cl_2 (1 mL) and washed with sat. aqueous NaOH (2 x 1 mL) and water (1 x 1 ml). The organic phase was dried over Na_2SO_4 and concentrated under vacuum to give **2b** (19 mg, 50%) as a colorless oil. R_f (*n*-pentane/EtOAc 3:1): 0.5; IR (neat): 3504 (m, O-H), 2966 (m, C-H), 2943 (m, C-H), 1710 (s, C=O), 1185 (s, C-O), 1153 (s, C-O-C), 996 (s, C-O-C); ^{1}H NMR (500 MHz, CD_2Cl_2): 8.33 (d, $^3J_{H-H}$ = 9.1 Hz, 1H), 8.18 (d, $^3J_{H-H}$ = 7.8 Hz, 1H), 8.17 (d, $^3J_{H-H}$ = 7.8 Hz, 1H), 8.13 (d, $^3J_{H-H}$ = 7.3 Hz, 1H), 8.12 (d, $^3J_{H-H}$ = 9.1 Hz, 1H), 8.07 – 8.03 (m, 2H), 8.00 (t, $^3J_{H-H}$ = 7.8 Hz, 1H), 7.91 (d, $^3J_{H-H}$ = 7.3 Hz, 1H), 4.07 – 4.01 (m, 2H), 3.88 (t, $^3J_{H-H}$ = 7.4 Hz, 1H), 3.42 – 3.39 (m, 2H), 2.51 (t, $^3J_{H-H}$ = 7.3 Hz, 2H), 2.22 – 2.16 (m, 2H), 1.92 – 1.85 (m, 2H), 1.75 – 1.66 (m, 2H), 1.22 (s, 3H), 1.20 (s, 3H), 1.18 (s, 3H); ^{13}C NMR (126 MHz, CD_2Cl_2): 173.7 (C=O), 136.4 (C), 131.8 (C), 131.3 (C), 130.3 (C), 129.1 (C), 127.9 (CH), 127.8 (CH), 127.7 (CH), 127.0 (CH), 126.3 (CH), 125.4 (C), 125.3 (C), 125.2 (CH), 125.2 (CH), 125.1 (CH), 123.8 (CH), 82.4 (CH), 81.4 (C), 72.5 (C), 68.9 (CH_2), 38.9 (CH_2), 34.2 (CH_2), 33.1 (CH_2), 28.7 (CH_3), 27.9 (CH_3), 27.2 (CH_2), 26.6 (CH_2), 21.6 (CH_3).

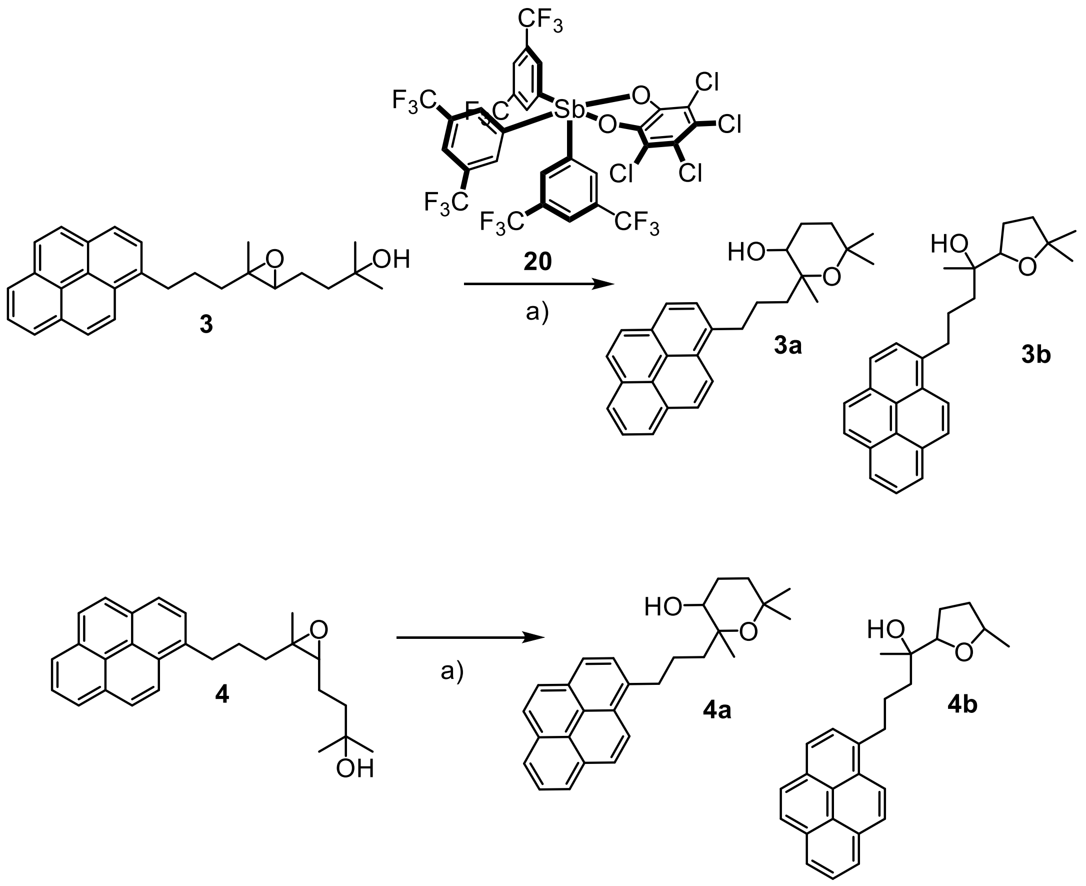

Scheme S5 (a) **20**, CH_2Cl_2, RT, 30 min, 81% **3a**, 14% **3b**, 82% **4a**, 8% **4b**.

Compounds 3a and 3b. To a solution of **3** (23 mg, 60 μmol) in CH_2Cl_2 (320 μL), **20** (5 mg, 6 μmol) was added. Afterward, the mixture was stirred for 30 min at RT. Then, the reaction mixture was diluted with CH_2Cl_2 (0.5 mL) and washed with water (2 x 0.5 mL) and brine (2 x 0.2 ml). The organic phase was dried over Na_2SO_4 and concentrated under vacuum. The crude mixture was purified by preparative TLC (*n*-pentane/EtOAc 9:1, developed twice) to give compound **3b** (R_f 0.58, 3 mg, 14%) and compound **3a** (R_f 0.28, 19 mg, 81%) as colorless oils.

Compound 3b. IR (neat): 3443 (w, O-H), 2968 (m, C-H), 2932 (m, C-H), 1457 (m, O-H), 1370 (m, C-H), 1043 (s, C-O), 1003 (s, C-O-C); ^{1}H NMR (500 MHz, CD_2Cl_2): 8.32 (d, $^3J_{H-H}$ = 9.2 Hz, 1H), 8.19 (d, $^3J_{H-H}$ = 7.6 Hz, 1H), 8.17 (d, $^3J_{H-H}$ = 7.6 Hz, 1H), 8.14 (d, $^3J_{H-H}$ = 7.8 Hz, 1H), 8.13 (d, $^3J_{H-H}$ = 9.2 Hz, 1H), 8.05 (d, $^3J_{H-H}$ = 9.0 Hz, 1H), 8.04 (d, $^3J_{H-H}$ = 9.0 Hz, 1H), 8.00 (t, $^3J_{H-H}$ = 7.6 Hz, 1H), 7.90 (d, $^3J_{H-H}$ = 7.8 Hz, 1H), 3.76 (dd, $^3J_{H-H}$ = 8.4, 6.4 Hz, 1H), 3.42 – 3.30 (m, 2H), 2.05 – 1.96 (m, 2H), 1.93 – 1.74 (m, 4H), 1.71 – 1.67 (m, 3H), 1.51 – 1.50 (m, 1H), 1.20 (s, 6H), 1.13 (s,

3H); ^{13}C NMR (126 MHz, CD_2Cl_2): 137.2 (C), 131.8 (C), 131.4 (C), 130.1 (C), 129.0 (C), 127.9 (CH), 127.7 (CH), 127.5 (CH), 126.9 (CH), 126.2 (CH), 125.4 (2C), 125.3 (CH), 125.2 (CH), 125.0 (CH), 123.9 (CH), 85.2 (CH), 81.0 (C), 72.7 (C), 39.1 (CH_2), 37.9 (CH_2), 34.4 (CH_2), 30.1 (CH_2), 28.8 (CH_3), 28.2 (CH_3), 26.5 (CH_2), 26.3 (CH_2), 24.5 (CH_3); HRMS (ESI): calcd. for $C_{27}H_{30}O_2$ ([M+Na]$^+$): 409.2139, found: 409.2117.

Compound 3a. IR (neat): 3433 (w, O-H), 2968 (m, C-H), 2931 (m, C-H), 1458 (m, O-H), 1370 (m, C-H), 1046 (s, C-O), 1003 (s, C-O-C); ^{1}H NMR (500 MHz, CD_2Cl_2): 8.33 (d, $^3J_{H-H}$ = 9.2 Hz, 1H), 8.18 (d, $^3J_{H-H}$ = 7.6 Hz, 1H), 8.17 (d, $^3J_{H-H}$ = 7.6 Hz, 1H), 8.14 (d, $^3J_{H-H}$ = 7.8 Hz, 1H), 8.13 (d, $^3J_{H-H}$ = 9.2 Hz, 1H), 8.05 (d, $^3J_{H-H}$ = 9.0 Hz, 1H), 8.04 (d, $^3J_{H-H}$ = 9.0 Hz, 1H), 7.99 (t, $^3J_{H-H}$ = 7.6 Hz, 1H), 7.92 (d, $^3J_{H-H}$ = 7.8 Hz, 1H), 3.49 – 3.45 (m, 1H), 3.34 (t, $^3J_{H-H}$ = 7.6 Hz, 1H), 2.01 – 1.93 (m, 2H), 1.73 – 1.69 (m, 4H), 1.64 – 1.60 (m, 1H), 1.49 – 1.43 (m, 1H), 1.37 (d, $^3J_{H-H}$ = 5.7 Hz, 2H), 1.19 (s, 3H), 1.17 (s, 3H), 1.12 (s, 3H); ^{13}C NMR (126 MHz, CD_2Cl_2): 138.0 (C), 131.9 (C), 131.4 (CH), 130.1 (CH), 129.0 (CH), 127.9 (CH), 127.7 (CH), 127.4 (CH), 126.8 (CH), 126.2 (CH), 125.4 (2C), 125.2 (CH), 125.1 (CH), 125.0 (CH), 124.1 (CH), 76.6 (C), 72.5 (CH), 71.3 (C), 42.1 (CH_2), 36.3 (CH_2), 34.4 (CH_2), 32.3 (CH_3), 28.2 (CH_3), 25.9 (CH_2), 25.8 (CH_2), 21.4 (CH_3); HRMS (ESI): calcd. for $C_{27}H_{30}O_2$ ([M+Na]$^+$): 409.2139, found: 409.2117.

Compounds 4b and 4a. To a solution of **4** (16 mg, 41 µmol) in CH_2Cl_2 (220 µL), **20** (3 mg, 4 µmol) was added. Afterward, the mixture was stirred for 30 min at RT. Then, the reaction mixture was diluted with CH_2Cl_2 (0.5 mL) and washed with water (2 x 0.5 mL) and brine (2 x 0.2 ml). The organic phase was dried over Na_2SO_4 and concentrated under vacuum. The crude mixture was purified by preparative TLC (*n*-pentane/EtOAc 9:1, developed twice) to give compound **4b** (R_f 0.60, 1 mg, 8%) and compound **4a** (R_f 0.30, 13 mg, 82%) as colorless oils.

Compound 4b. IR (neat): 3453 (w, O-H), 2940 (w, C-H), 1467 (w, O-H), 1365 (w, C-H), 1264 (s, C-O), 1047 (m, C-O-C); ^{1}H NMR (500 MHz, CD_2Cl_2): 8.32 (d, $^3J_{H-H}$ = 9.2 Hz, 1H), 8.19 (d, $^3J_{H-H}$ = 7.6 Hz, 1H), 8.17 (d, $^3J_{H-H}$ = 7.6 Hz, 1H), 8.14 (d, $^3J_{H-H}$ = 7.8 Hz, 1H), 8.13 (d, $^3J_{H-H}$ = 9.2 Hz,

1H), 8.05 (d, $^3J_{H\text{-}H}$ = 9.0 Hz, 1H), 8.04 (d, $^3J_{H\text{-}H}$ = 9.0 Hz, 1H), 8.00 (t, $^3J_{H\text{-}H}$ = 7.6 Hz, 1H), 7.90 (d, $^3J_{H\text{-}H}$ = 7.8 Hz, 1H), 3.76 (dd, $^3J_{H\text{-}H}$ = 8.4, 6.4 Hz, 1H), 3.42 – 3.30 (m, 2H), 2.05 – 1.96 (m, 2H), 1.93 – 1.74 (m, 4H), 1.71 – 1.67 (m, 3H), 1.51 – 1.50 (m, 1H), 1.20 (s, 6H), 1.13 (s, 3H); ^{13}C NMR (126 MHz, CD_2Cl_2): 137.4 (C), 131.4 (C), 131.0 (C), 129.7 (C), 128.6 (C), 127.5 (CH), 127.3 (CH), 127.0 (CH), 126.4 (CH), 125.8 (CH), 125.0 (2C), 124.8 (CH), 124.7 (CH), 124.6 (CH), 123.6 (CH), 84.0 (CH), 80.7 (C), 72.3 (C), 40.4 (CH_2), 38.6 (CH_2), 34.0 (CH_2), 28.5 (CH_3), 27.8 (CH_3), 26.4 (CH_2), 26.1 (CH_2), 21.1 (CH_3); HRMS (ESI): calcd. for $C_{27}H_{30}O_2$ ([M+Na]$^+$): 409.2139, found: 409.2117.

Compound 4a. IR (neat): 3453 (w, O-H), 2972 (m, C-H), 2936 (m, C-H), 1467 (m, O-H), 1365 (m, C-H), 1264 (s, C-O), 1047 (s, C-O-C); ^{1}H NMR (500 MHz, CD_2Cl_2): 8.33 (d, $^3J_{H\text{-}H}$ = 9.2 Hz, 1H), 8.18 (d, $^3J_{H\text{-}H}$ = 7.6 Hz, 1H), 8.17 (d, $^3J_{H\text{-}H}$ = 7.6 Hz, 1H), 8.14 (d, $^3J_{H\text{-}H}$ = 7.8 Hz, 1H), 8.13 (d, $^3J_{H\text{-}H}$ = 9.2 Hz, 1H), 8.05 (d, $^3J_{H\text{-}H}$ = 9.0 Hz, 1H), 8.04 (d, $^3J_{H\text{-}H}$ = 9.0 Hz, 1H), 7.99 (t, $^3J_{H\text{-}H}$ = 7.6 Hz, 1H), 7.92 (d, $^3J_{H\text{-}H}$ = 7.8 Hz, 1H), 3.42 – 3.34 (m, 1H), 3.36 (t, $^3J_{H\text{-}H}$ = 7.8 Hz, 1H), 2.03 – 1.93 (m, 2H), 1.92 – 1.79 (m, 2H), 1.74 – 1.62 (m, 4H), 1.58 (d, $^3J_{H\text{-}H}$ = 6.7 Hz, 1H), 1.46 – 1.40 (m, 2H), 1.16 (s, 3H), 1.14 (s, 6H); ^{13}C NMR (126 MHz, CD_2Cl_2): 137.8 (C), 131.9 (C), 131.4 (C), 130.1 (C), 129.0 (C), 127.9 (CH), 127.8 (CH), 127.4 (CH), 126.8 (CH), 125.4 (2C), 125.2 (CH), 125.1 (CH), 125.0 (CH), 124.0 (CH), 76.6 (C), 72.8 (CH), 71.6 (C), 36.7 (CH_2), 34.4 (CH_2), 33.7 (CH_2), 30.6 (CH_3), 30.2 (CH_3), 26.0 (CH_2), 25.9 (CH_2), 24.9 (CH_3); HRMS (ESI): calcd. for $C_{27}H_{30}O_2$ ([M+Na]$^+$): 409.2139, found: 409.2117.

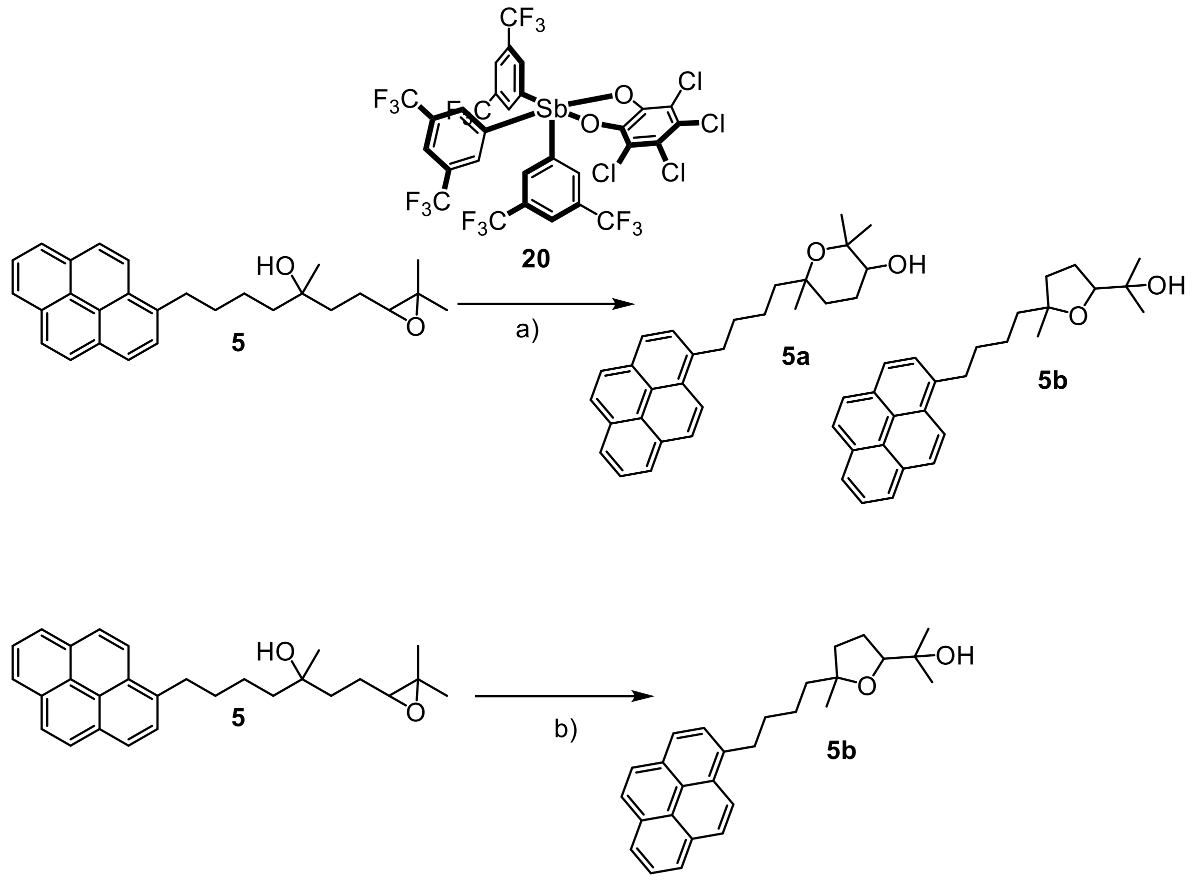

Scheme S6 (a) **20**, CH_2Cl_2, RT, 30 min, 85% **5a**, 15% **5b**. (b) AcOH, CH_2Cl_2, 2 h, RT, quant. **5b**.

Compound 5b. To a solution of **5** (20 mg, 50 μmol) in CH_2Cl_2 (3.3 mL), AcOH (330 μL, 5.7 mmol) was added. The mixture was stirred for 2 h at RT. The mixture was then washed with saturated $NaHCO_3$ until the pH of the aqueous phase was > 7, then, the organic phase was collected, dried over Na_2SO_4, and concentrated. The crude mixture was purified by successive preparative TLC (5, 10 and 15% EtOAc in *n*-pentane) to obtain two diastereomers **5b1** and **5b2** (~1:1) in quantitative yield.

Stereoisomer 5b1: R_f (*n*-pentane/EtOAc 7:1): 0.5; IR (neat): 3464 (b, O-H), 3040 (w, C-H), 2969 (s, C-H), 2934 (s, C-H), 2863 (s, C-H), 1603 (w, C-C), 1460, (m, C-C), 1372 (m, C-H), 1182 (m, C-O-C), 1147 (w, C-H), 1057 (s, C-O), 1033 (m, C-H), 952 (w, C-H); ^1H NMR (500 MHz, CD_2Cl_2): 8.31 (d, $^3J_{H\text{-}H}$ = 9.2 Hz, 1H), 8.20 – 8.16 (m, 2H), 8.14 – 8.11 (m, 1H), 8.12 (d, $^3J_{H\text{-}H}$ = 4.5 Hz, 1H), 8.07 – 7.98 (m, 3H), 7.90 (d, $^3J_{H\text{-}H}$ = 7.8 Hz, 1H), 3.72 – 3.69 (m, 1H), 3.39 – 3.35 (m, 2H),

2.07 (s, 1H), 1.90 – 1.76 (m, 4H), 1.70 – 1.49 (m, 6H), 1.18 (s, 3H), 1.15 (s, 3H), 1.07 (s, 3H); ^{13}C NMR (126 MHz, CD_2Cl_2): 137.8 (C), 131.8 (C), 131.4 (C), 130.1 (C), 129.0 (C), 127.9 (CH), 127.7 (CH), 127.4 (CH), 126.8 (CH), 126.2 (CH), 125.4 (C), 125.3 (C), 125.2 (CH), 125.2 (CH), 125.0 (CH), 124.0 (CH), 85.5 (CH), 83.5 (C), 70.2 (C), 40.9 (CH_2), 38.6 (CH_2), 35.0 (CH_2), 33.1 (CH_2), 27.8 (CH_3), 26.9 (CH_2), 26.8 (CH_3), 25.2 (CH_2), 24.3 (CH_3); HRMS (ESI): calcld. for $C_{28}H_{32}O_2$ ($[M+Na]^+$): 423.2295, found: 423.2284.

Stereoisomers 5b2: R_f (*n*-pentane/EtOAc 7:1): 0.37; IR (neat): 3460 (b, O-H), 3040 (w, C-H), 2968 (s, C-H), 2934 (s, C-H), 2862 (s, C-H), 1603 (w, C-C), 1587 (C-H), 1509 (w, C-H), 1460 (s, C-C), 1417 (w, C-H), 1372 (s, C-H), 1314 (w, C-H), 1243 (w, C-H), 1182 (s, C-O-C), 1089 (w, C-H), 1063 (s, C-O), 949 (m, C-H); ^{1}H NMR (500 MHz, CD_2Cl_2): 8.32 (d, $^3J_{H\text{-}H}$ = 9.3 Hz, 1H), 8.21 – 8.14 (m, 2H), 8.13 (d, $^3J_{H\text{-}H}$ = 3.7 Hz, 1H), 8.12 (d, $^3J_{H\text{-}H}$ = 5.2 Hz, 1H), 8.07 – 7.95 (m, 3H), 7.91 (d, $^3J_{H\text{-}H}$ = 7.9 Hz, 1H), 3.73 – 3.67 (m, 1H), 3.39 – 3.35 (m, 2H), 2.07 (s, 1H) 1.95 – 1.74 (m, 5H), 1.73 – 1.43 (m, 5H), 1.18 (s, 3H), 1.15 (s, 3H), 1.07 (s, 3H); ^{13}C NMR (126 MHz, CD_2Cl_2): 137.8 (C), 131.8 (C), 131.4 (C), 130.1 (C), 129.0 (C), 127.9 (CH), 127.7 (CH), 127.4 (CH), 126.8 (CH), 126.2 (CH), 125.4 (C), 125.3 (C), 125.2 (CH), 125.1 (CH), 125.0 (CH), 124.0 (CH), 85.0 (CH), 83.5 (C), 71.3 (C), 42.1 (CH_2), 37.3 (CH_2), 35.3 (CH_2), 32.8 (CH_2), 27.5 (CH_3), 26.8 (CH_2), 25.7 (CH_3), 25.4 (CH_2), 24.5 (CH_3); HRMS (ESI): calcld. for $C_{28}H_{32}O_2$ ($[M+Na]^+$): 423.2295, found: 423.2284.

Compound 5a. To a solution of **5** (50 mg, 130 μmol) in CH_2Cl_2 (10 mL) was added **20** (10 mg, 13 μmol). The mixture was stirred at RT for 2 h, when complete consumption of starting material was observed, and washed with water. The combined organic phases were dried over Na_2SO_4 and concentrated *in vacuo.* The product ratio (**5a**/**5b** 17:3) was determined by an analytical HPLC profile of the crude mixture. The crude mixture was then purified by preparative TLC (CH_2Cl_2, developed four times) to afford stereoisomers **5a1** (R_f 0.5), **5a2** (R_f 0.33), and **5b** (R_f 0.6).

Stereoisomer 5a1: R_f (CH_2Cl_2, devloped four times): 0.5; IR (neat): 3426 (b, O-H), 3040 (w, C-H), 2931 (s, C-H), 2856 (w, C-H), 1603 (w, C-C), 1459 (m, C-C), 1373 (m, C-H), 1243 (w, C-H), 1182 (w, C-H), 1106 (m. C-H), 1060 (m, C-O-C), 1016 (m. C-O), 985 (m, C-H); ^{1}H NMR (500 MHz,

CD_2Cl_2): 8.31 (d, $^3J_{H\text{-}H}$ = 9.3 Hz, 1H), 8.20 – 8.16 (m, 2H), 8.13 (d, $^3J_{H\text{-}H}$ = 4.3 Hz, 1H), 8.11 (d, $^3J_{H\text{-}H}$ = 5.8 Hz, 1H), 8.08 – 7.96 (m, 3H), 7.90 (d, $^3J_{H\text{-}H}$ = 7.7 Hz, 1H), 3.39 – 3.31 (m, 3H), 1.91 – 1.77 (m, 2H), 1.77 – 1.67 (m, 2H), 1.62 – 1.43 (m, 6H), 1.19 (s, 3H), 1.19 (s, 6H); ^{13}C NMR (126 MHz, CD_2Cl_2): 138.0 (C), 131.8 (C), 131.4 (C), 130.1 (C), 129.0 (C), 127.9 (CH), 127.7 (CH), 127.4 (CH), 126.8 (CH), 126.2 (CH), 125.4 (C), 125.3 (C), 125.2 (CH), 125.1 (CH), 125.0 (CH), 124.0 (CH), 75.0 (CH), 75.0 (C), 73.3 (C), 44.9 (CH_2), 34.4 (CH_2), 34.0 (CH_2), 33.0 (CH_2), 29.9 (CH_3), 26.3 (CH_3), 25.6 (CH_2), 24.3 (CH_2), 22.8 (CH_3); HRMS (ESI): calcld. for $C_{28}H_{32}O_2$ ([M+Na]$^+$): 423.2295, found: 423.2284.

Stereoisomer 5a2: R_f (CH_2Cl_2, developed four times): 0.33; IR(neat): 3424 (b, O-H), 3040 (w, C-H), 2936 (S, C-H), 2863 (w, C-H), 1603 (w, C-C), 1464 (m, C-C), 1372 (m. C-H), 1216 (w, C-H), 1182 (w, C-H), 1117 (s, C-O-C), 1064 (s, C-O), 1014 (m, C-H), 980 (m, C-H); ^{1}H NMR (500 MHz, CD_2Cl_2): 8.31 (d, $^3J_{H\text{-}H}$ = 9.2 Hz, 1H), 8.21 – 8.15 (m, 2H), 8.14 – 8.11 (m, 1H), 8.08 – 7.97 (m, 3H), 7.91 (d, $^3J_{H\text{-}H}$ = 7.8 Hz, 1H), 3.42 – 3.33 (m, 3H), 1.92 – 1.80 (m, 2H), 1.76 – 1.35 (m, 8H), 1.22 (s, 3H), 1.15 (s, 3H), 1.14 (s, 3H); ^{13}C NMR (126 MHz, CD_2Cl_2): 137.9 (C), 131.8 (C), 131.4 (C), 130.1 (C), 129.0 (C), 127.9 (CH), 127.7 (CH), 127.4 (CH), 126.8 (CH), 126.2 (CH), 125.4 (C), 125.3 (C), 125.2 (CH), 125.1 (CH), 125.0 (CH), 124.0 (CH), 74.8 (C), 73.5 (C), 73.0 (CH), 42.7 (CH_2), 34.0 (CH_2), 33.0 (CH_2), 32.2 (CH_2), 29.2 (CH_3), 28.0 (CH_3), 24.9 (CH_2), 24.7 (CH_2), 24.5 (CH_3); HRMS (ESI): calcld. for $C_{28}H_{32}O_2$ ([M+Na]$^+$): 423.2295, found: 423.2284.

3. Catalysis on MWCNTs in suspension

The conversion of **2** (100 mM) in the presence of MWCNTs (0, 1, 3, or 9 wt%) in ODCB at 40 °C was monitored as a function of time. Samples (1 drop ~ 5 µL) were taken in appropriate intervals, diluted in *n*-hexane, and analyzed by HPLC. Peaks of substrate **2** (red) and product **2b** (blue) were integrated to determine the conversion. HPLC conditions: λ_{abs} 342 nm; YMC-Pack SIL 5 × 50 mm, 120 Å, 3 µm; 0.8 mL/min; 3:7 (EtOAc + 1% Et_3N)/*n*-hexane. R_t (**2**): 6.5 min and R_t (**2b**): 2.5 min.

The concentrations of consumed substrate (**2**) were plotted against reaction time and fit to a linear function to determine initial velocities (v_{ini}, Fig. 3a). Apparent initial first-order rate constants (Table S1) were determined from Equation (S1)

$$k_{app} = v_{ini} / ([\text{epoxide}]_0) \qquad \text{(S1)}$$

where k_{app} corresponds to the first-order catalytic rate constant (k_{cat}) in the presence of MWCNTs. The first-order rate constant of the uncatalyzed reaction k_{uncat} (**2**) = 6.7 x 10^{-5} h^{-1}.

Table S1 Cyclization of substrate **2** on MWCNT suspensions in ODCB.[a]

Entry	MWCNT (wt%)[b]	k_{cat} (10^{-4} h^{-1})[c]	k_{cat}/k_{uncat} [d]
1	1	2.4	4
2	3	5.6	8
3	9	37	55

[a]Conditions: following the general procedure, 100 mM **2**, 0-9 wt% MWCNT, ODCB, 40 °C. [b]Weight percent MWCNTs in ODCB. [c]Catalytic rate constant. [d]Rate enhancement.

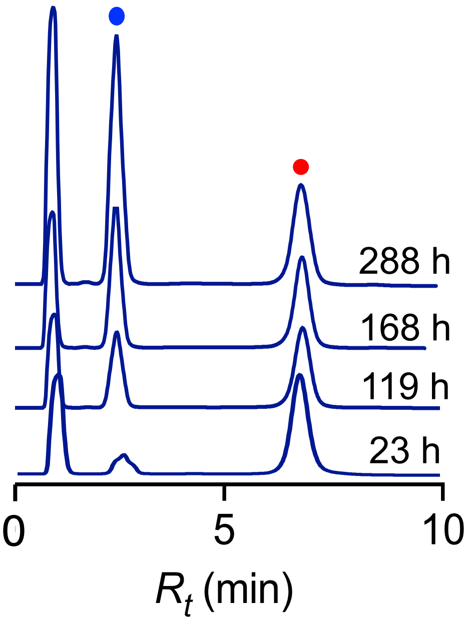

Fig. S3 Representative HPLC profile showing the time course of the conversion of **2** (red) into **2b** (blue) in the presence of MWCNTs (9% wt) in ODCB at 40 °C.

4. Microfluidic electric-field catalysis

4.1. General procedures

The reactions were performed using an ion electrochemical reactor from Vapourtec (FEP spacer, 0.25 mm; reactor volume, 0.3 mL; Pt and Gr*: MWCNT-coated Gr electrodes). The coated graphite electrode was prepared following an established drop-casting protocol.[S7] Solutions of the epoxides (**2**, **3**, **4** or **5**) at varying concentrations (25, 50 or 100 mM) in solvents of different polarities were infused at chosen flow rates (25 – 15 µL/min) into the electrochemical reactor under constant current. The first one and a half reactor volume (0.45 mL) were disposed to ensure that a steady state of the system had been reached. After collection for a defined period, the reaction mixture (~5 µL) was diluted in hexane and analyzed by HPLC. The substrate conversion was determined by comparing the area% of pertinent peak (**2**: 6.5 min; **3:** 10 min, **4:** 9.9 min, **5**: 9.2 min) with that of the corresponding products (**2b:** 2.5 min, **3a**: 2.0 min, **3b**: 4.6 min, **4a**: 2.1 min, **4b**: 4.8 min, **5a**: 5.2 min and **5b**: 2.5 min) in the crude HPLC profile under following conditions: λ_{abs} 342 nm; YMC-Pack SIL 5 × 50 mm, 120 Å, 3 µm; 0.8 mL/min; 3:7 (EtOAc + 1% Et_3N)/*n*-hexane for **2**, 15:85 (EtOAc + 1% Et_3N)/*n*-hexane for **3** – **4** and 2:8 (EtOAc + 1% Et_3N)/*n*-hexane for **5**. Experimental duplicates indicate the error levels to be within 10% of reported values.

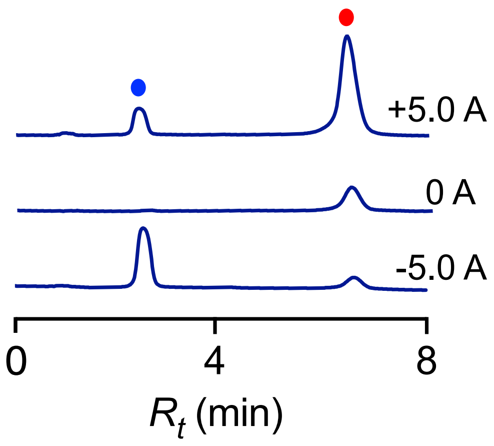

Fig. S4 Representative HPLC profile showing the cyclization of **2** (25 mM) under positive/negative applied field in dry PC. Peaks of product **2b** (blue, R_t: 2.5 min) and substrate **2** (red, R_t: 6.5 min) were integrated to determine conversion.

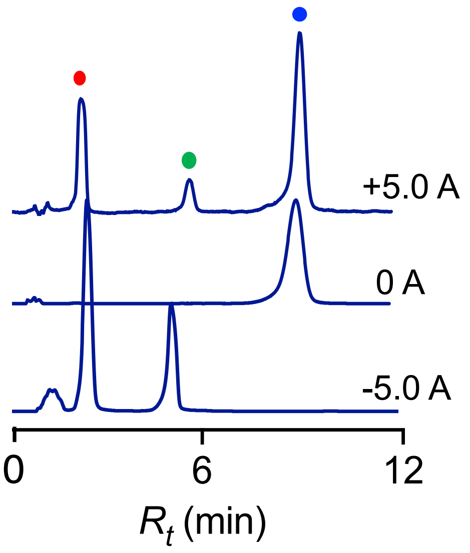

Fig. S5 Representative HPLC profile showing the cyclization of **3** (25 mM) under positive/negative applied field in dry PC. Peaks of substrate **3** (blue, R_t: 10 min) and products, **3b** (red, R_t: 2 min) and **3a** (green, R_t: 4.6 min), were integrated to determine conversion.

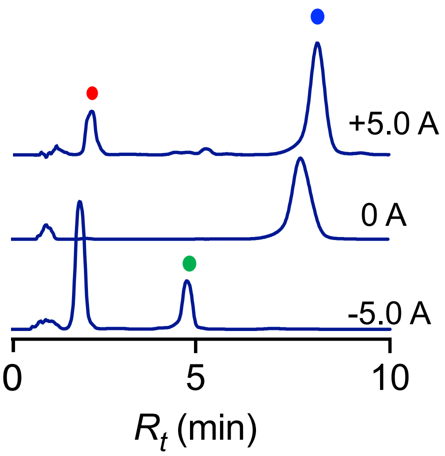

Fig. S6 Representative HPLC profile showing the cyclization of **4** (25 mM) under positive/negative applied field in dry PC. Peaks of substrate **4** (blue, R_t: 8 min) and products, **4b** (red, R_t: 2 min) and **4a** (green, R_t: 4.8 min), were integrated to determine conversion.

4.2. Dependence on solvents

Table S2 OEEF catalyzed cyclization of **2** (100 mM) in different solvents at 25 µL/min.[a]

Entry	(+)/(-)[b]	I (A)[c]	V (V)[d]	η (%)[e]	Solvents[f]	ε[g]
1	Pt/Gr*	0	-	1	C_6F_6	2
2	Pt/Gr*	0.2	1.3	1	C_6F_6	2
3	Pt/Gr*	0.5	2.6 – 2.4	1	C_6F_6	2
4	Pt/Gr*	1.5	3.2 – 3.6	6	C_6F_6	2
5	Pt/Gr*	5.0	3.4 – 3.5	12	C_6F_6	2
6	Gr*/Pt	0	-	3	Toluene	3
7	Gr*/Pt	0.5	1.3	2	Toluene	3
8	Gr*/Pt	1.5	2.9 – 2.8	2	Toluene	3
9	Gr*/Pt	3.0	4.4 – 4.5	3	Toluene	3
10	Gr*/Pt	5.0	4.9 – 5.0	8	Toluene	3
11	Pt/Gr*	1.0	2.3	1	Toluene	3
12	Pt/Gr*	3.0	3.9	4	Toluene	3
13	Pt/Gr*	5.0	3.5	10	Toluene	3
14[h]	Gr*/Pt	0	-	1	PC	64
15[h]	Gr*/Pt	5	1.7 – 1.6	16	PC	64
16[h]	Pt/Gr*	1.5	0.8 – 0.6	1	PC	64
17[h]	Pt/Gr*	3.0	1.2 – 1.1	20	PC	64
18[h]	Pt/Gr*	5.0	1.8 – 1.7	80	PC	64
19[h]	Pt/Gr*	0	-	1	DMSO	47
20[h]	Pt/Gr*	1.5	1.0	1	DMSO	47
21[h]	Pt/Gr*	3.0	1.8 – 1.9	19	DMSO	47

22[h]	Pt/Gr*	5.0	2.9 – 2.8	48	DMSO	47

[a]Conditions: Following the general procedure. [b]Electrodes configuration (anode)/(cathode). Gr*: MWCNT coated graphite. [c]Current applied, in ampere. [d]Range of measured voltage, in volt. [e]Total conversion of substrate, **2**. [f]Solvent used. [g]Dielectric constant of solvent. Conversions were calculated from obtained HPLC profiles (Fig. S3). Epoxide substrate was only converted into THF **2b** cyclic product. [h]These experiments were done with a 50 mM solution of substrate and the flow rate of 15 μL/min.

4.3. Dependence on water

Table S3. OEEF ion-catalyzed cyclization of **3** (25 mM) in dry PC with increasing H_2O at 15 μL/min.[a]

Entry	(+)/(-)[b]	*I* (A)[c]	*V* (V)[d]	*η* (%)[e]	H_2O (eq)[f]	P (%)[g] **3b**	**3a**
1	Pt/Gr*	0	-	1	0	1	-
2	Gr*/Pt	1.5	1.6 – 1.7	4	0	3	1
3	Gr*/Pt	3.0	2.5 – 2.6	6	0	6	-
4	Gr*/Pt	5.0	3.2 – 3.1	37	0	30	7
5	Pt/Gr*	1.5	1.4	100	0	72	28
6	Pt/Gr*	3.0	2.4	100	0	70	30
7	Pt/Gr*	5.0	3.1	100	0	65	35
8	Pt/Gr*	0	-	8	1	8	-
9	Gr*/Pt	5	2.4	7	1	6	1
10	Pt/Gr*	1.5	1.0 – 0.9	9	1	7	2
11	Pt/Gr*	3.0	1.7	93	1	64	19
12	Pt/Gr*	5	2.5 – 2.4	99	1	68	31
13	Pt/Gr*	0	-	2	10	2	-
14	Gr*/Pt	5.0	2.8 – 2.7	12	10	9	3
15	Pt/Gr*	1.5	1.1	2	10	2	-
16	Pt/Gr*	3.0	1.9	98	10	68	30
17	Pt/Gr*	5.0	2.8 – 2.6	100	10	67	33

[a]Conditions: following the general procedure. [b]Electrodes configuration (anode)/(cathode). Gr*: MWCNT coated graphite. [c]Current applied, in ampere. [d]Range of measured voltage, in volt. [e]Total conversion of substrate **3** after one passage through the electromicrofluidic reactor. [f]Equivalents of added H_2O. [g]Yields of assigned cyclic products **3b** and **3a**. (-) indicates < 0.8% yield.

4.4. Dependence on substrates

Table S4 Results of OEEF ion-π catalyzed cyclization of substrate (25 mM) in different solvents at 15 μL/min.[a]

Entry	S[b]	(+)/(-)[c]	*I* (A)[d]	*V* (V)[e]	*η* (%)[f]	Solvent[g]	P (%)[h]	
							**b**	**a**
1	**3**	Gr*/Pt	0	-	1	PC	1	-
2	**3**	Gr*/Pt	1.5	1.4	4	PC	3	1
3	**3**	Gr*/Pt	5.0	3.1 – 3.2	37	PC	30	7
4	**3**	Pt/Gr*	1.5	1.7	100	PC	72	28
5	**3**	Pt/Gr*	3.0	2.6	100	PC	70	30
6	**3**	Pt/Gr*	5.0	3.1 – 3.2	100	PC	65	35
7	**4**	Gr*/Pt	0	-	9	PC	8	1
8	**4**	Gr*/Pt	5	3.2	22	PC	20	2
9	**4**	Pt/Gr*	1.5	1.8 – 1.9	92	PC	73	19
10	**4**	Pt/Gr*	3.0	2.5	84	PC	65	19
11	**4**	Pt/Gr*	5	3.2	100	PC	71	29
12	**5**	Gr*/Pt	0	-	7	PC	6	1
13	**5**	Gr*/Pt	3.0	1.8-1.9	7	PC	7	-
14	**5**	Gr*/Pt	5.0	2.5-2.2	8	PC	8	-
15	**5**	Pt/Gr*	0.8	0.6	4	PC	4	-
16	**5**	Pt/Gr*	3.0	1.6	100	PC	77	23
17	**5**	Pt/Gr*	5.0	2.3-2.2	100	PC	76	24

(continued)

Entry	S[b]	(+)/(-)[c]	I (A)[d]	V (V)[e]	η (%)[f]	Solvent[g]	P (%)[h] **b**	**a**
18	**5**	Gr*/Pt	0	-	8	CH_3CN	8	-
19	**5**	Gr*/Pt	3.0	1.4	11	CH_3CN	11	-
20	**5**	Gr*/Pt	5.0	2.1	15	CH_3CN	14	1
21	**5**	Pt/Gr*	0.2	0.4	10	CH_3CN	9	1
22	**5**	Pt/Gr*	0.8	1.3	31	CH_3CN	26	5
23	**5**	Pt/Gr*	3.0	3.3 – 3.7	88	CH_3CN	68	20
24	**5**	Pt/Gr*	5.0	3.5 – 3.8	96	CH_3CN	69	27
25	**5**	Gr*/Pt	0	-	7	CH_3CN	7	-
26	**5**	Gr*/Pt	0.41	2.0 – 2.1	6	CH_3CN	6	-
27	**5**	Gr*/Pt	1.43	3.1 – 3.5	8	CH_3CN	8	-
28	**5**	Pt/Gr*	0.41	1.7 – 1.6	70	CH_3CN	57	13
29	**5**	Pt/Gr*	1.43	3.3 – 3.4	59	CH_3CN	49	10
30	**5**	Pt/Gr*	3.5	3.7 – 3.3	44	CH_3CN	37	7

[a]Conditions: following the general procedure. Conditions for entries 25-30 are the same as 18-24 but using refreshed electrodes and the substrate solution (experimental replicates). [b]Substrate. [c]Electrodes configuration (anode)/(cathode). Gr*: MWCNT coated graphite. [d]Current applied, in ampere. [e]Range of measured voltage, in volt. [f]Total conversion of the substrate after one passage through the electromicrofluidic reactor. [g]Solvents. [h]Yields of assigned cyclic products **b** (**3b**, **4b**, **5b**) and **a** (**3a**, **4a**, **5a**) after one passage through the electromicrofluidic reactor. (-) indicates < 0.8% yield. Conversions and yields were calculated from obtained HPLC profiles (Fig. S4-S6).

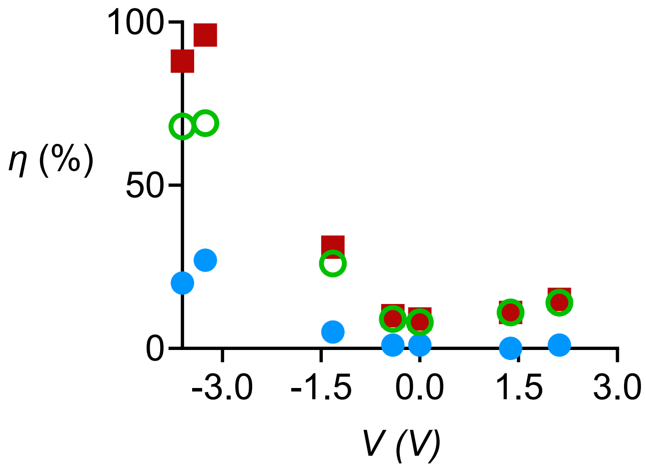

Fig. S7 Yield (η) of **5a** (blue filled circles), **5b** (green empty circles) and total yield (red square) as a function of the applied voltage obtained from **5** (25 mM) in dry CH_3CN passing once through the electromicrofluidic reactor (15 μL/min, Pt/Gr* electrodes).

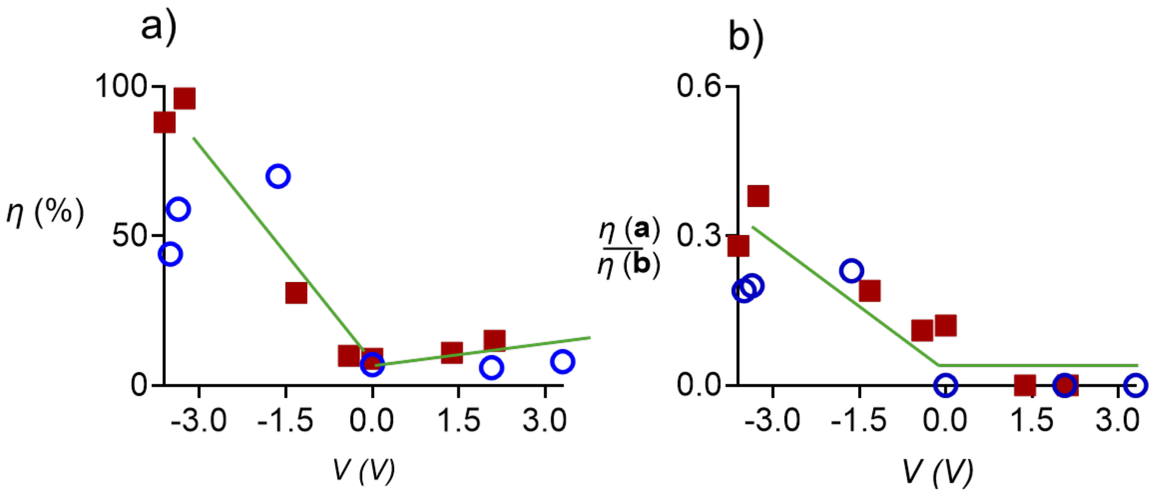

Fig. S8 Total yield (η) of cyclization products **5a** and **5b** (a) and **5a/5b** ratio (b) as a function of the applied voltage obtained from **5** (25 mM) in dry CH_3CN passing once through the electromicrofluidic reactor (15 μL/min, Pt/Gr* electrodes). Experimental duplicates (red squares and blue empty circles) using different batches of electrodes and substrate solutions are shown. Green lines are added only to guide the eyes.

5. Supplementary references

S1 M. Á. Gutiérrez López, R. Ali, M.-L. Tan, N. Sakai, T. Wirth and S. Matile, *Sci. Adv.*, 2023, **9**, eadj5502.

S2 F. Colobert, T. Kreuzer, J. Cossy, S. Reymond, T. Tsuchiya, L. Ferrié, I. Marko and P. Jourdain, *Synlett*, 2007, **15**, 2351–2354.

S3 V. Morozova, J. Skotnitzki, K. Moriya, K. Karaghiosoff and P. Knochel, *Angew. Chem. Int. Ed.*, 2018, **57**, 5516–5519.

S4 M. Á. Gutiérrez López, M.-L. Tan, A. Frontera and S. Matile, *JACS Au*, 2023, **3**, 1039–1051.

S5 H. H. Patel and M. S. Sigman, *J. Am. Chem. Soc.*, 2016, **43**, 14226–14229.

S6 A. Taher, K. C. Lee, H. J. Han and D. W. Kim, *Org. Lett.*, 2017, **19**, 3342–3345.

S7 G. Gabriel, R. Gómez-Martínez and R. Villa, *Physiol. Meas.*, 2008, **29**, S203-S212.

6. NMR spectra

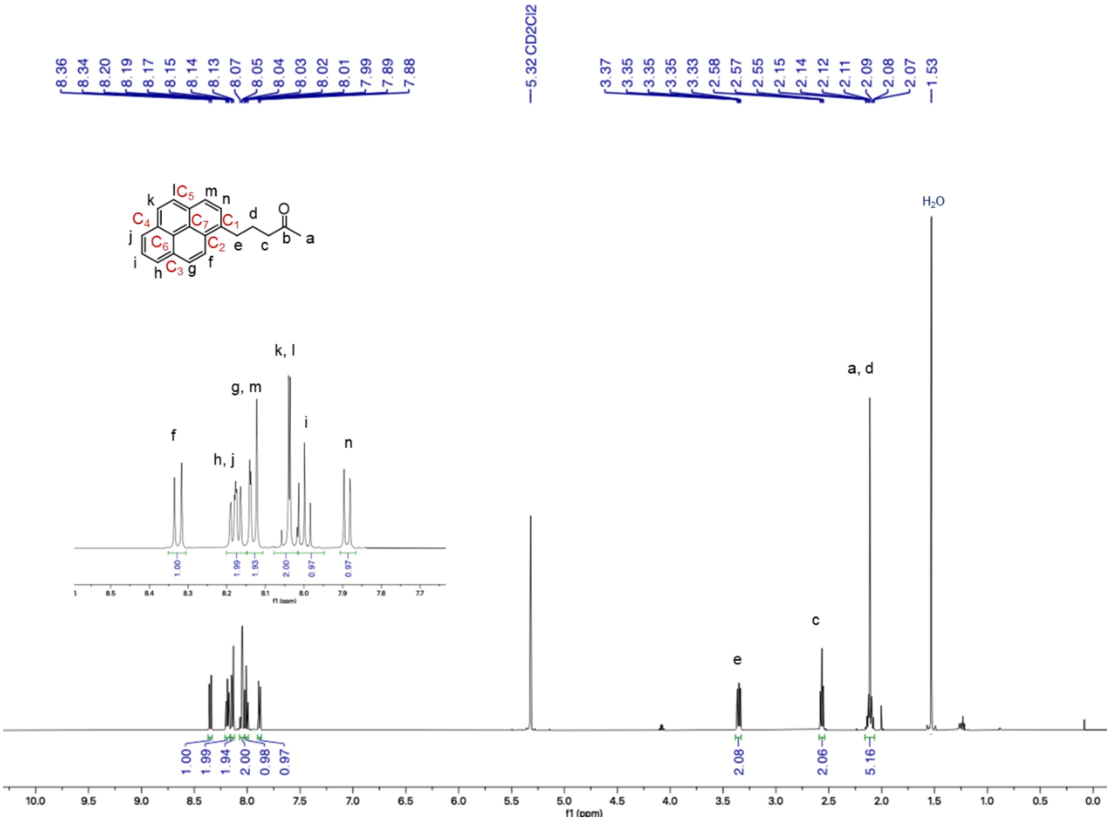

Fig. S9 500 MHz ^{1}H NMR spectrum of **11** in CD_2Cl_2.

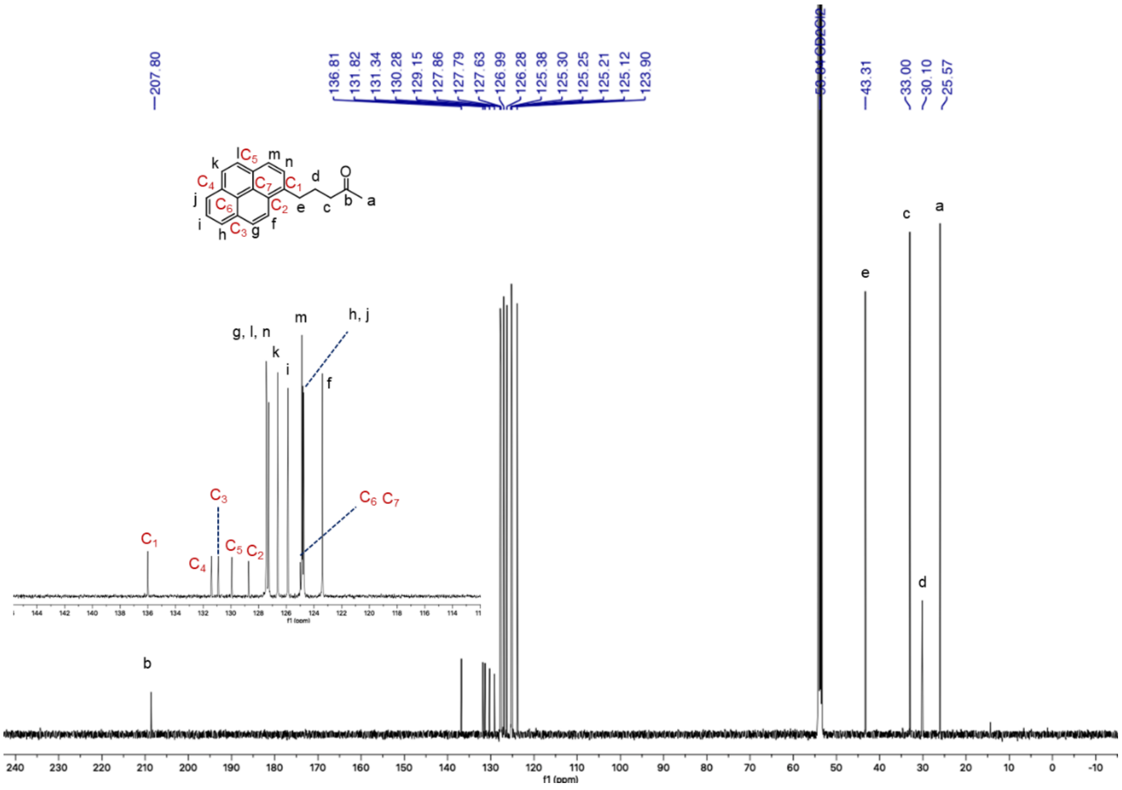

Fig. S10 126 MHz ^{13}C NMR spectrum of **11** in CD_2Cl_2.

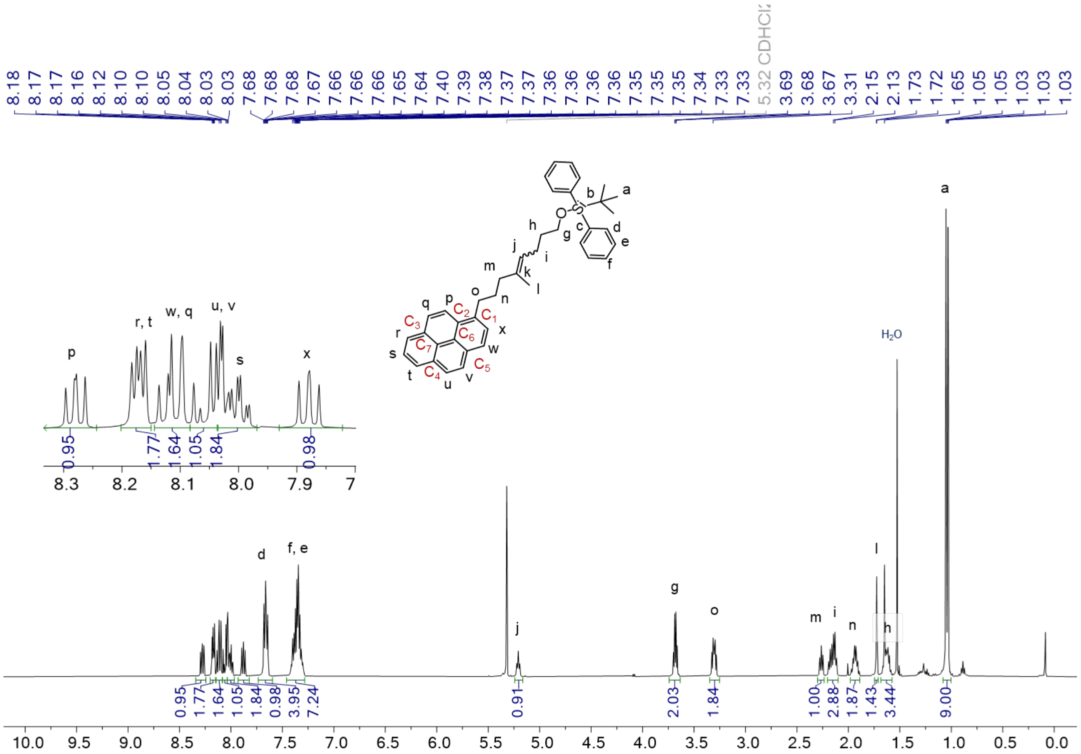

Fig. S11 500 MHz ^{1}H NMR spectrum of **12** in CD_2Cl_2.

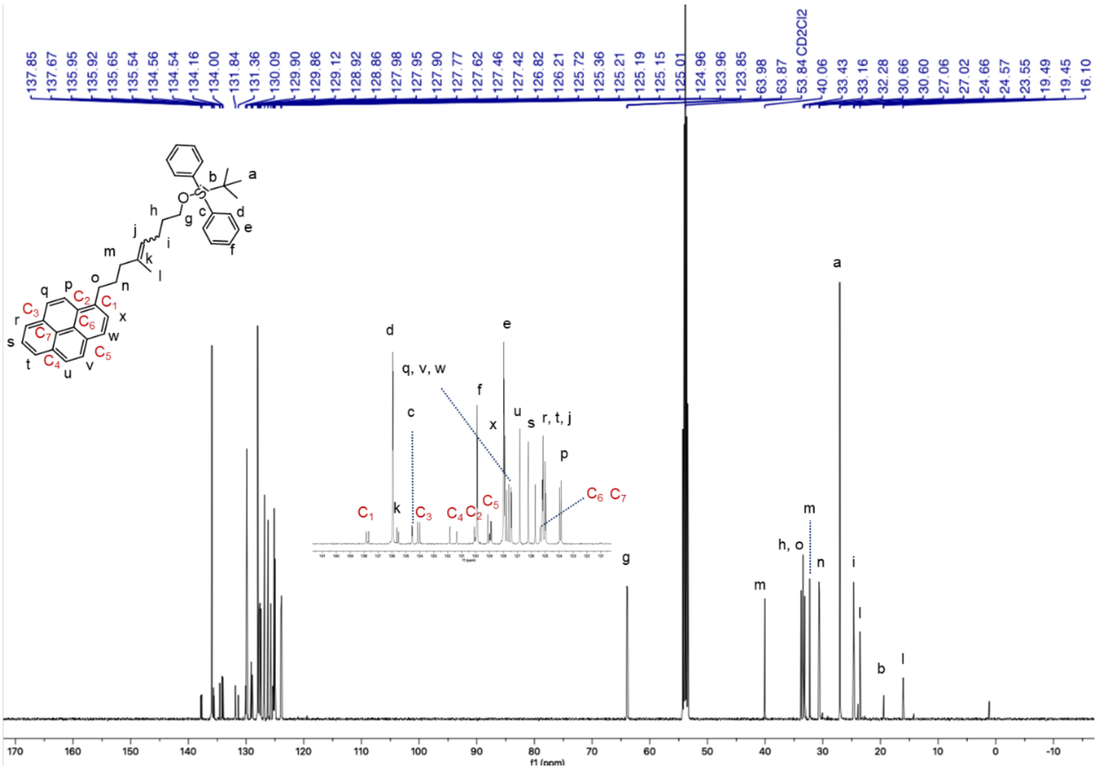

Fig. S12 126 MHz ^{13}C NMR spectrum of **12** in CD_2Cl_2.

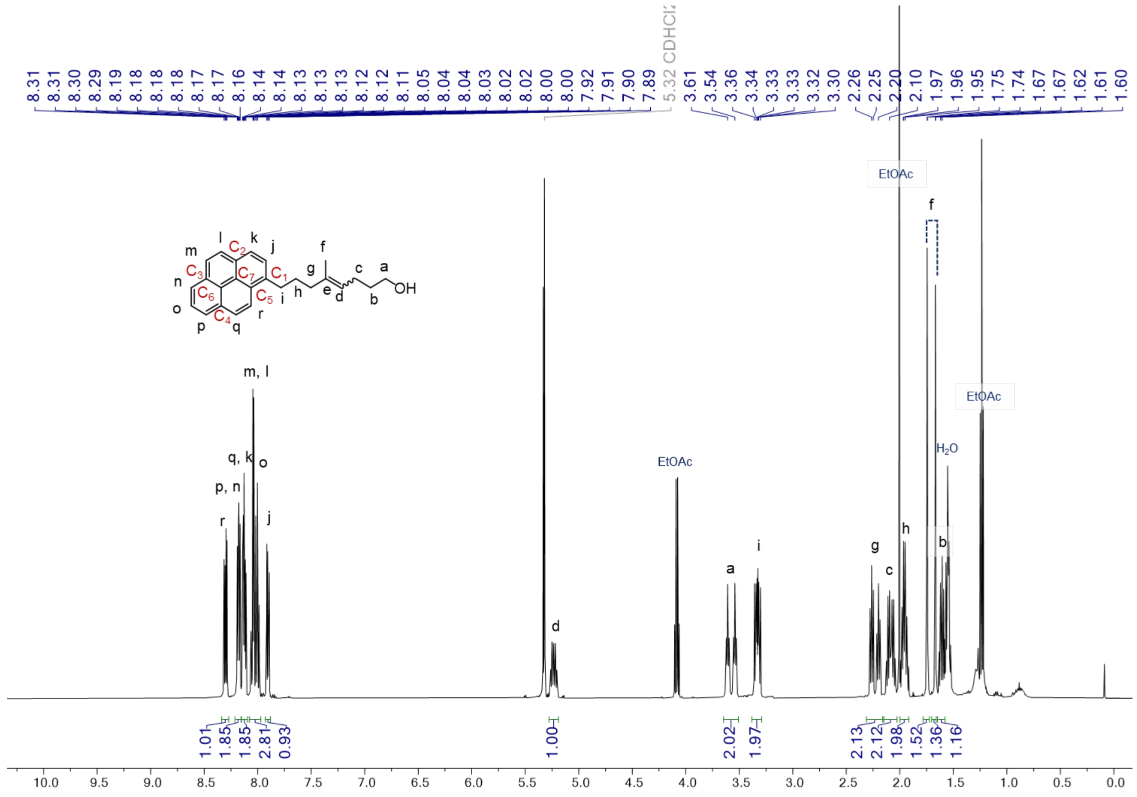

Fig. S13 500 MHz ^1H NMR spectrum of **13** in CD_2Cl_2.

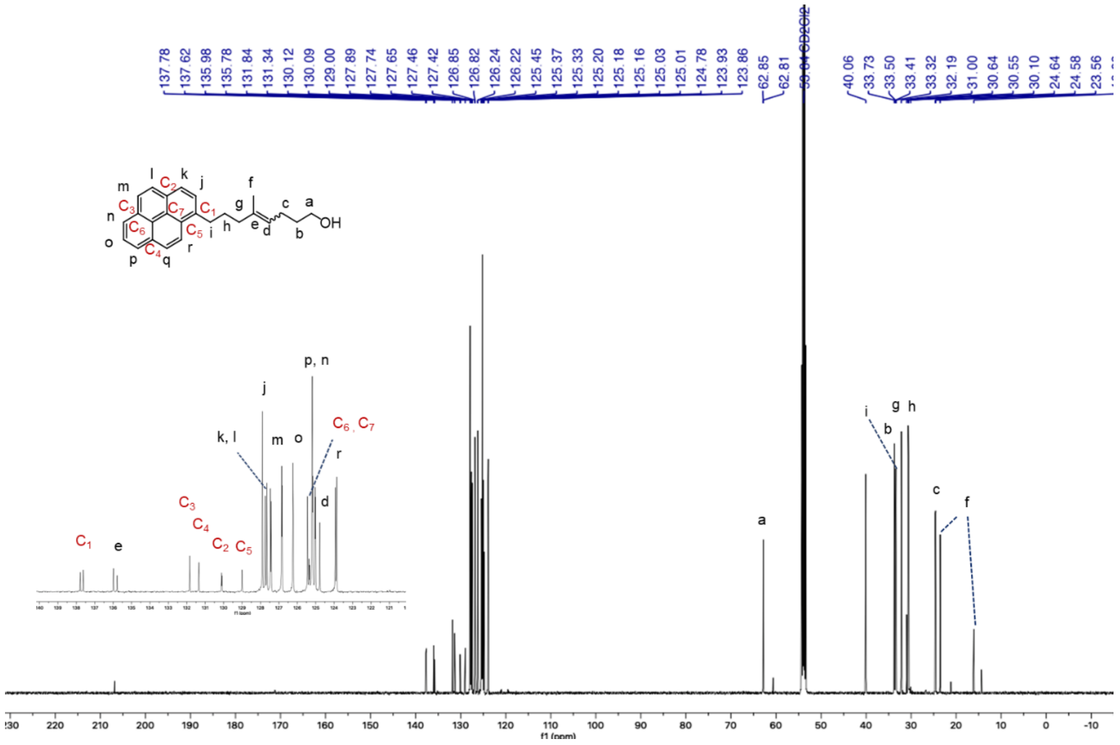

Fig. S14 126 MHz ^{13}C NMR spectrum of **13** in CD_2Cl_2.

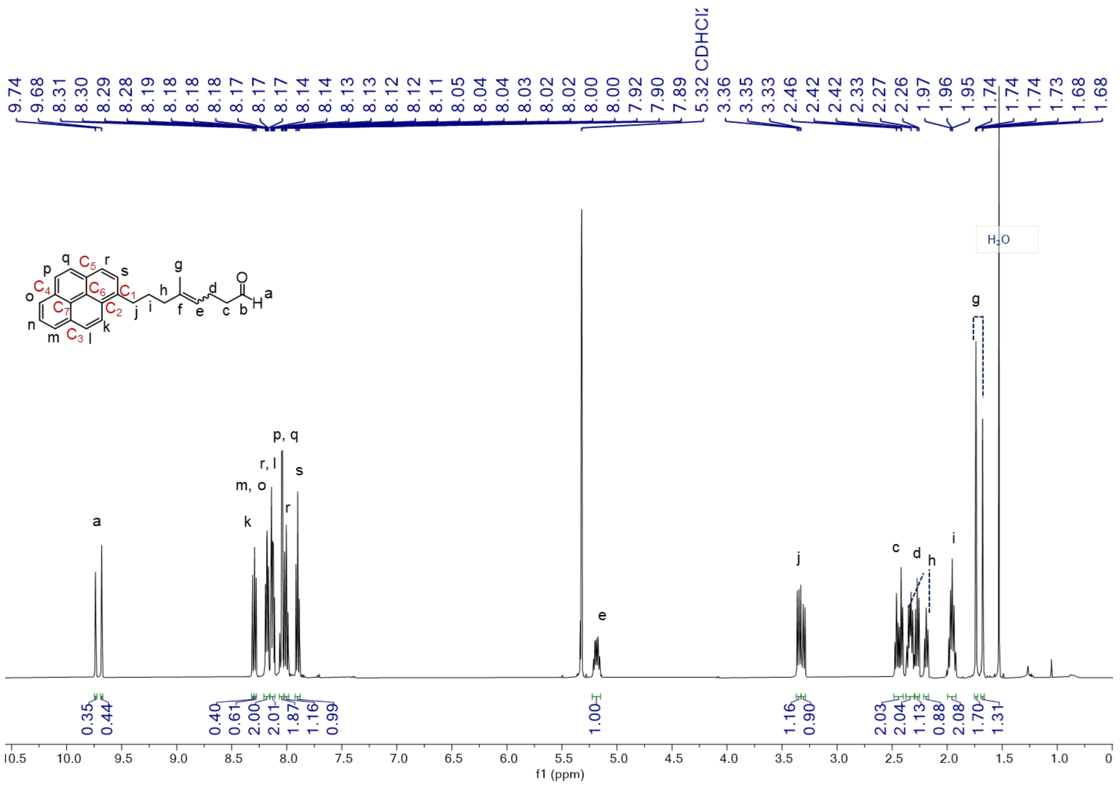

Fig. S15 500 MHz ^1H NMR spectrum of **14** in CD_2Cl_2.

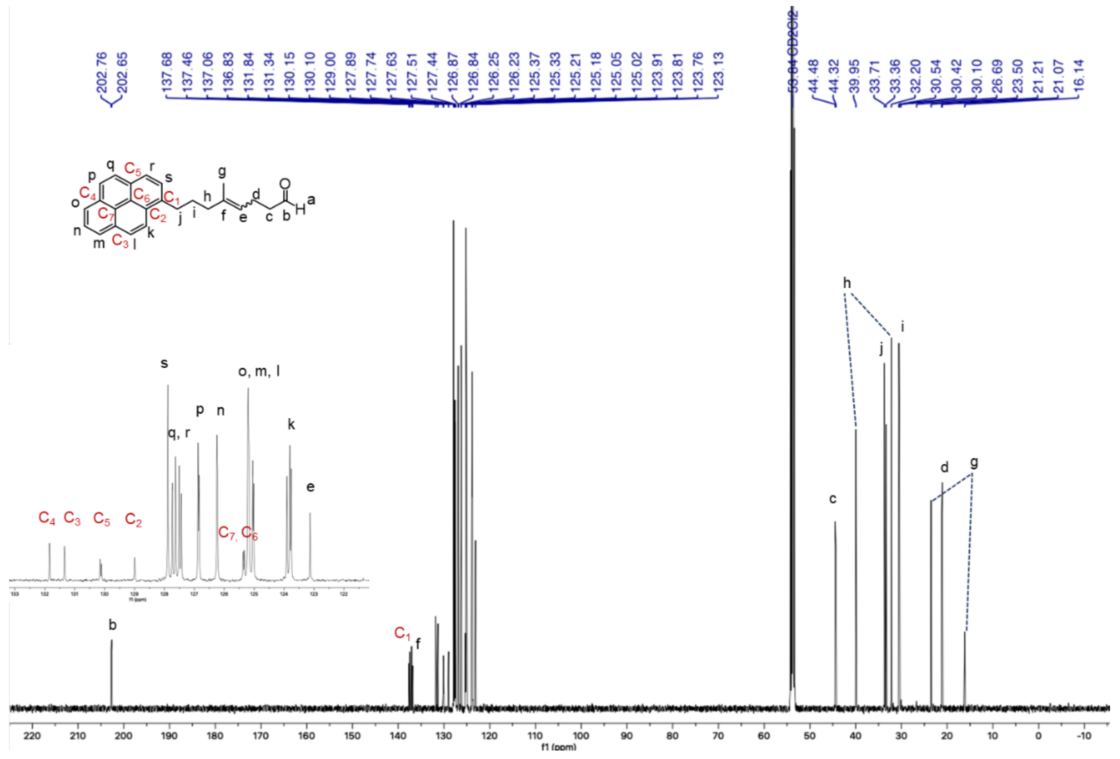

Fig. S16 126 MHz ^{13}C NMR spectrum of **14** in CD_2Cl_2.

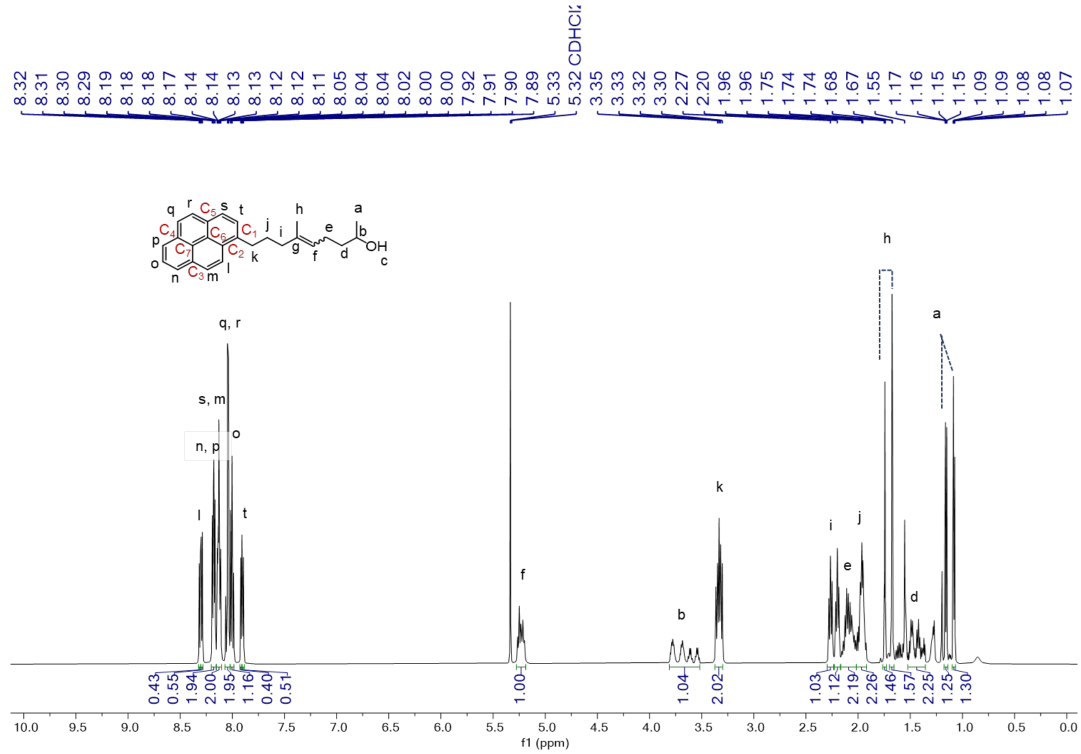

Fig. S17 500 MHz ^{1}H NMR spectrum of **15** in CD_2Cl_2.

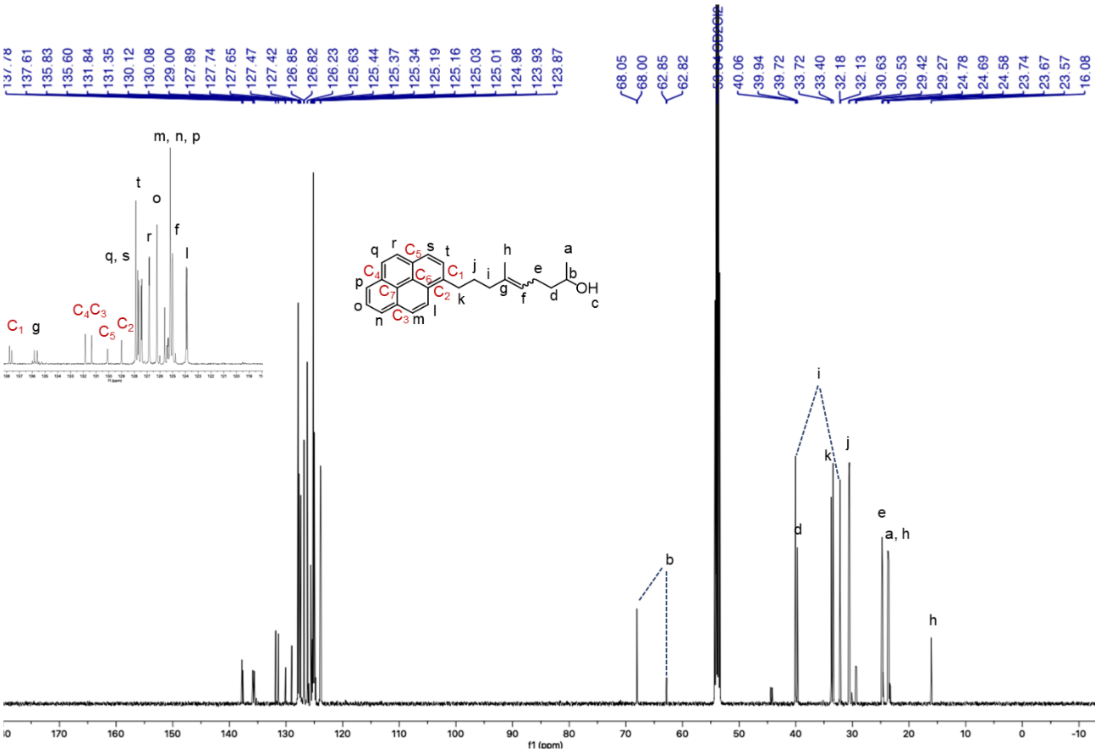

Fig. S18 126 MHz ^{13}C NMR spectrum of **15** in CD_2Cl_2.

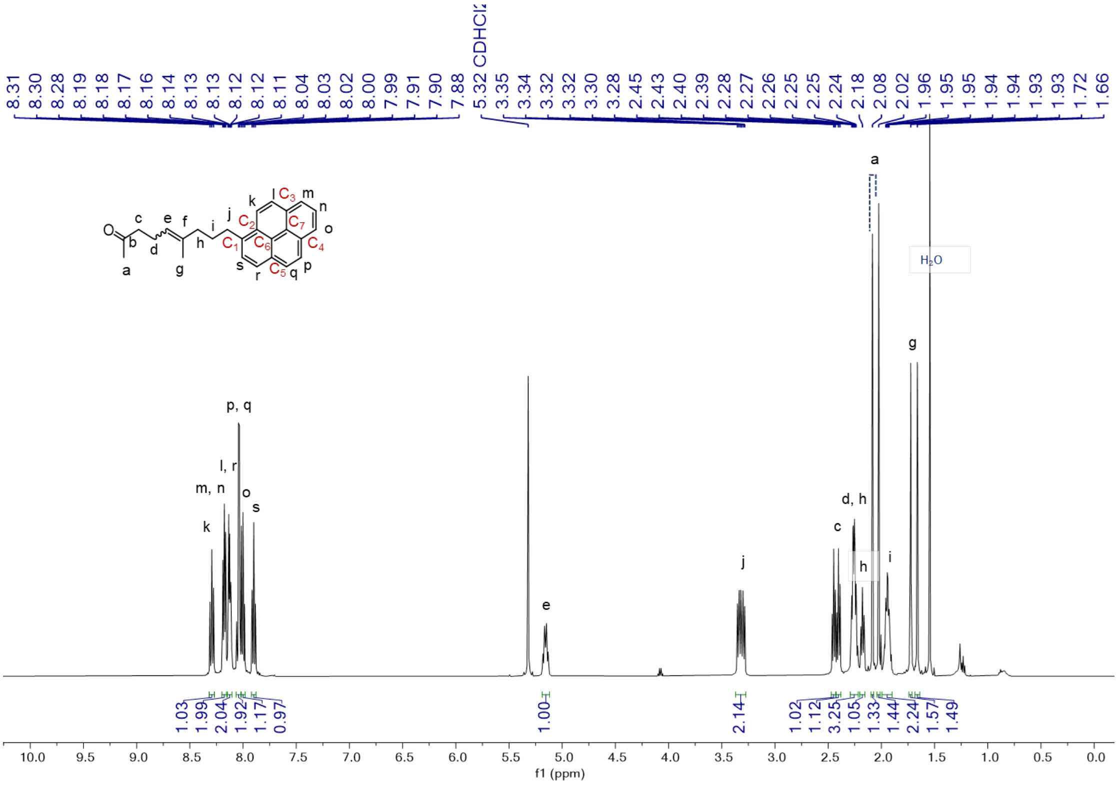

Fig. S19 500 MHz ^{1}H NMR spectrum of **16** in CD_2Cl_2.

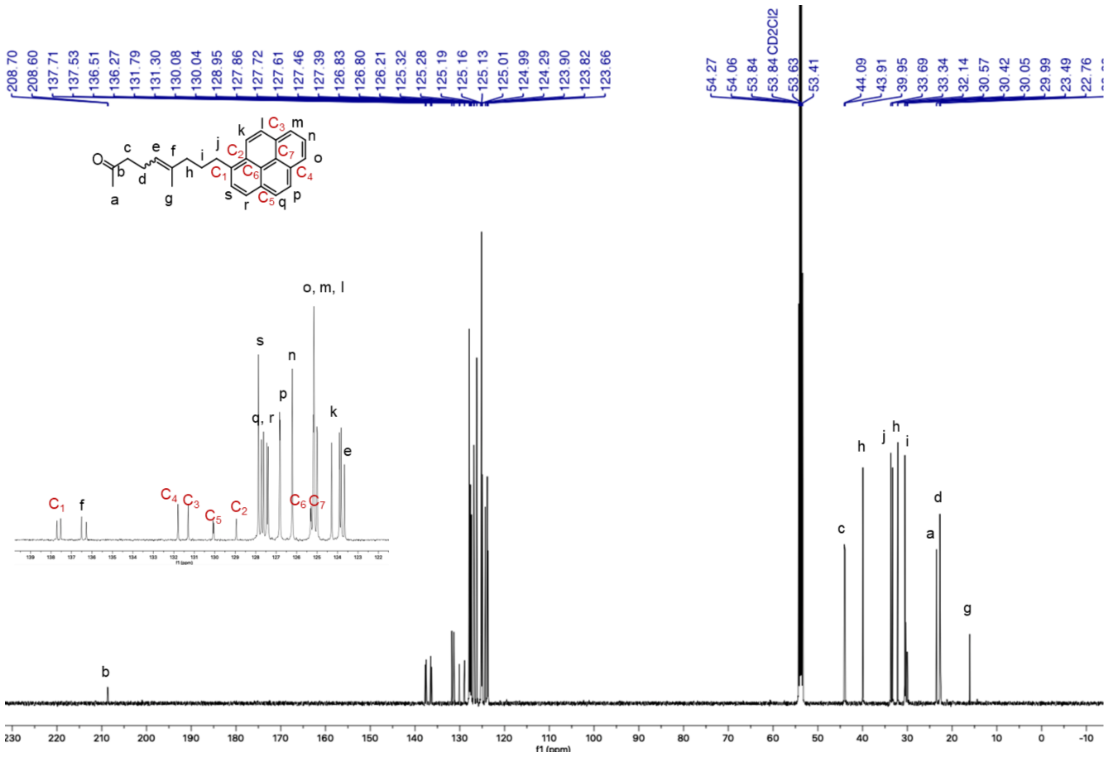

Fig. S20 126 MHz ^{13}C NMR spectrum of **16** in CD_2Cl_2.

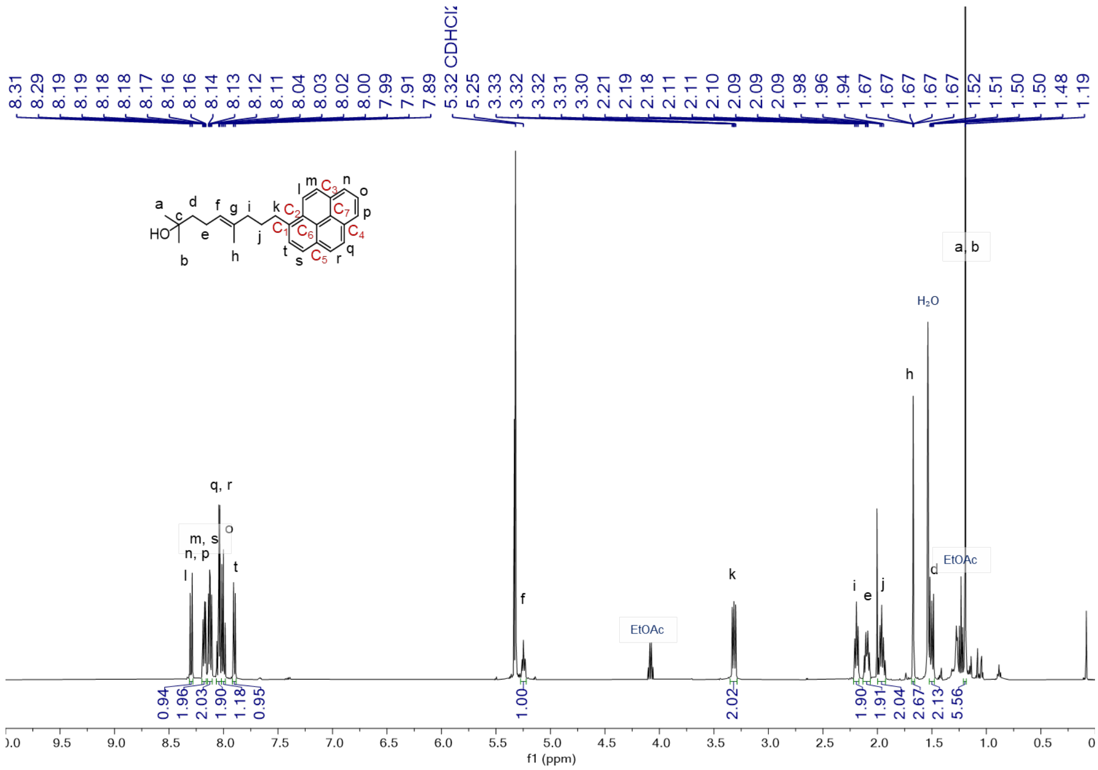

Fig. S21 500 MHz ^1H NMR spectrum of ***E*-17** in CD_2Cl_2.

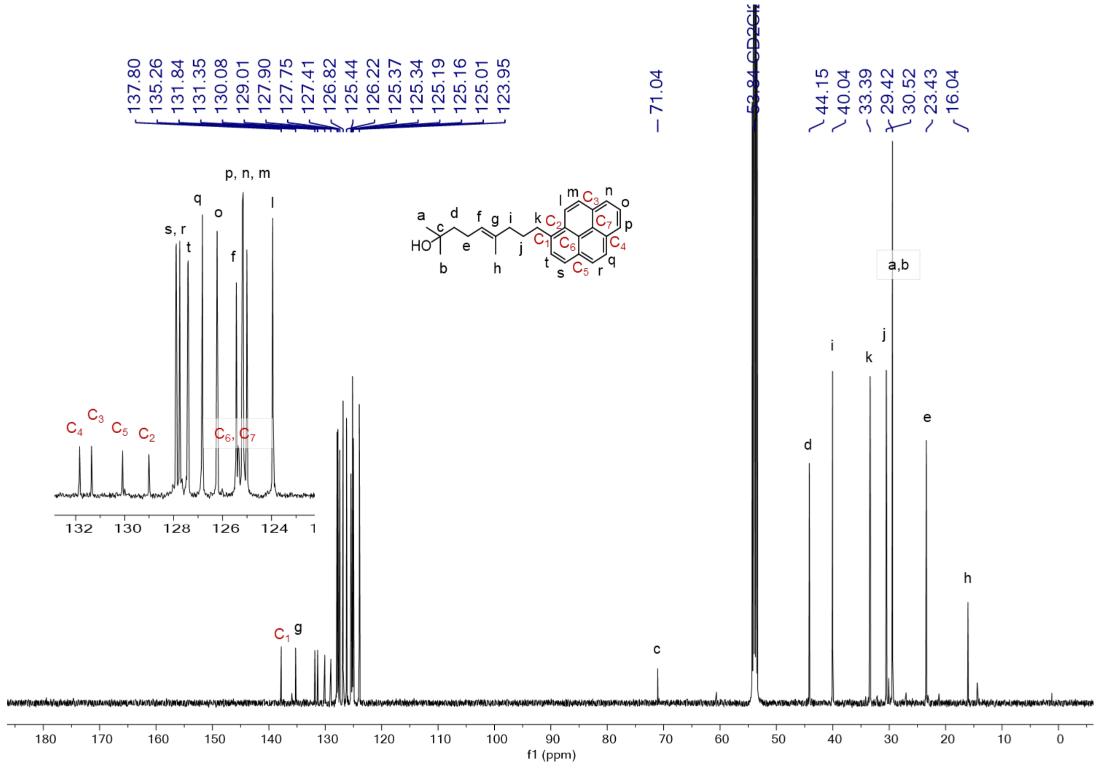

Fig. S22 126 MHz ^{13}C NMR spectrum of ***E*-17** in CD_2Cl_2.

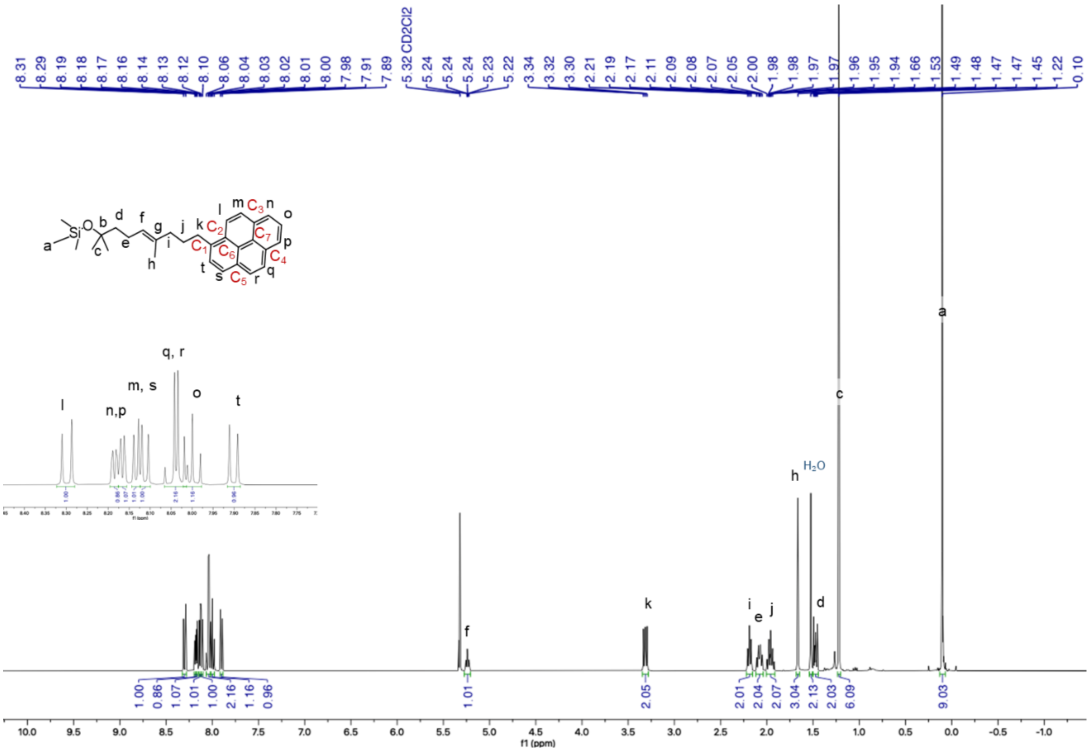

Fig, S23 500 MHz ^{1}H NMR spectrum of **18** in CD_2Cl_2.

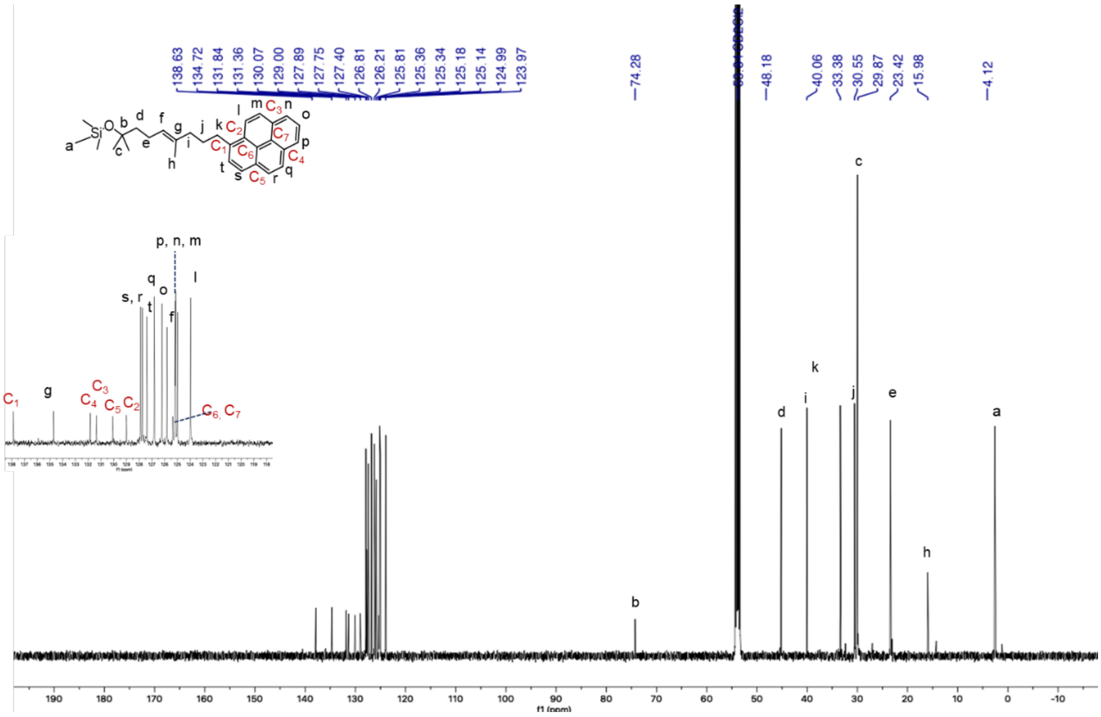

Fig. S24 126 MHz ^{13}C NMR spectrum of **18** in CD_2Cl_2.

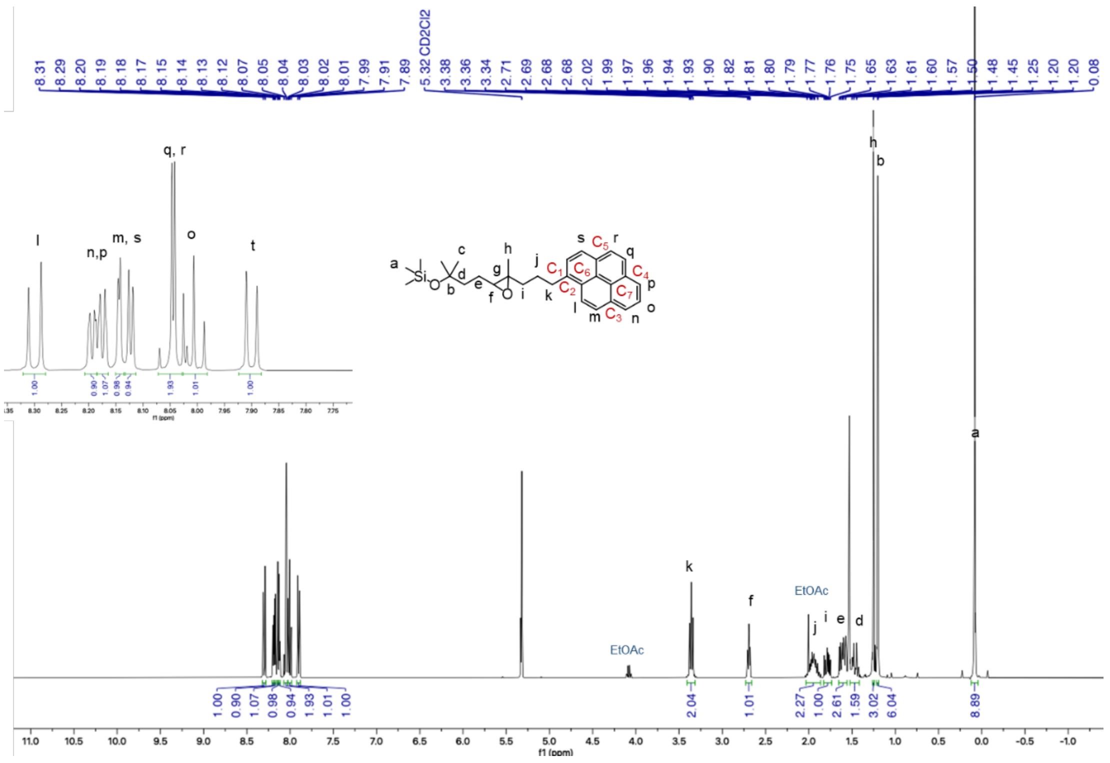

Fig. S25 500 MHz ^{1}H NMR spectrum of **19** in CD_2Cl_2.

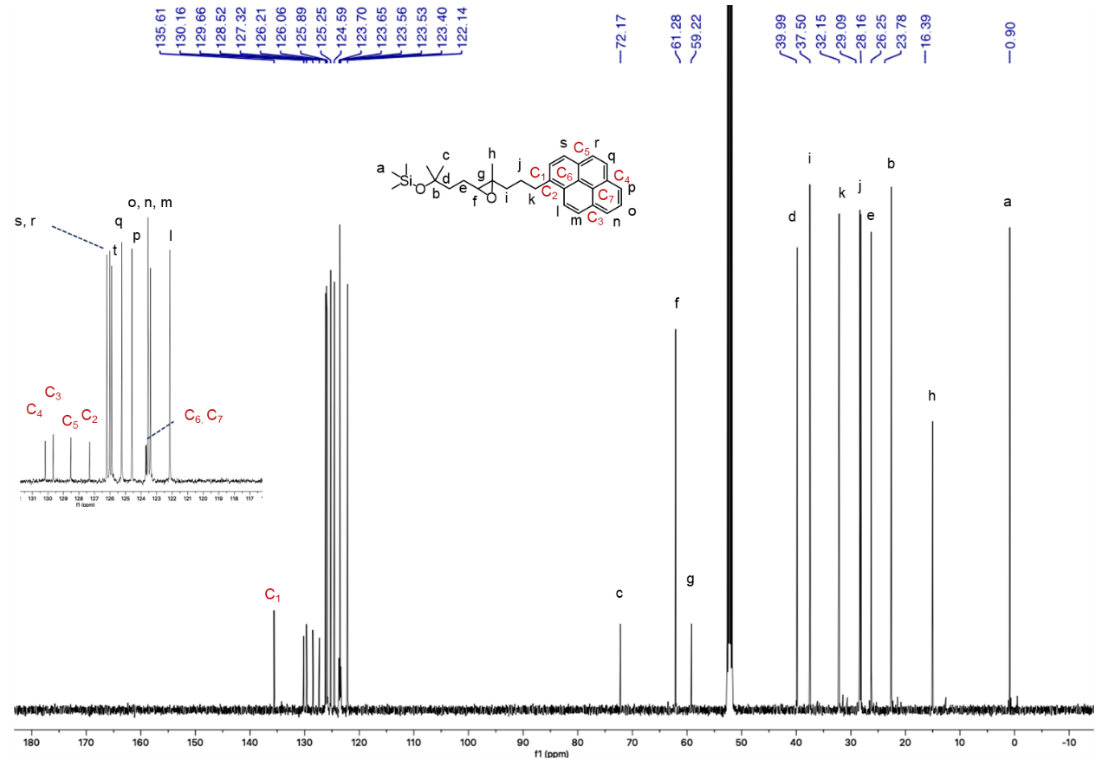

Fig. S26 126 MHz ^{13}C NMR spectrum of **19** in CD_2Cl_2.

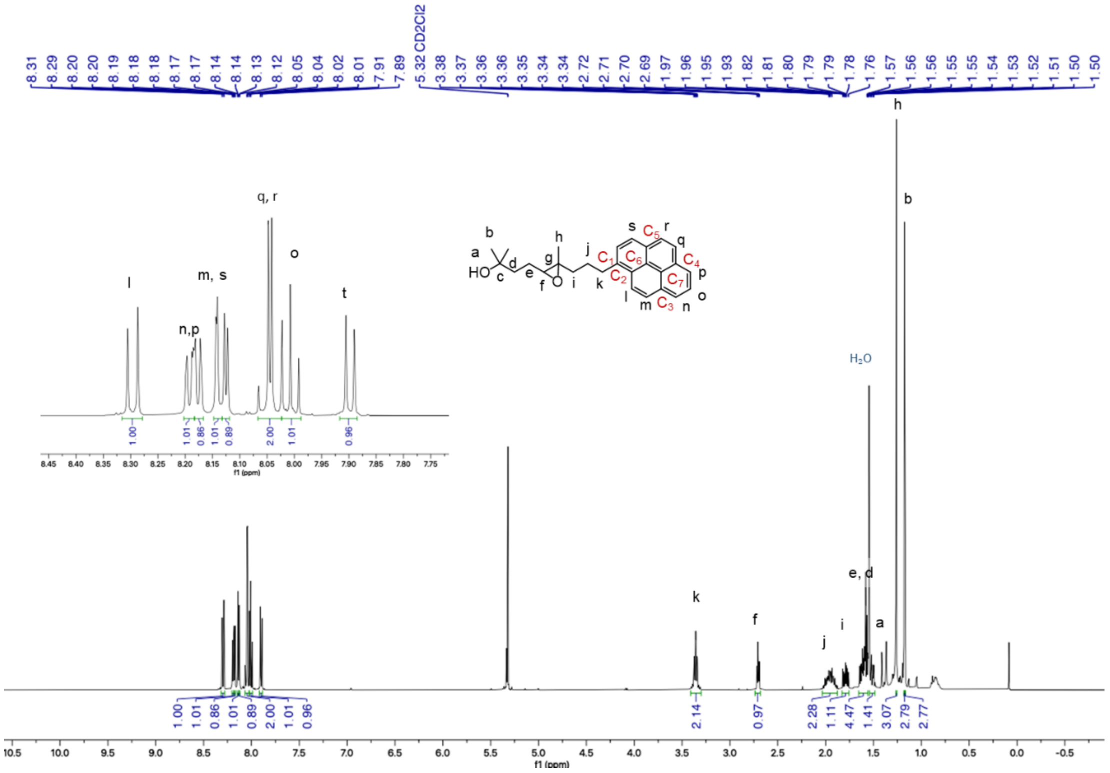

Fig. S27 500 MHz ^{1}H NMR spectrum of **3** in CD_2Cl_2

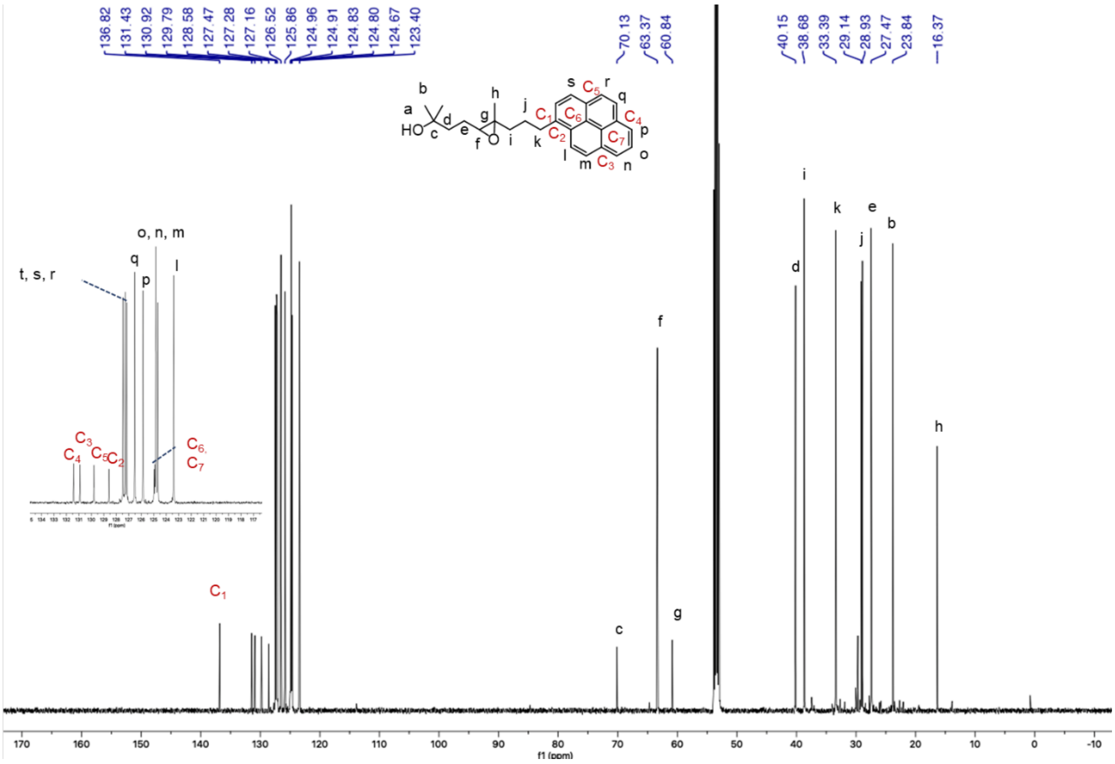

Fig. S28 126 MHz ^{13}C NMR spectrum of **3** in CD_2Cl_2.

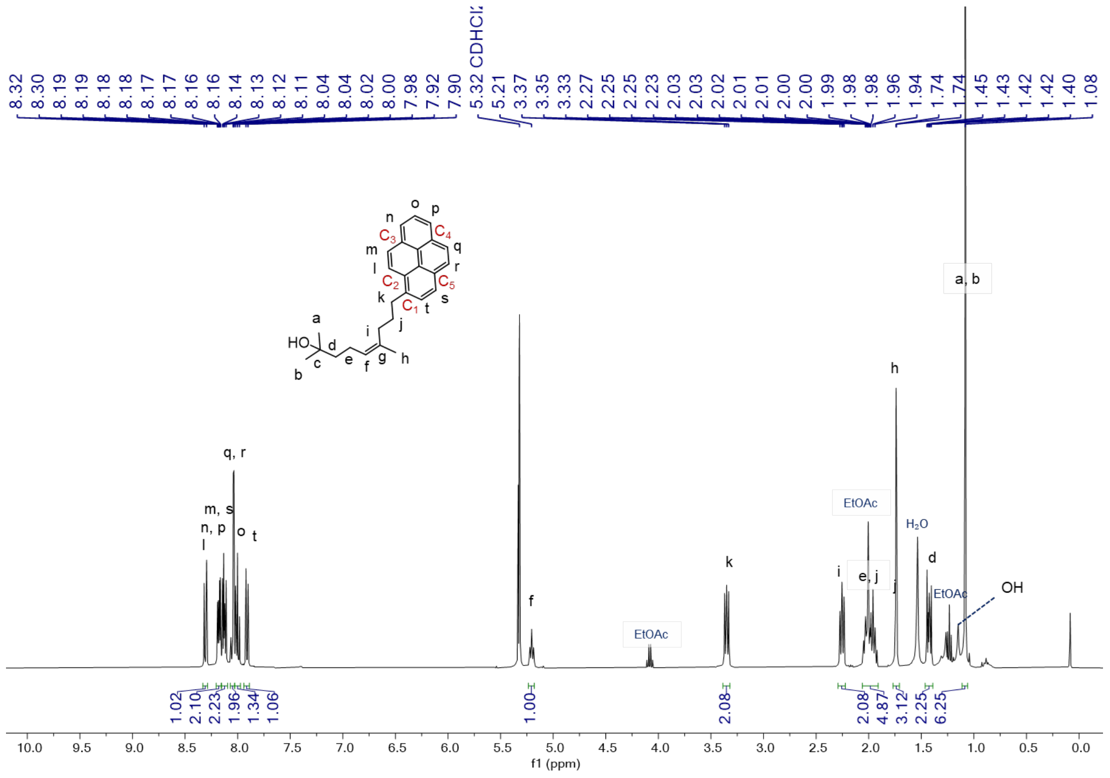

Fig. S29 500 MHz ^{1}H NMR spectrum of ***Z*-17** in CD_2Cl_2.

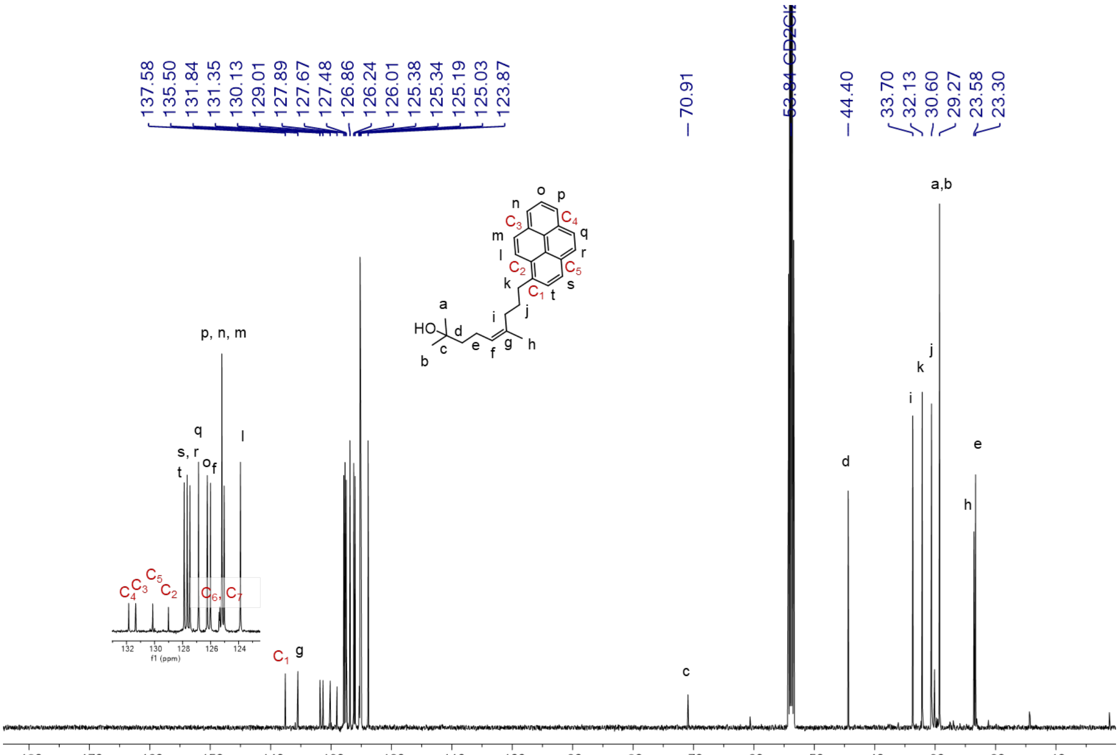

Fig. S30 126 MHz ^{13}C NMR spectrum of ***Z*-17** in CD_2Cl_2.

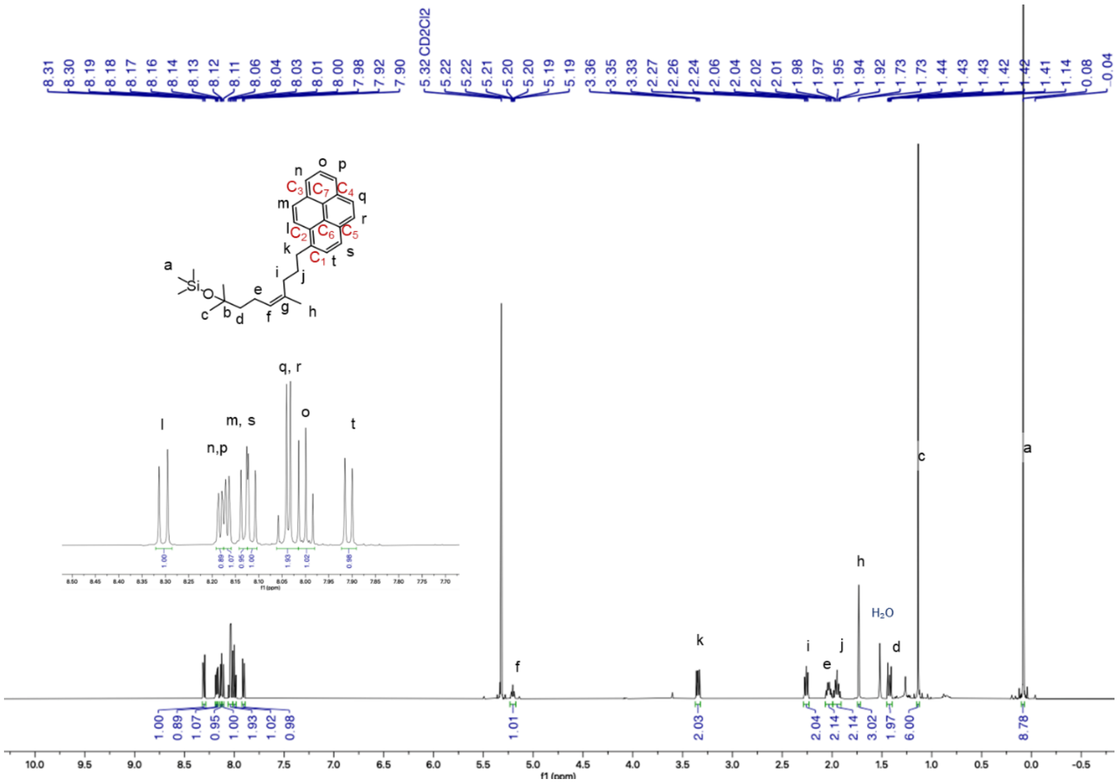

Fig. S31 500 MHz ^{1}H NMR spectrum of **21** in CD_2Cl_2.

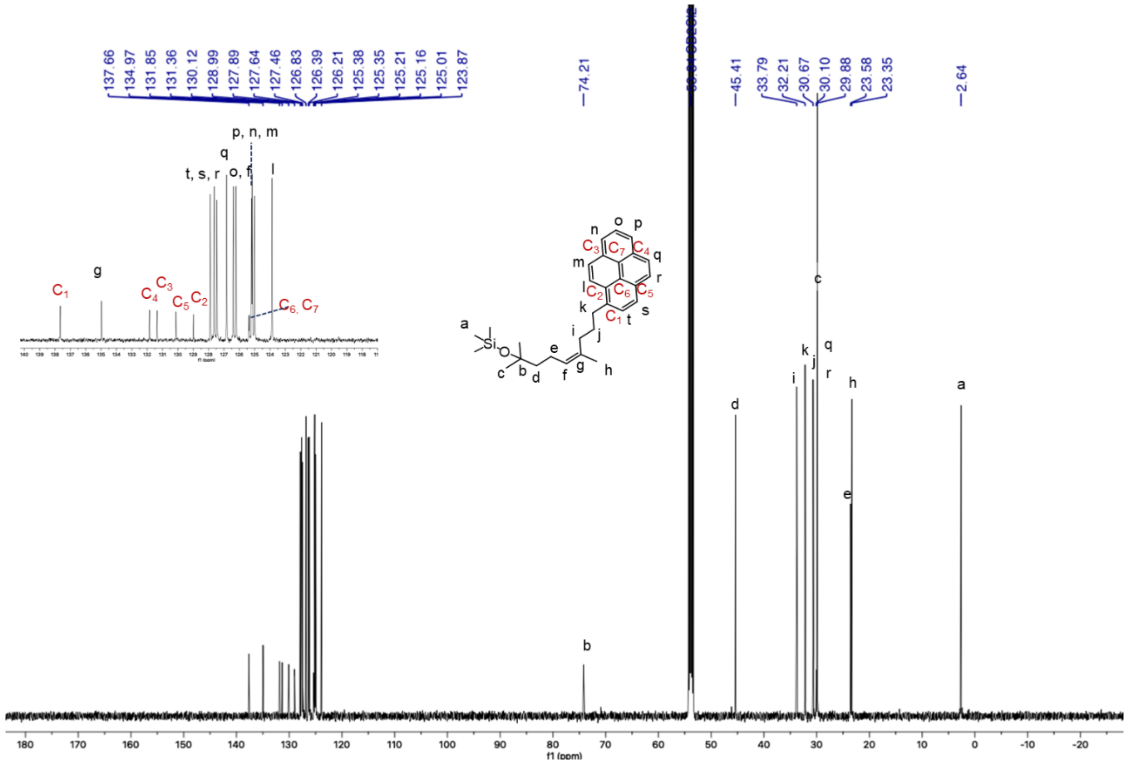

Fig. S32 126 MHz ^{13}C NMR spectrum of **21** in CD_2Cl_2.

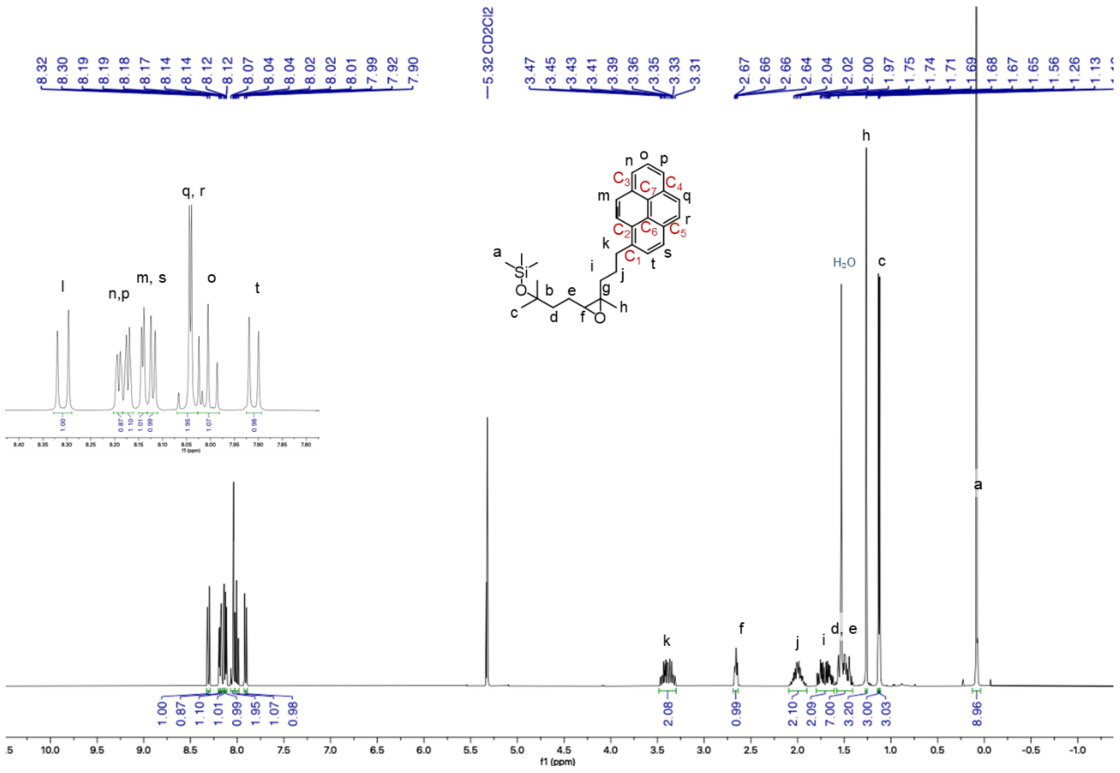

Fig. S33 500 MHz ^{1}H NMR spectrum of **22** in CD_2Cl_2.

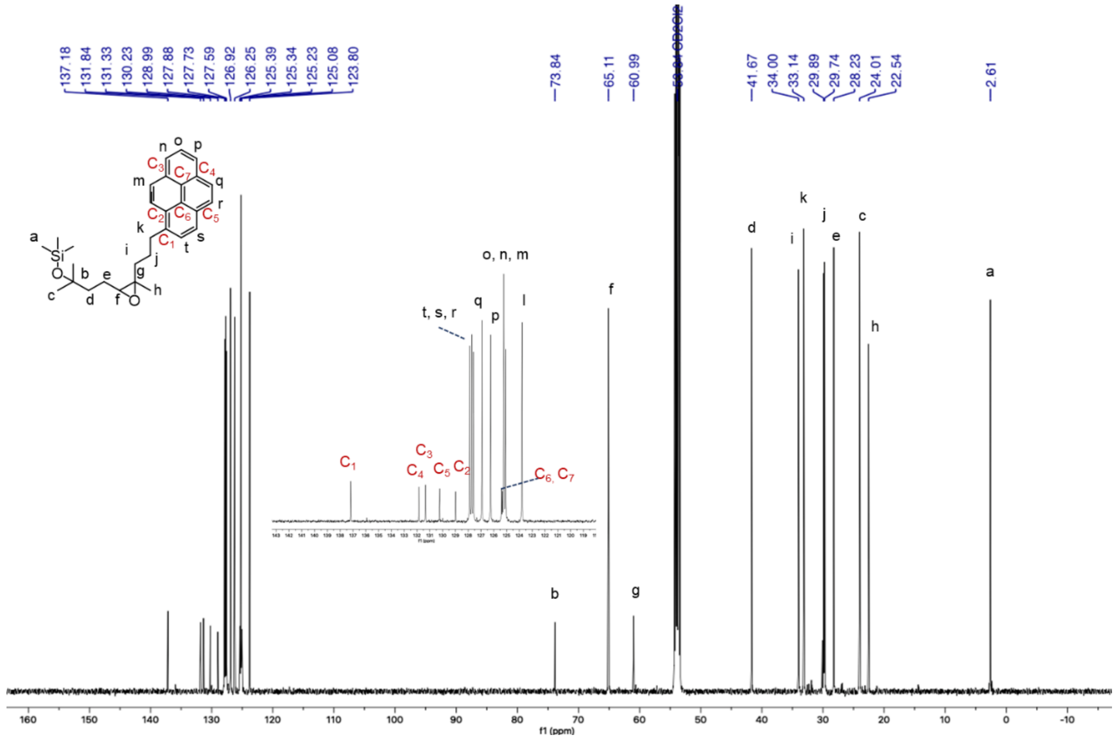

Fig. S34 126 MHz ^{13}C NMR spectrum of **22** in CD_2Cl_2.

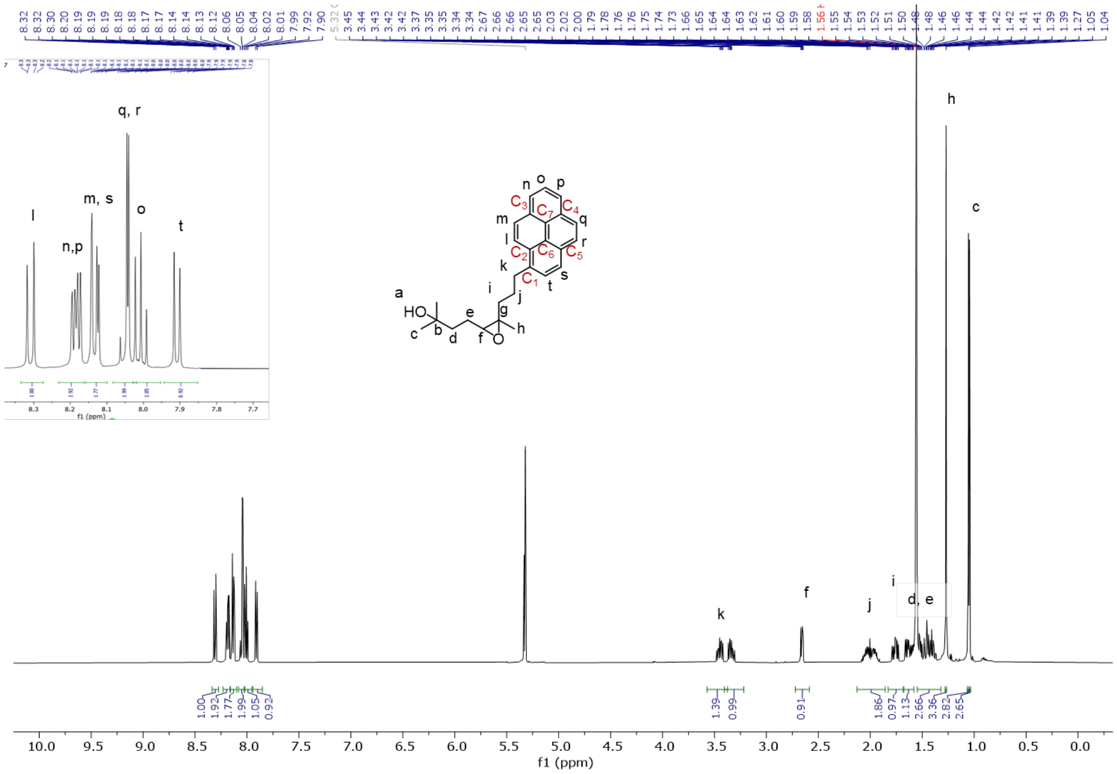

Fig. S35 500 MHz ^{1}H NMR spectrum of **4** in CD_2Cl_2.

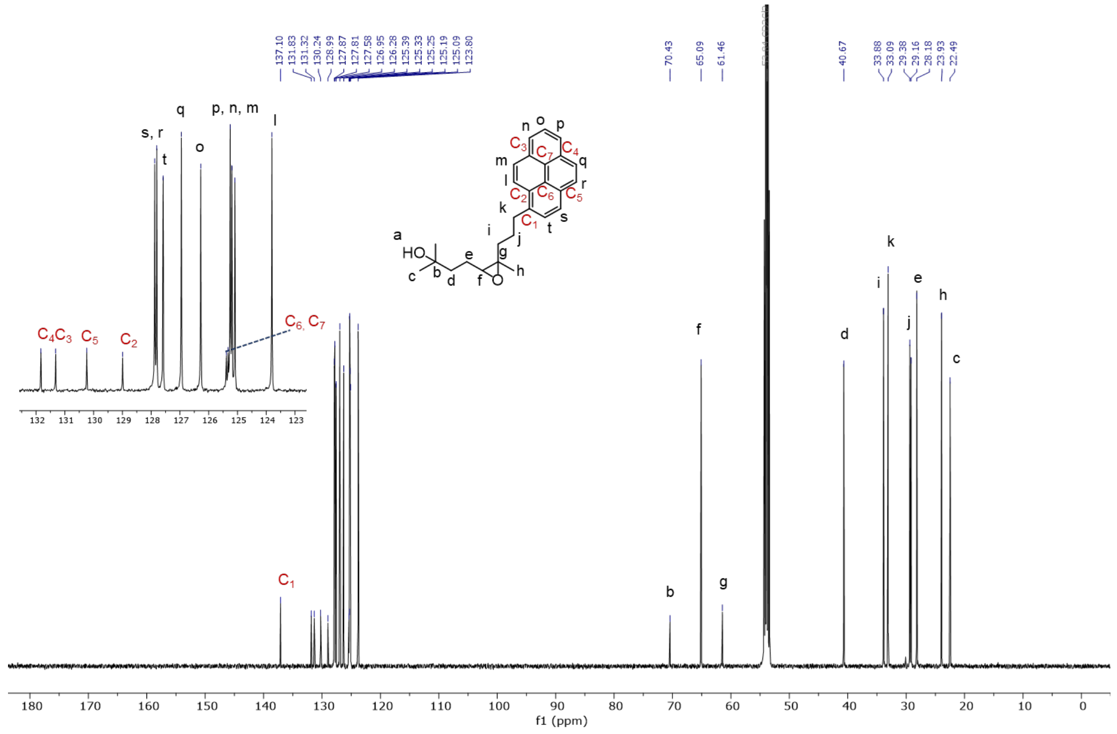

Fig. S36 126 MHz ^{13}C NMR spectrum of **4** in CD_2Cl_2.

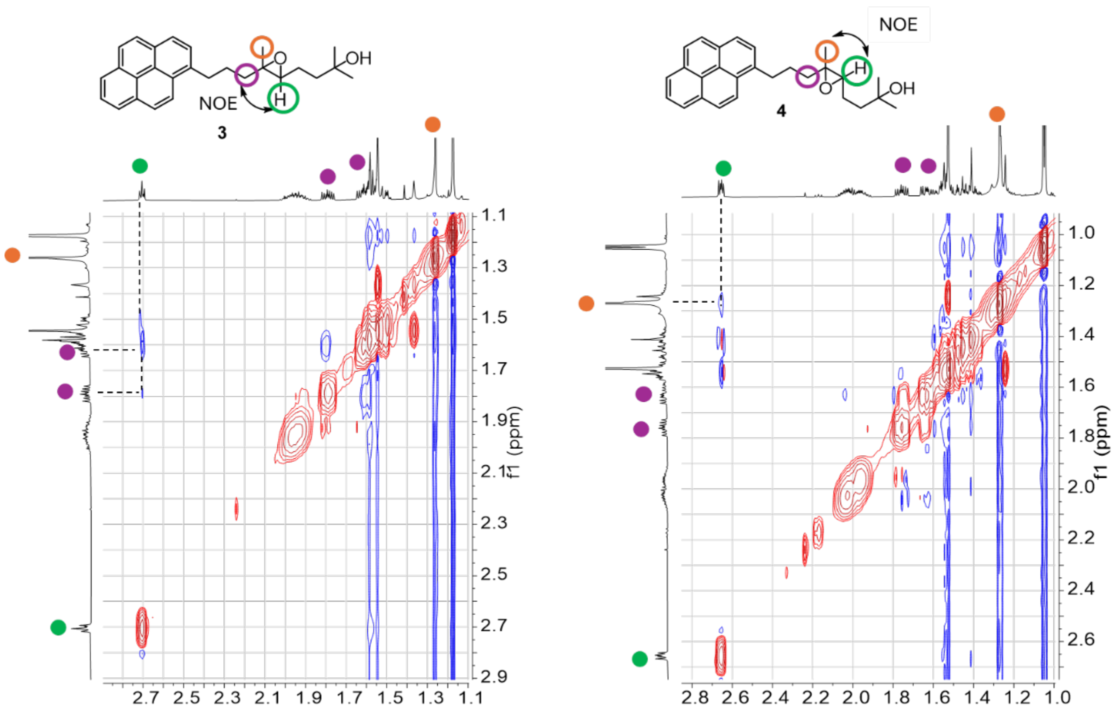

Fig. S37 NOESY spectra (500 MHz, CD_2Cl_2) of **3** (left) and **4** (right). The configurations of epoxide substituents were confirmed by observed NOE cross-peaks.

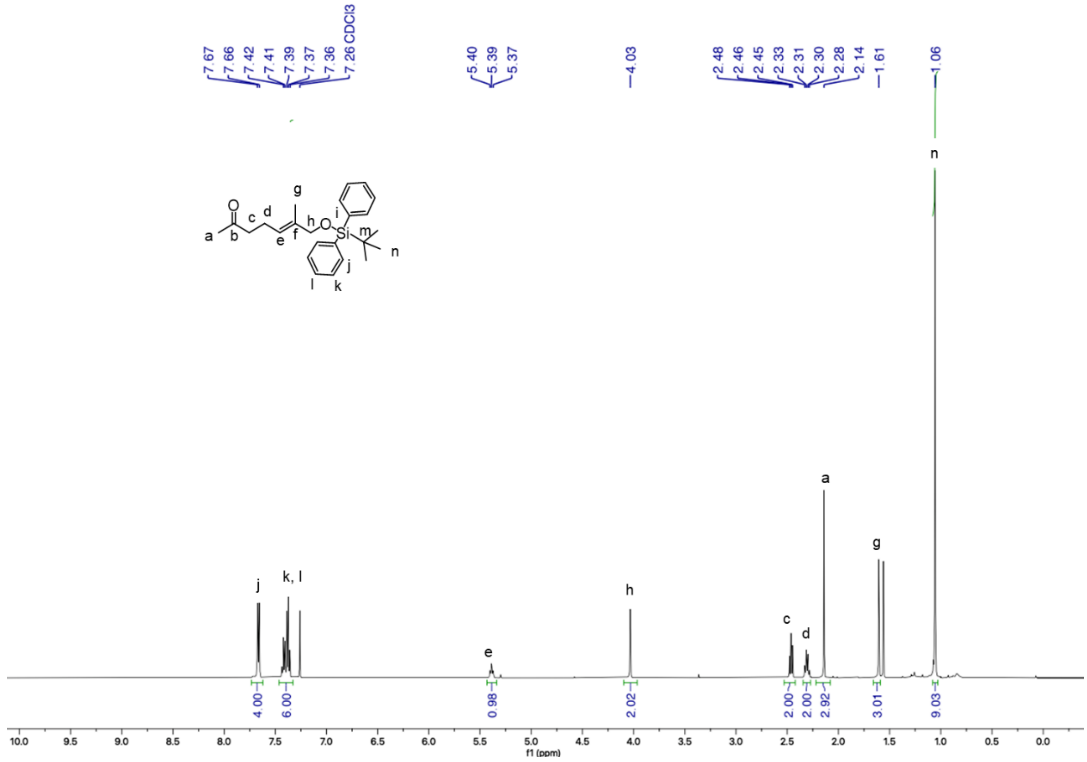

Fig. S38 500 MHz ^{1}H NMR spectrum of **25** in $CDCl_3$.

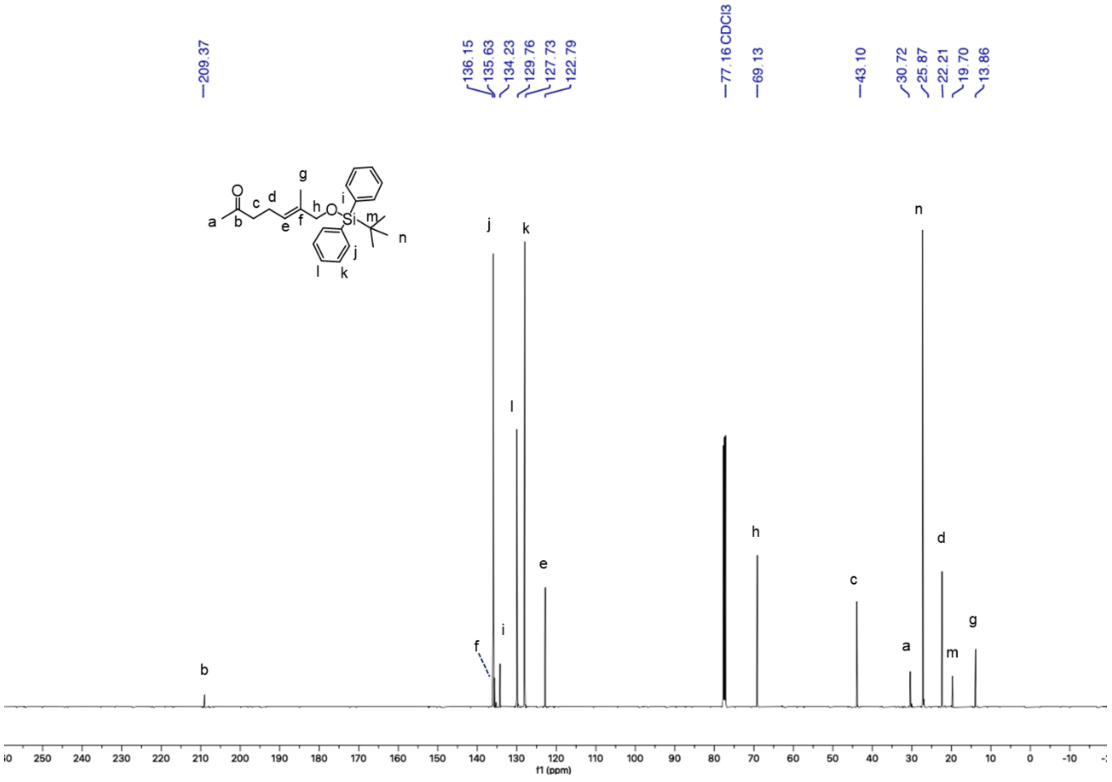

Fig. S39 126 MHz ^{13}C NMR spectrum of **25** in $CDCl_3$.

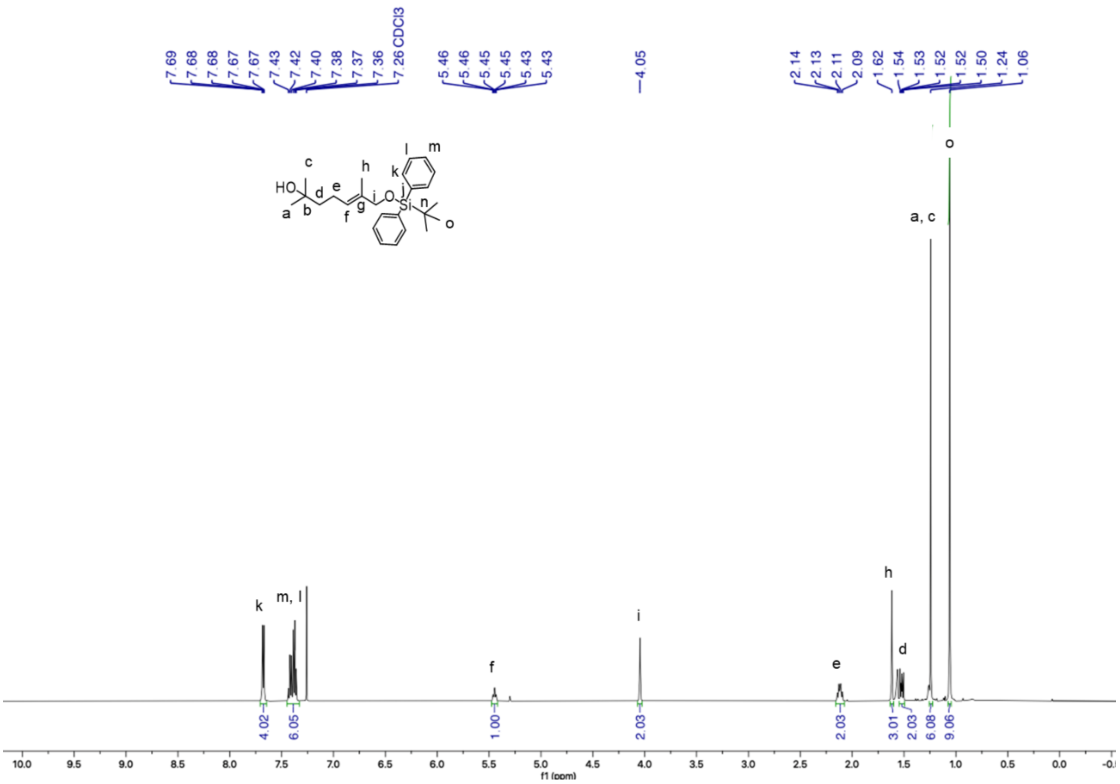

Fig. S40 500 MHz ^{1}H NMR spectrum of **26** in $CDCl_3$.

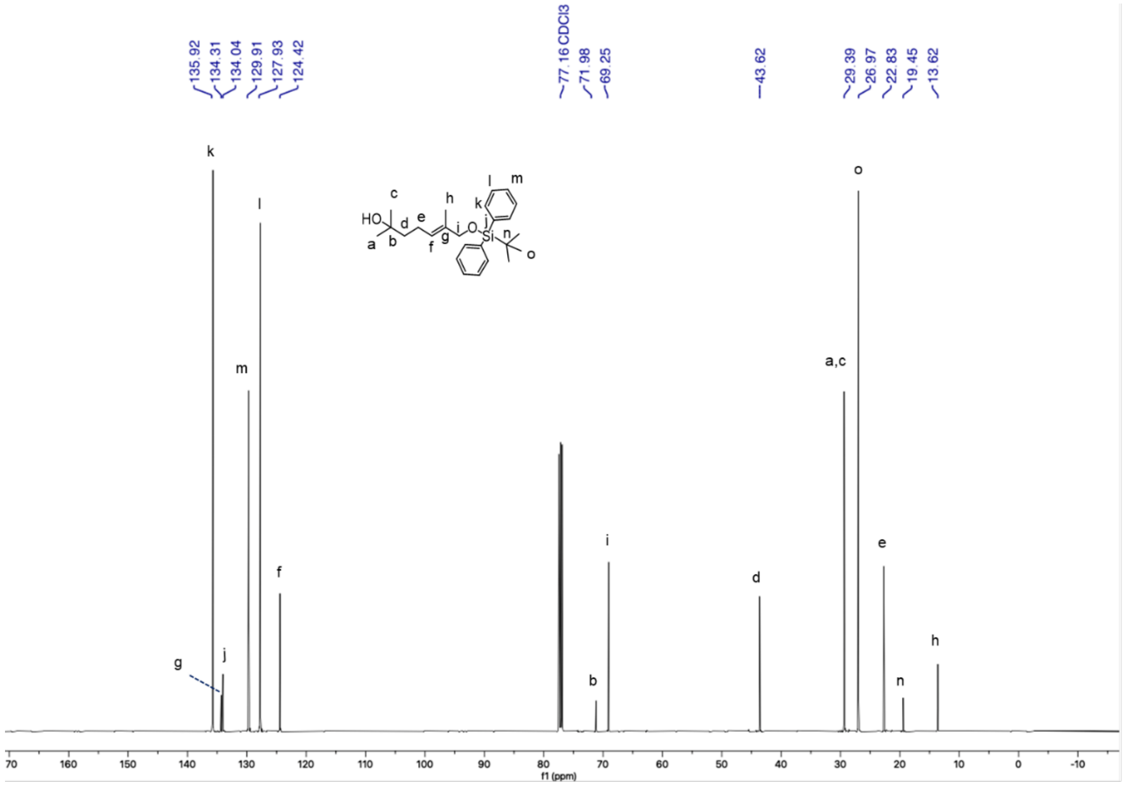

Fig. S41 126 MHz ^{13}C NMR spectrum of **26** in $CDCl_3$.

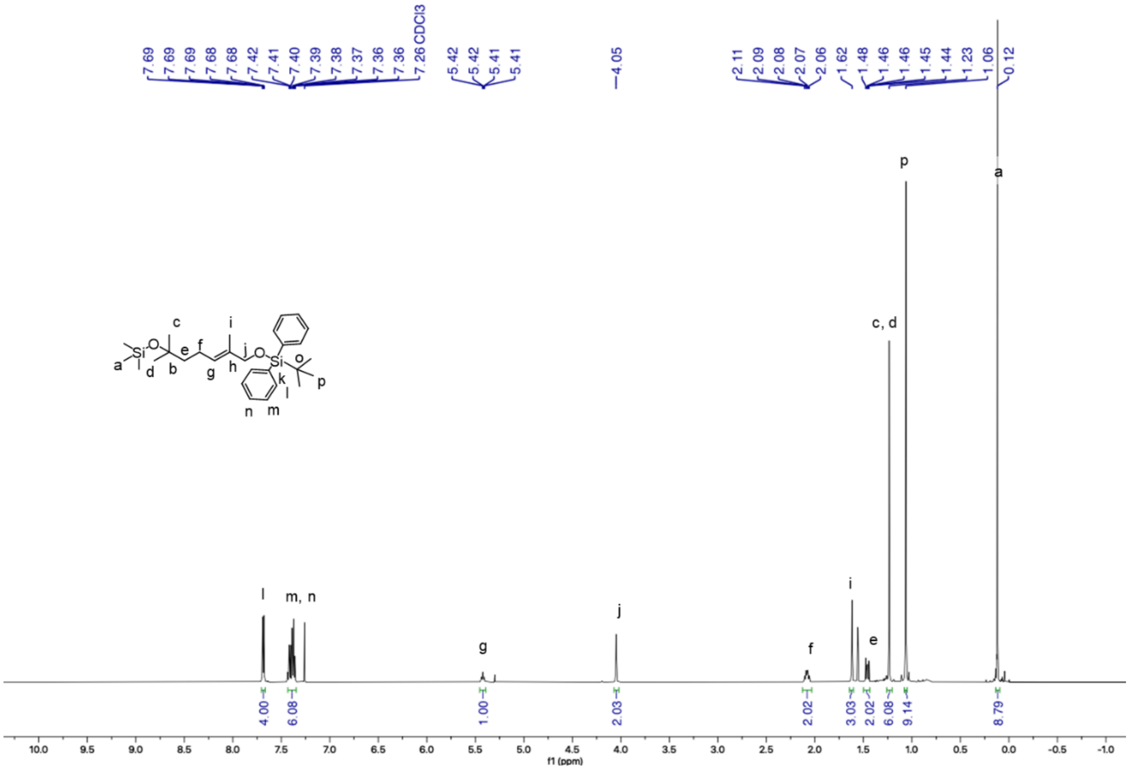

Fig. S42 500 MHz ^{1}H NMR spectrum of **27** in $CDCl_3$.

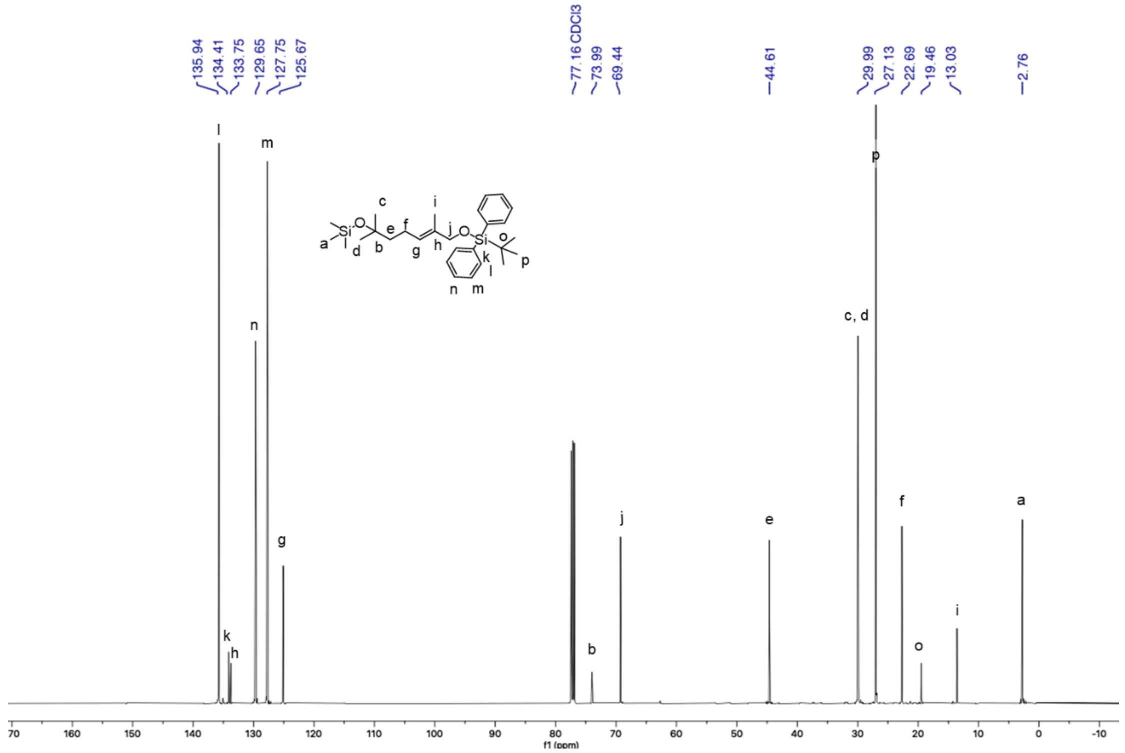

Fig. S43 126 MHz ^{13}C NMR spectrum of **27** in $CDCl_3$.

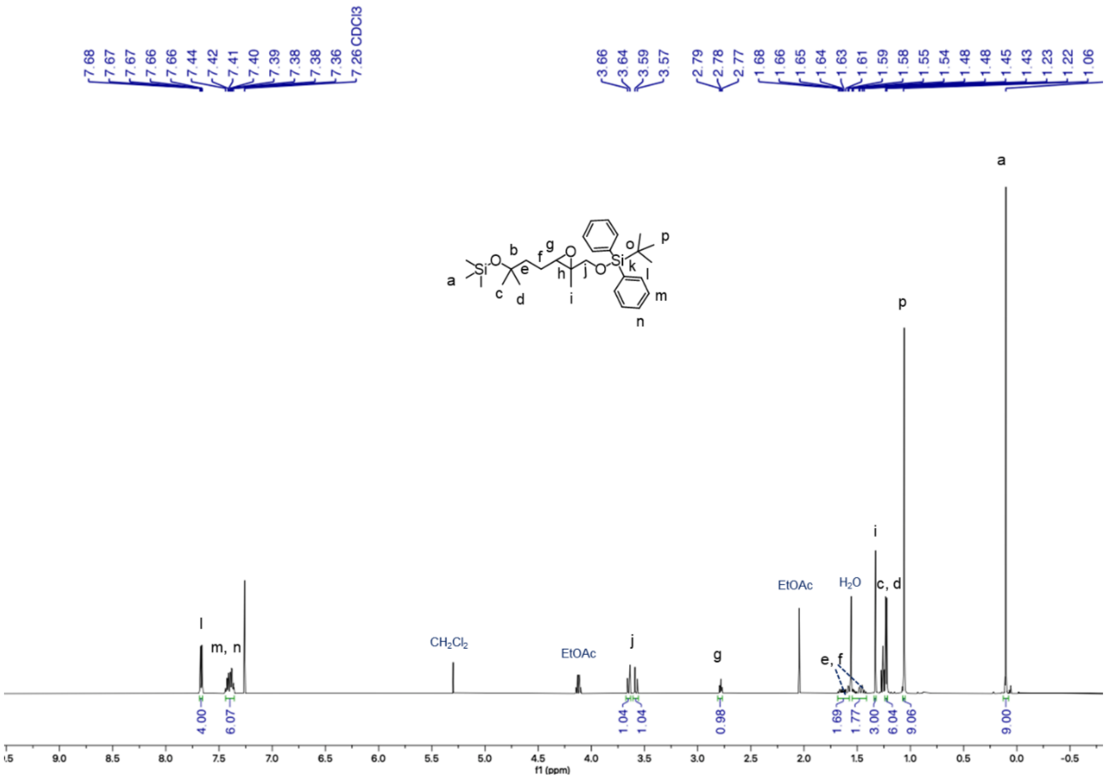

Fig. S44 500 MHz ^{1}H NMR spectrum of **28** in $CDCl_3$.

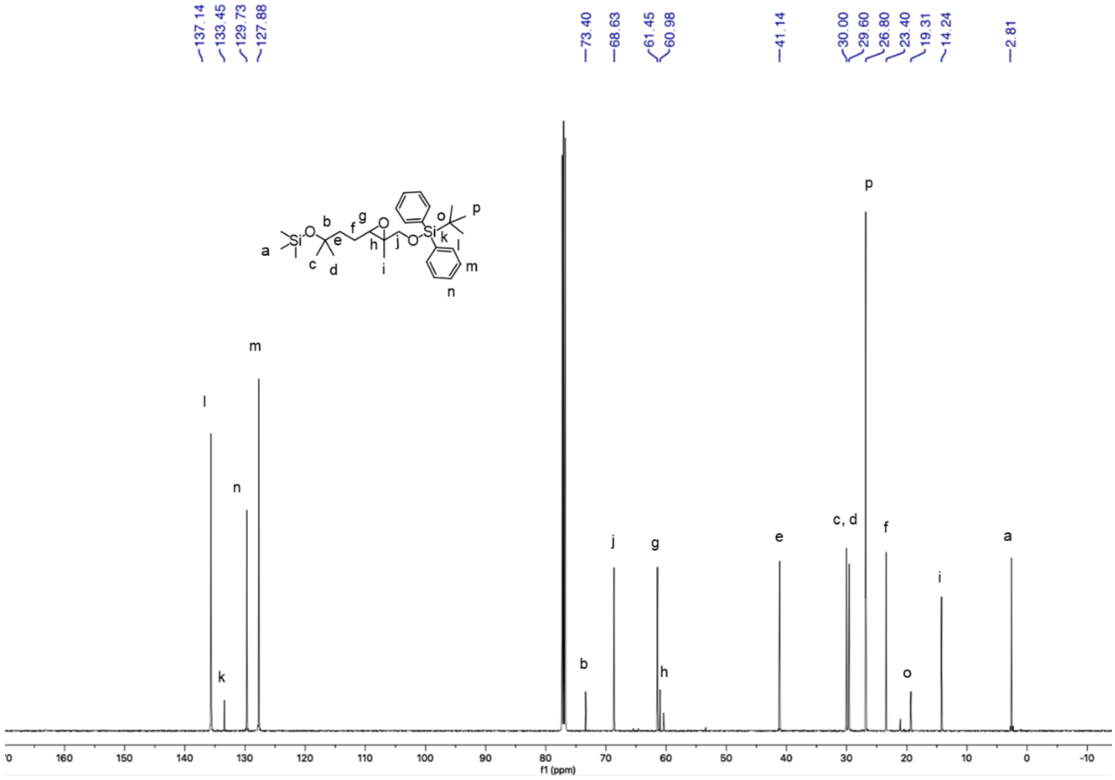

Fig. S45 126 MHz ^{13}C NMR spectrum of **28** in $CDCl_3$.

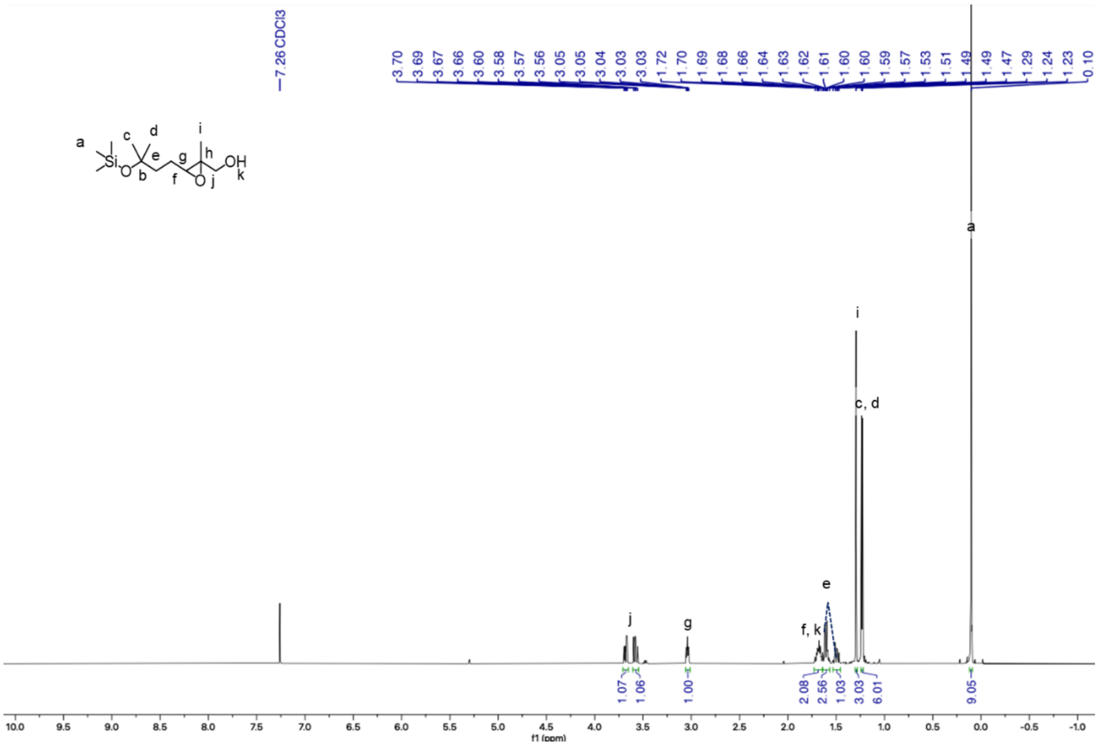

Fig. S46 500 MHz ^{1}H NMR spectrum of **29** in $CDCl_3$.

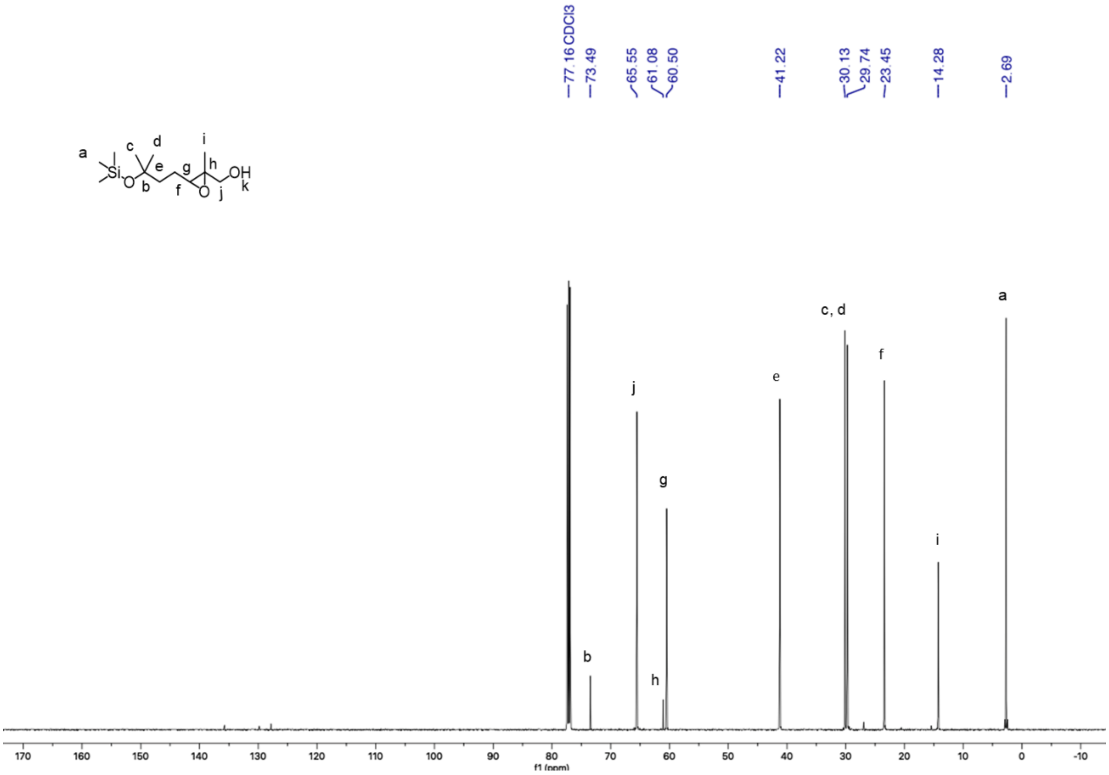

Fig. S47 126 MHz ^{13}C NMR spectrum of **29** in $CDCl_3$.

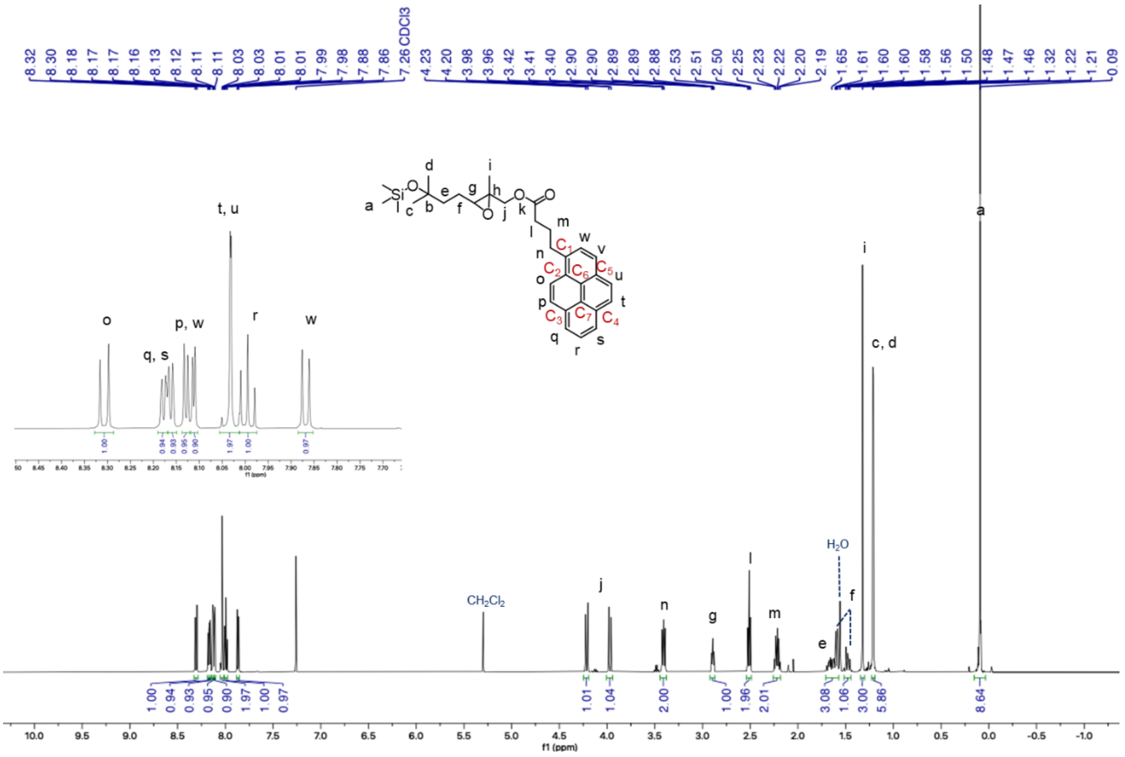

Fig. S48 500 MHz ^{1}H NMR spectrum of **30** in $CDCl_3$.

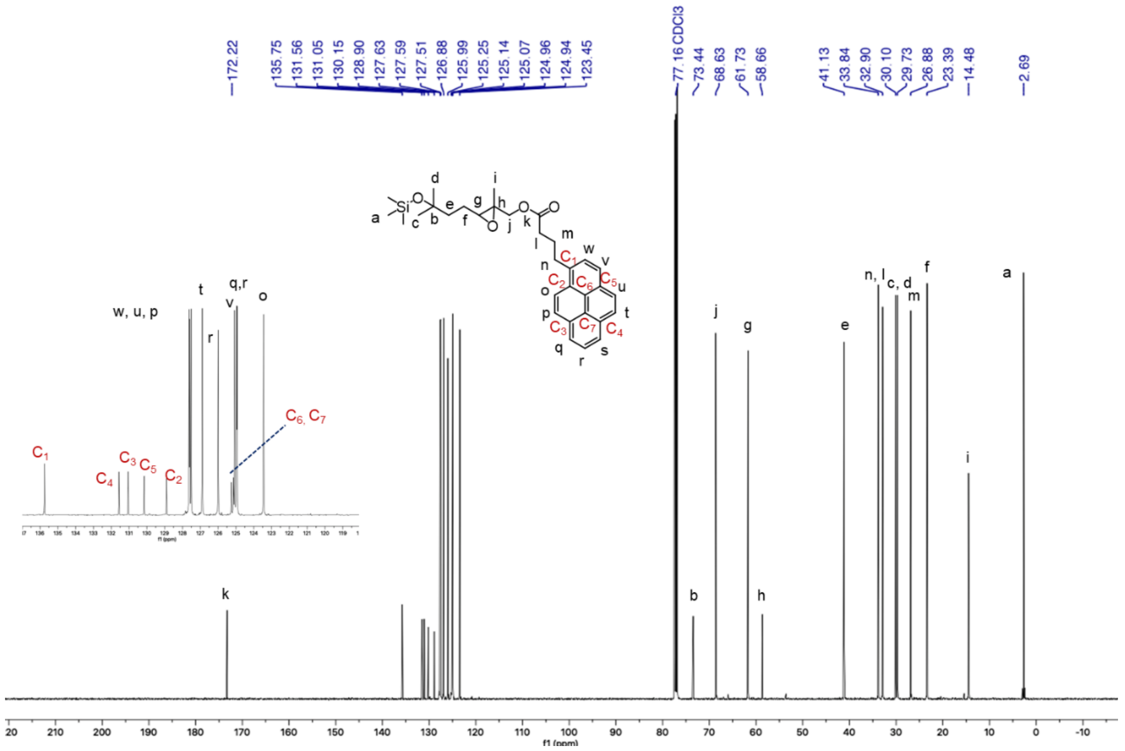

Fig. S49 126 MHz ^{13}C NMR spectrum of **30** in $CDCl_3$.

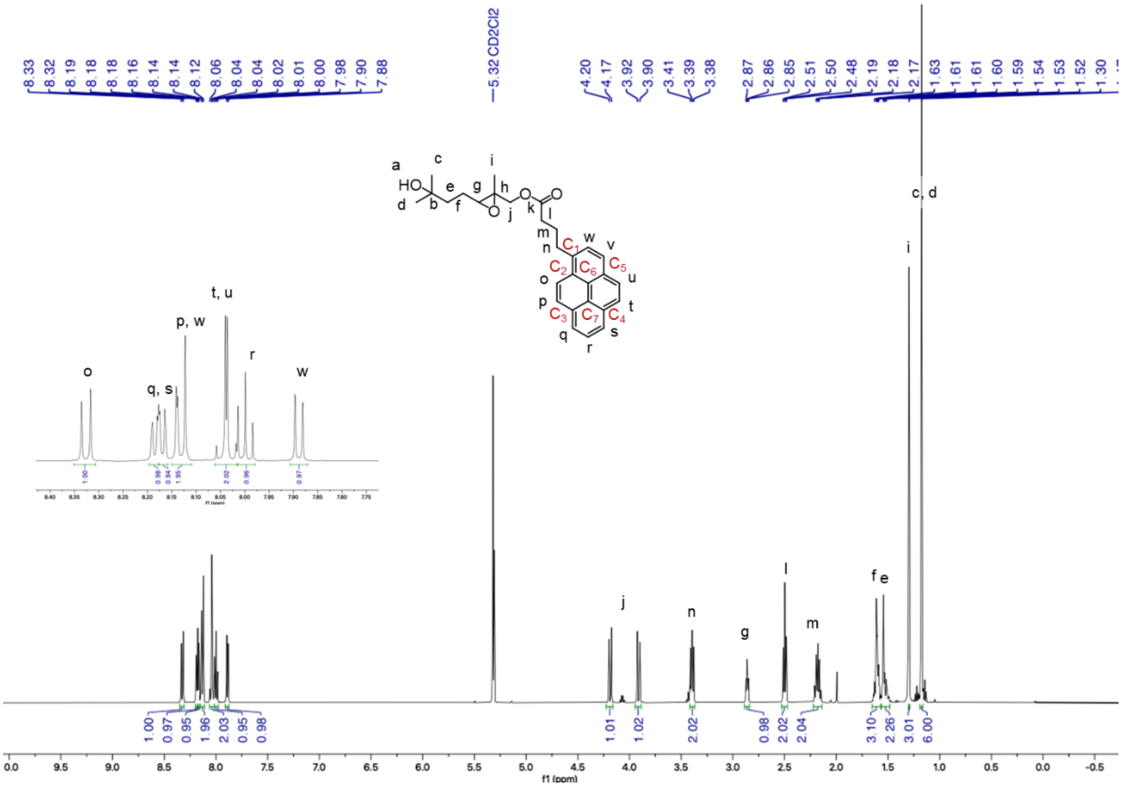

Fig. S50 500 MHz ^{1}H NMR spectrum of **2** in CD_2Cl_2.

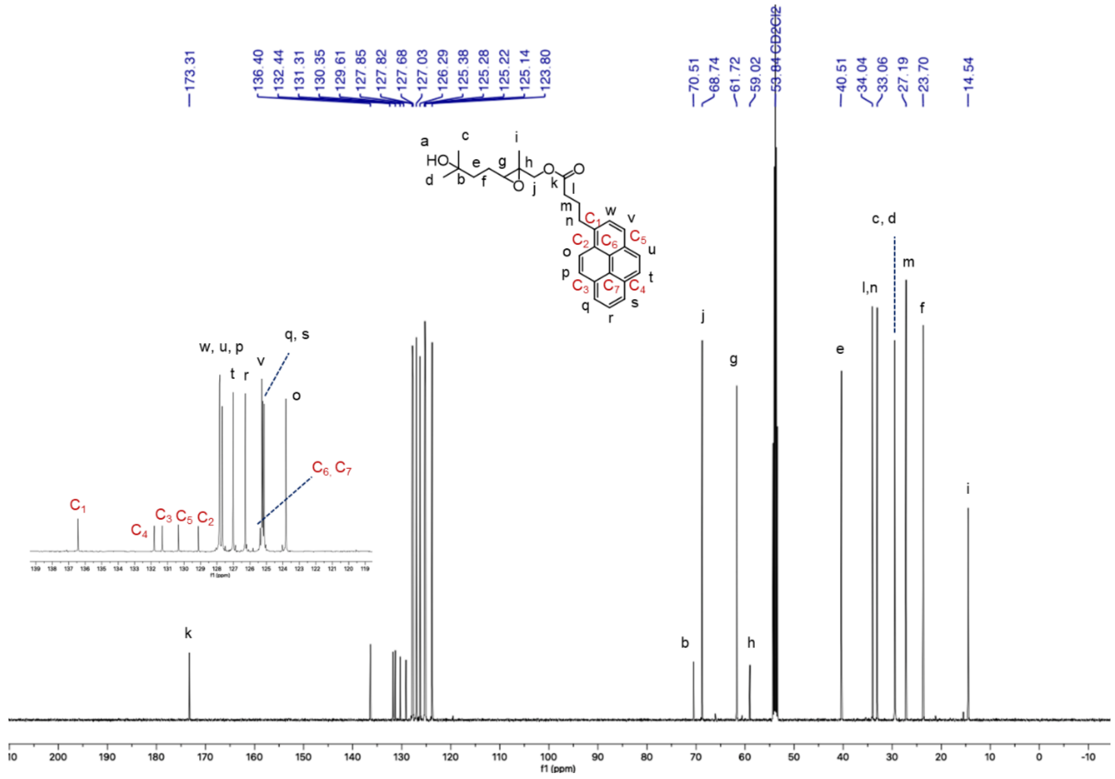

Fig. S51 126 MHz ^{13}C NMR spectrum of **2** in CD_2Cl_2.

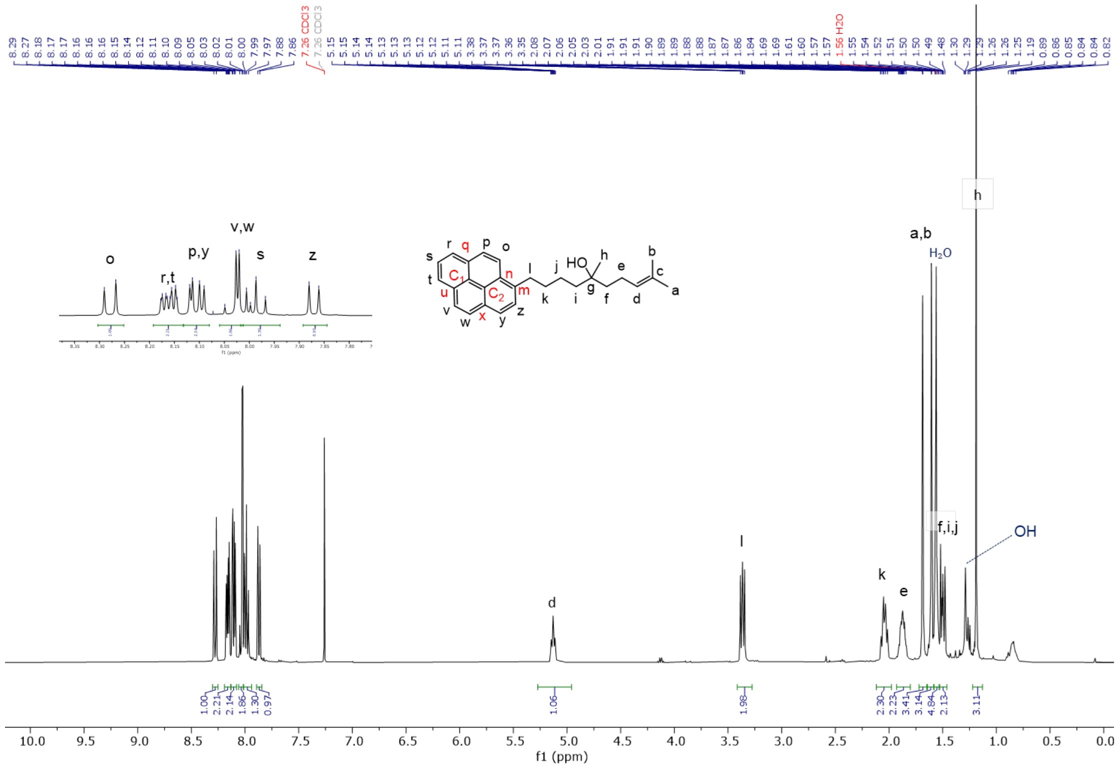

Fig. S52 500 MHz ^{1}H NMR spectrum of **31** in $CDCl_3$.

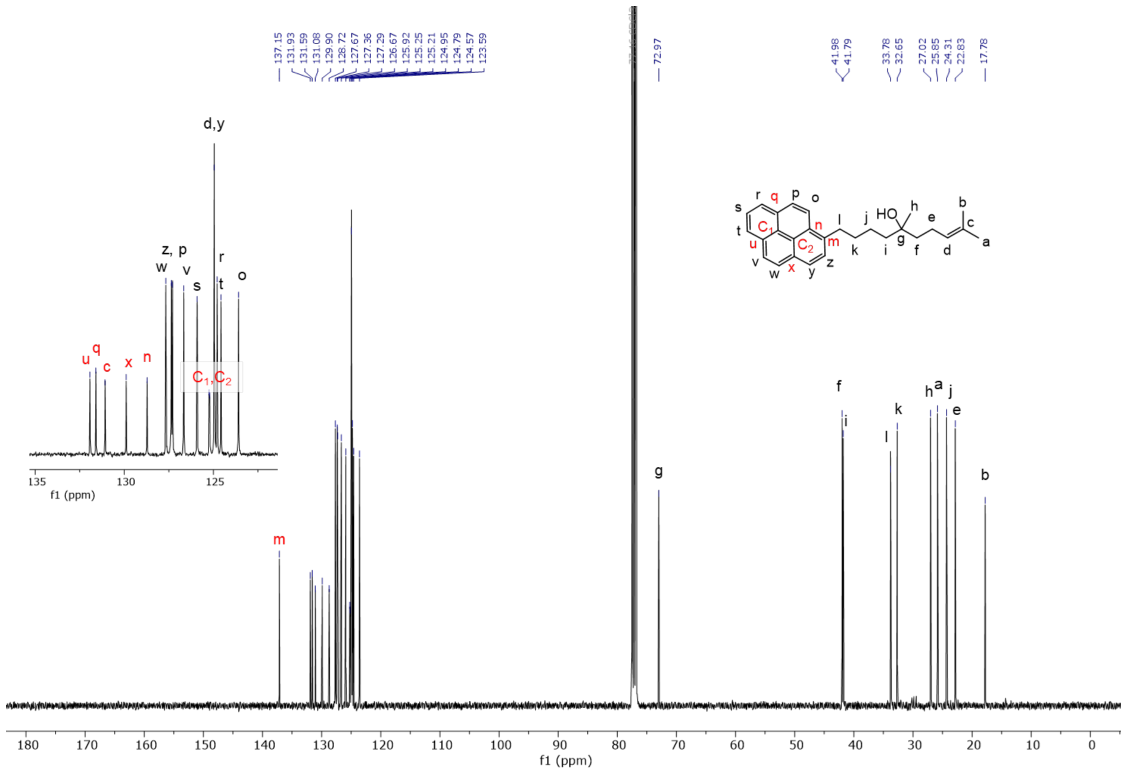

Fig. S53 126 MHz ^{13}C NMR spectrum of **31** in $CDCl_3$**.**

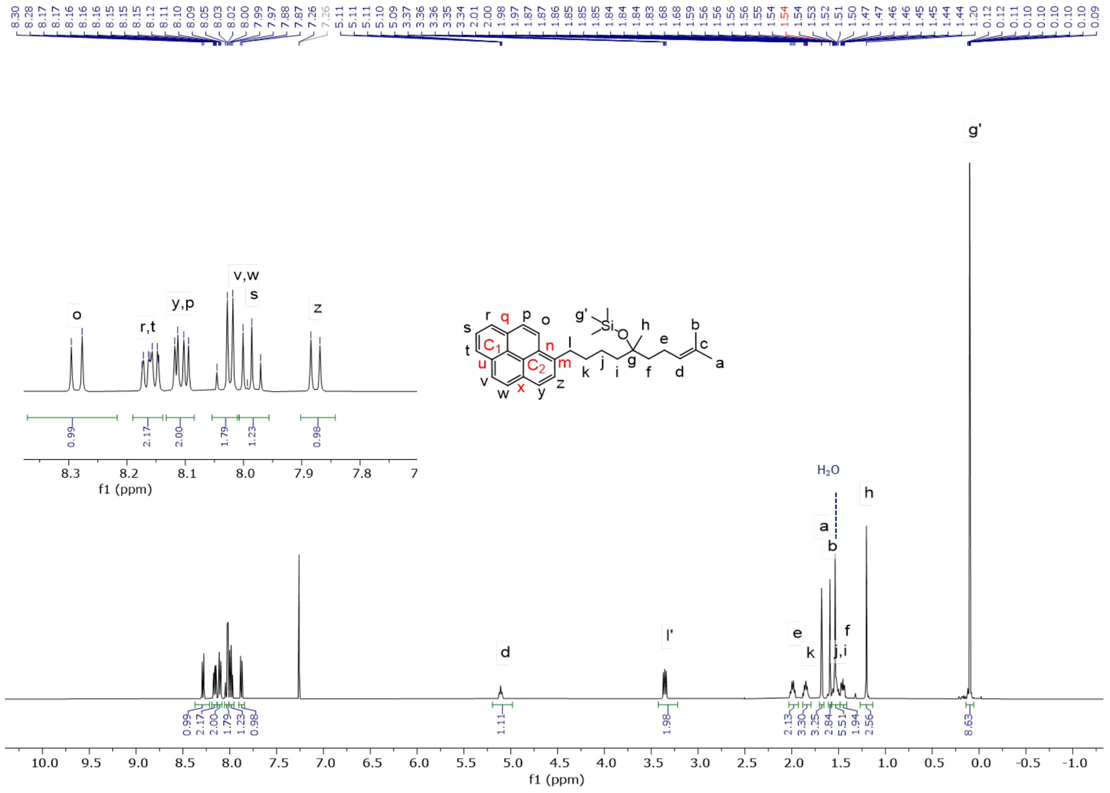

Fig. **S54** 500 MHz ^{1}H NMR spectrum of **32** in $CDCl_3$.

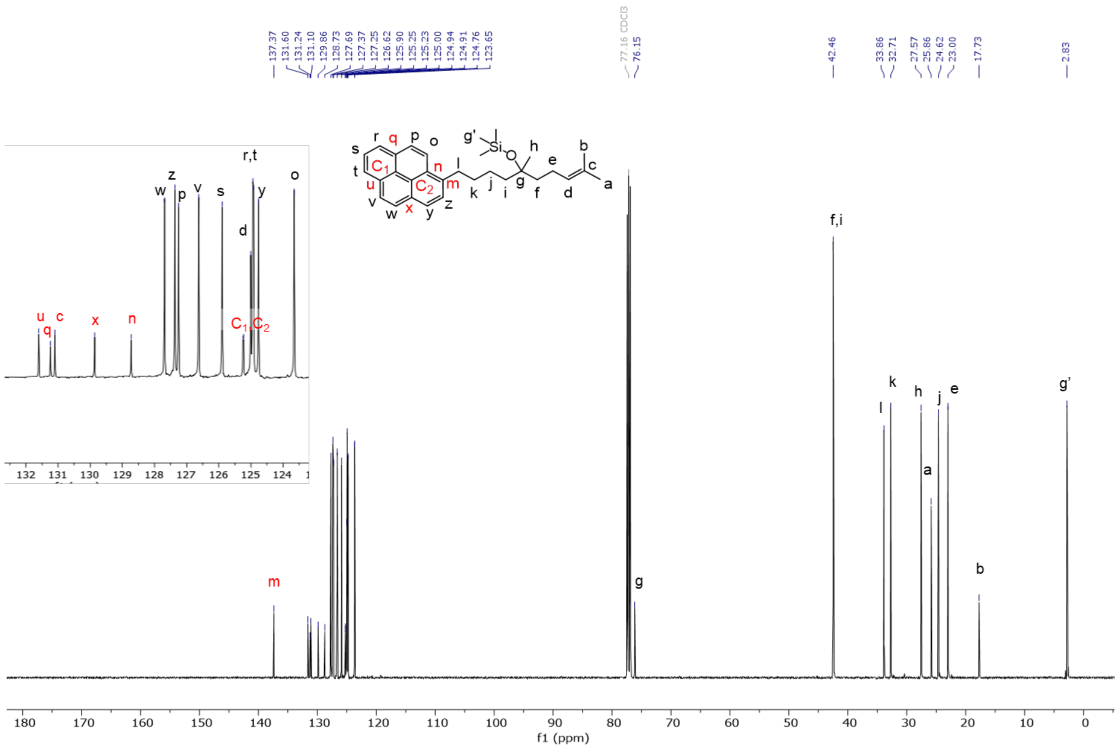

Fig. S55 126 MHz ^{13}C NMR spectrum of **32** in $CDCl_3$.

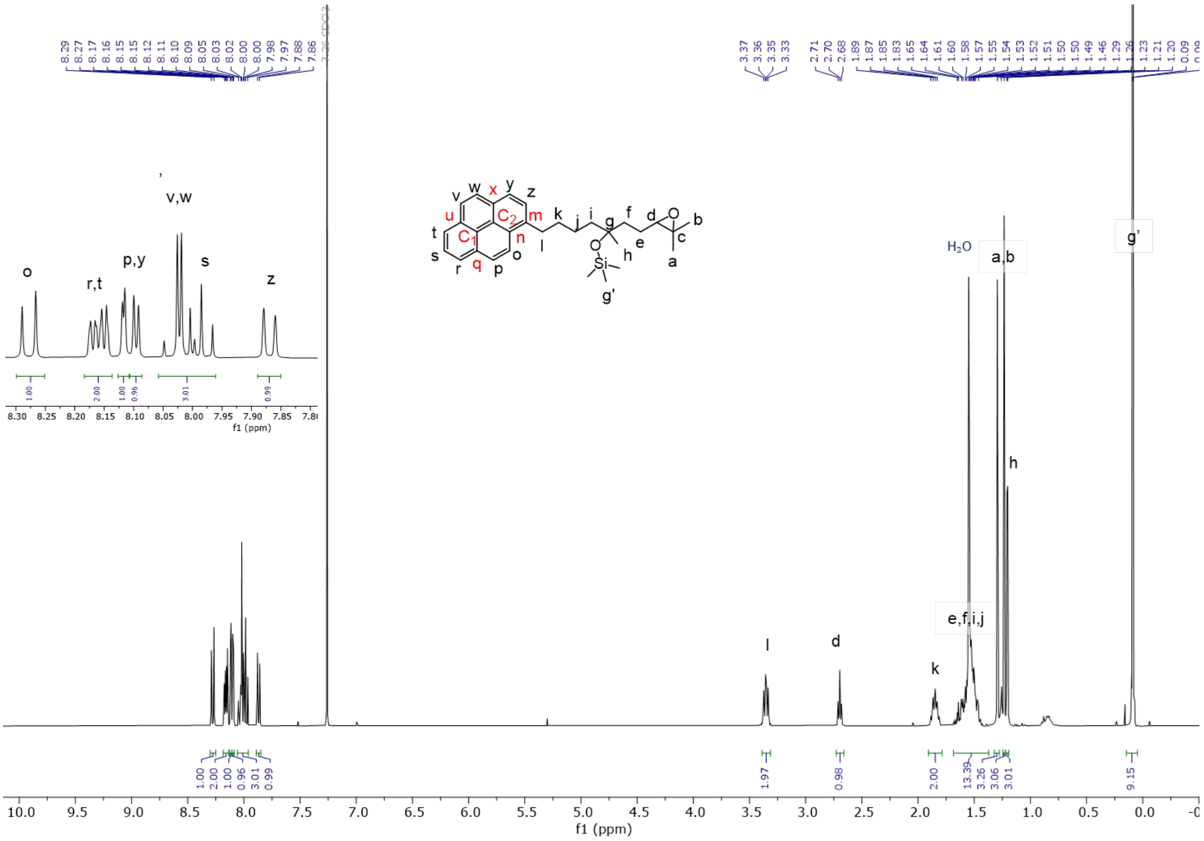

Fig. S56 500 MHz ^{1}H NMR spectrum of **33** in $CDCl_3$.

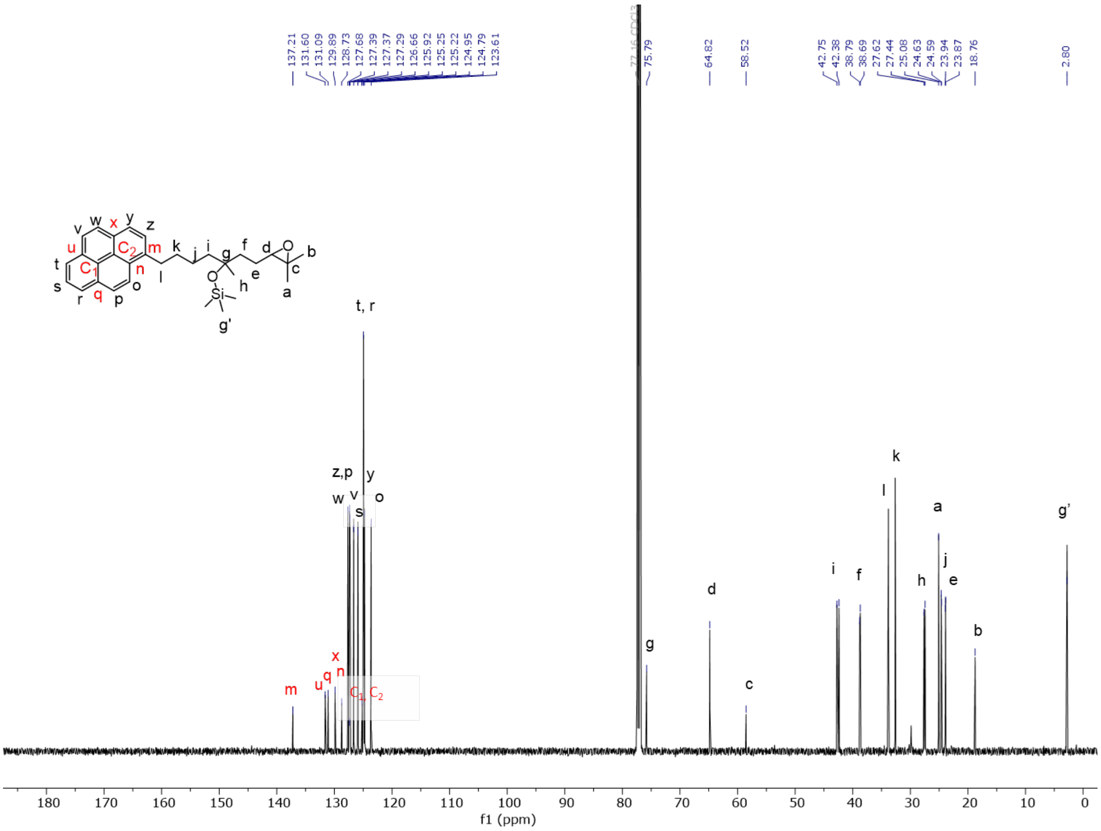

Fig. S57 126 MHz ^{13}C NMR spectrum of **33** in $CDCl_3$.

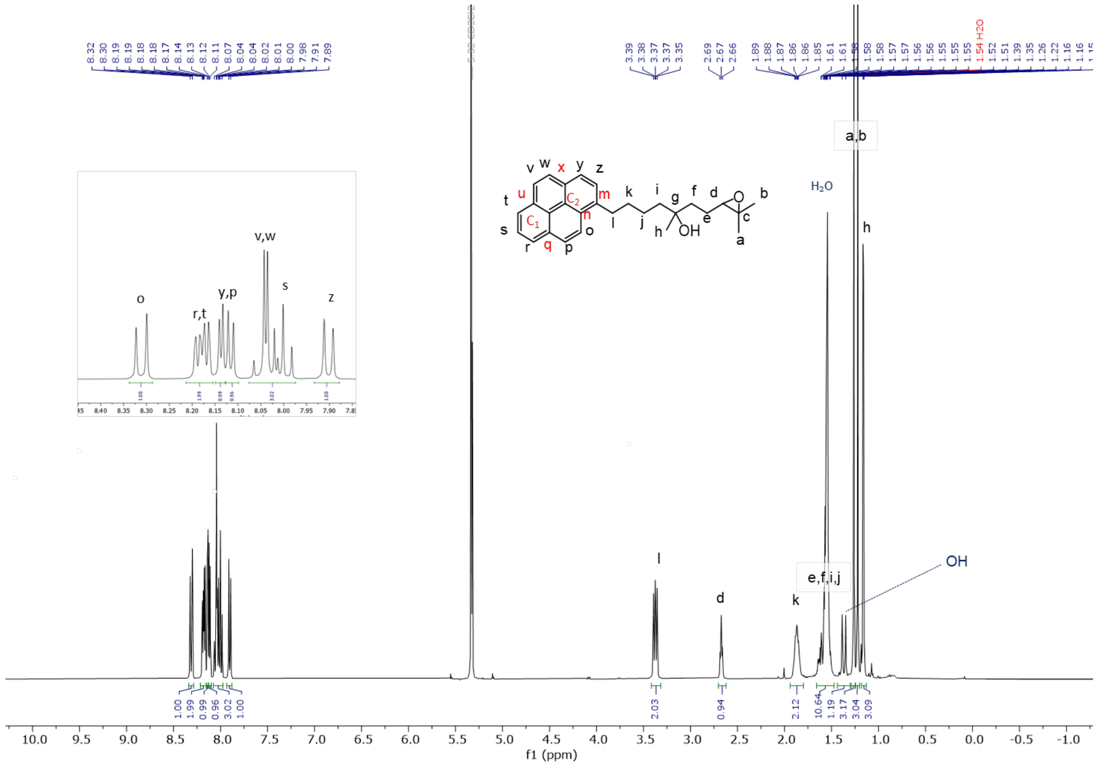

Fig. S58 500 MHz ^{1}H NMR spectrum of **5** in CD_2Cl_2.

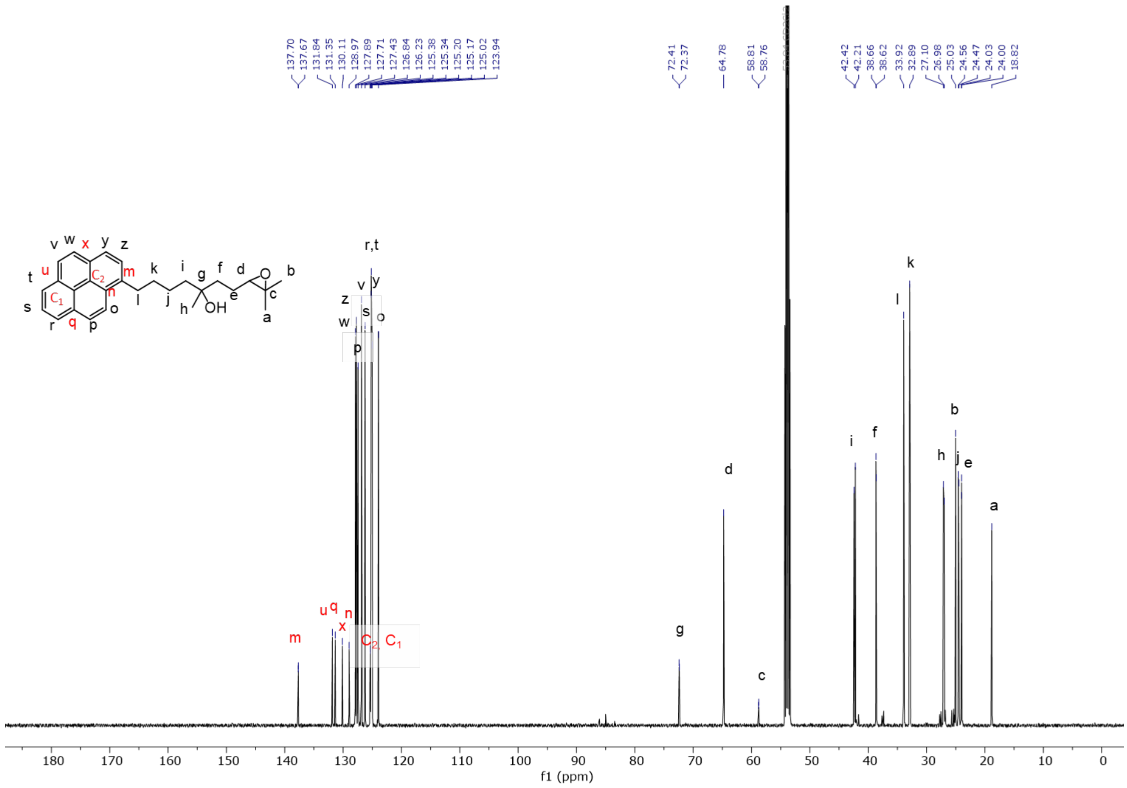

Fig. S59 126 MHz ^{13}C NMR spectrum of **5** in CD_2Cl_2

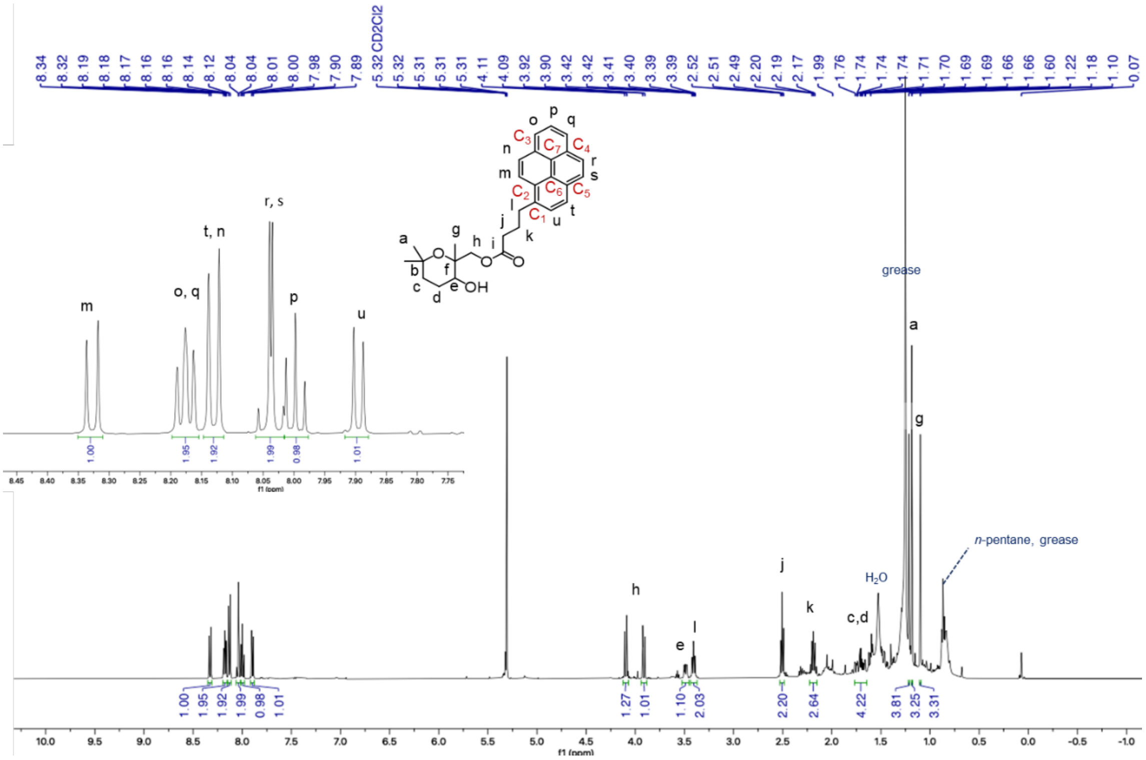

Fig. S60 500 MHz ^1H NMR spectrum of **2a** in CD_2Cl_2.

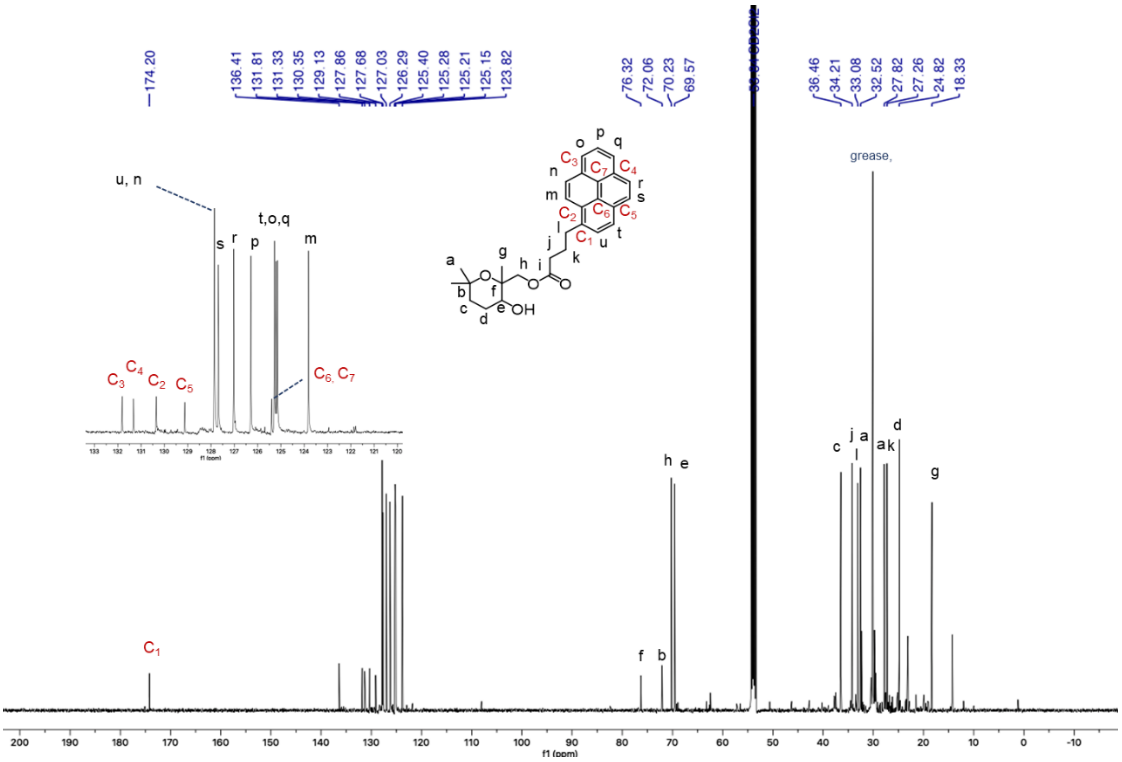

Fig. S61 126 MHz ^{13}C NMR spectrum of **2a** in CD_2Cl_2.

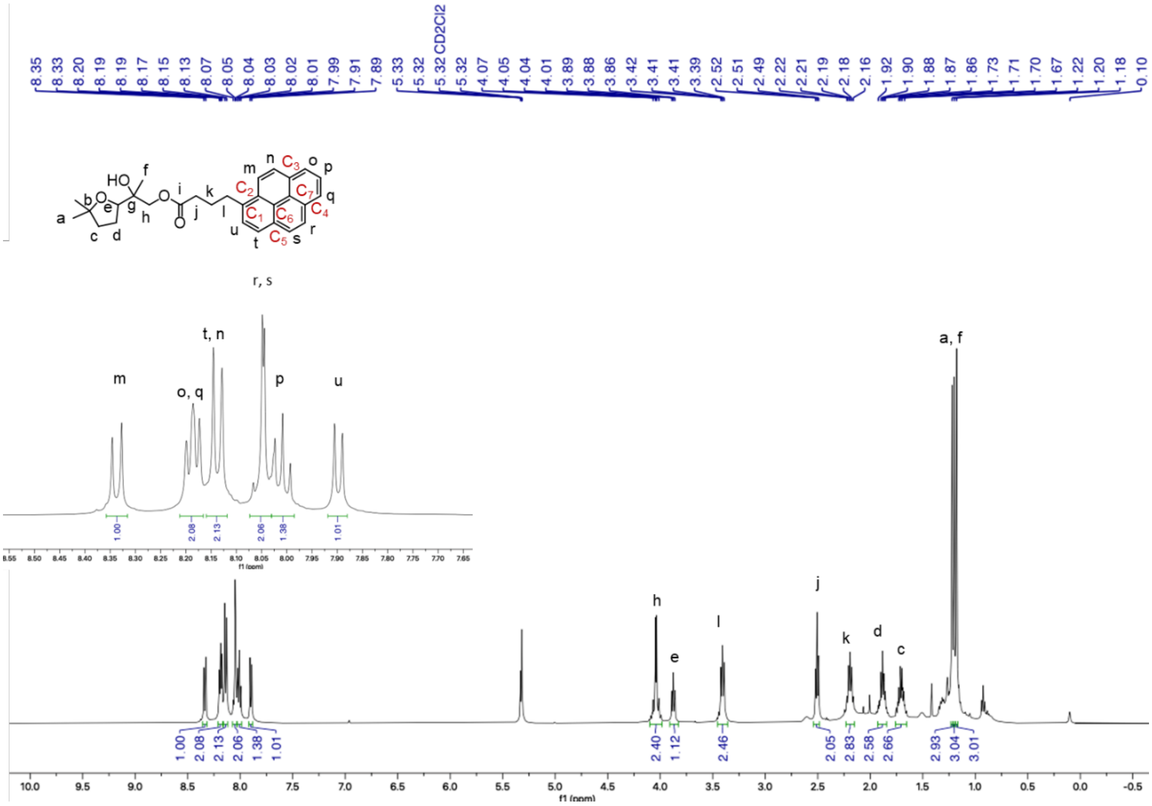

Fig. S62 500 MHz ^{1}H NMR spectrum of **2b** in CD_2Cl_2.

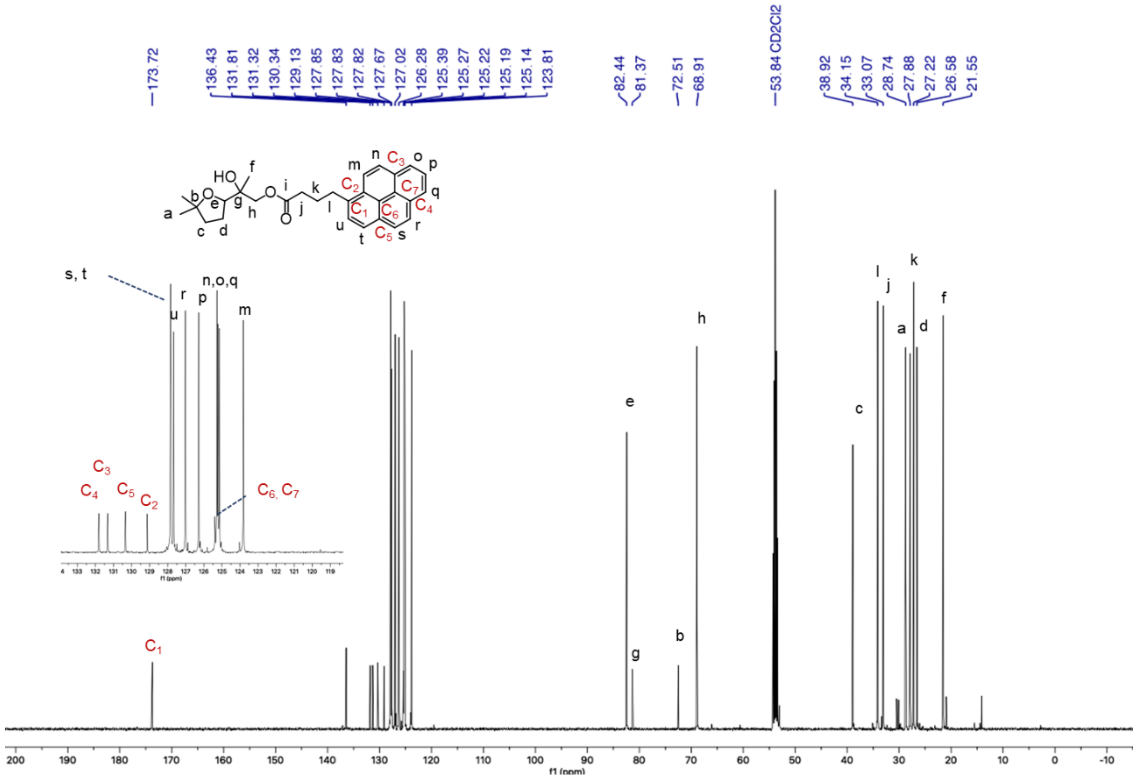

Fig. S63 126 MHz ^{13}C NMR spectrum of **2b** in CD_2Cl_2.

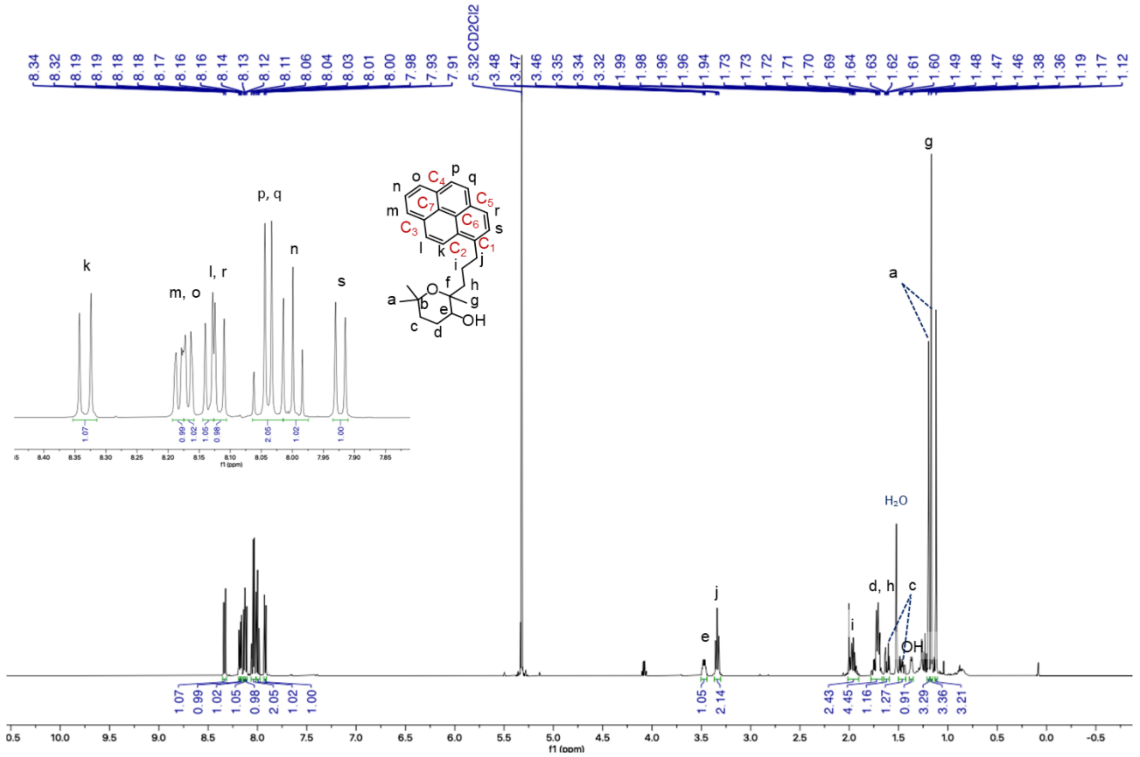

Fig. S64 500 MHz ^{1}H NMR spectrum of **3a** in CD_2Cl_2.

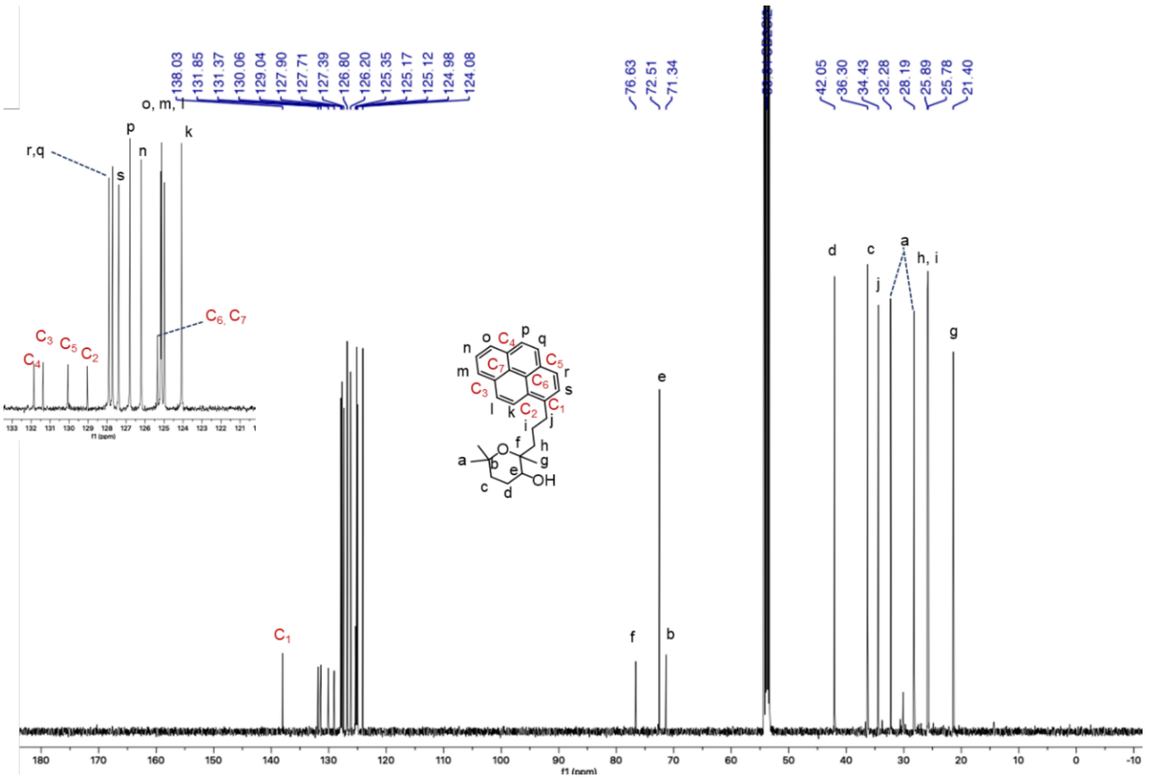

Fig. S65 126 MHz ^{13}C NMR spectrum of **3a** in CD_2Cl_2.

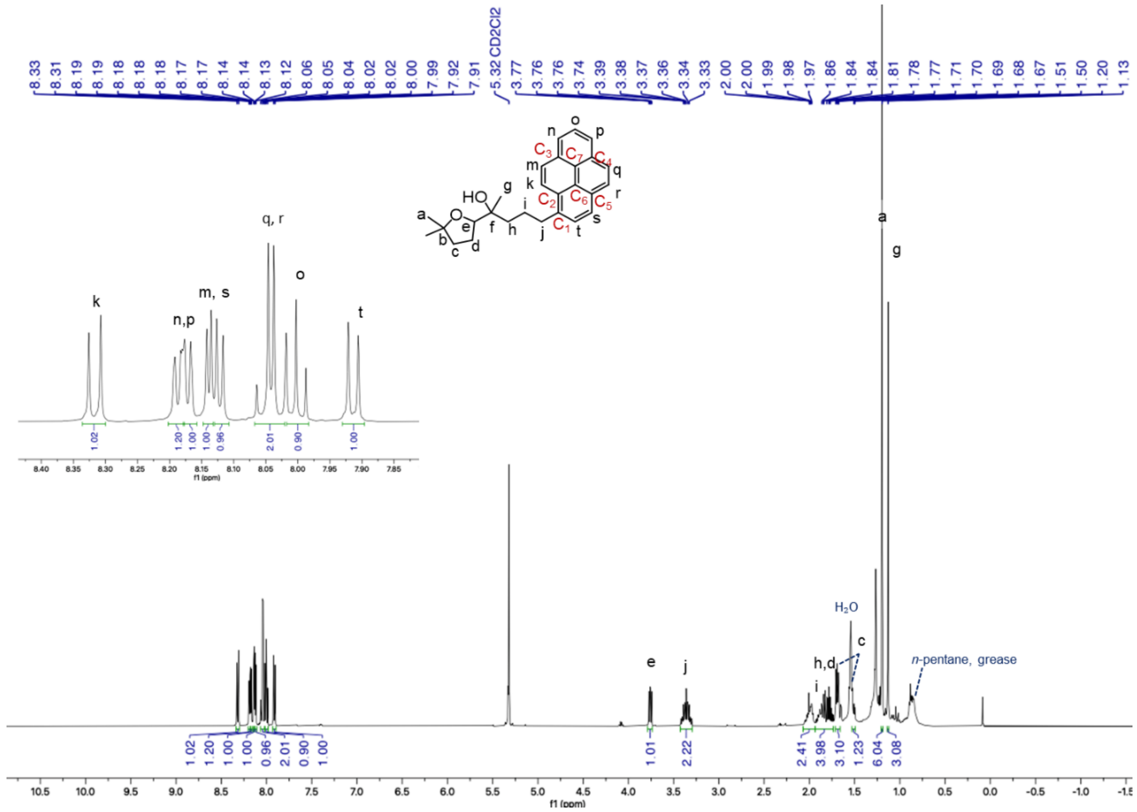

Fig. S66 500 MHz ^{1}H NMR spectrum of **3b** in CD_2Cl_2.

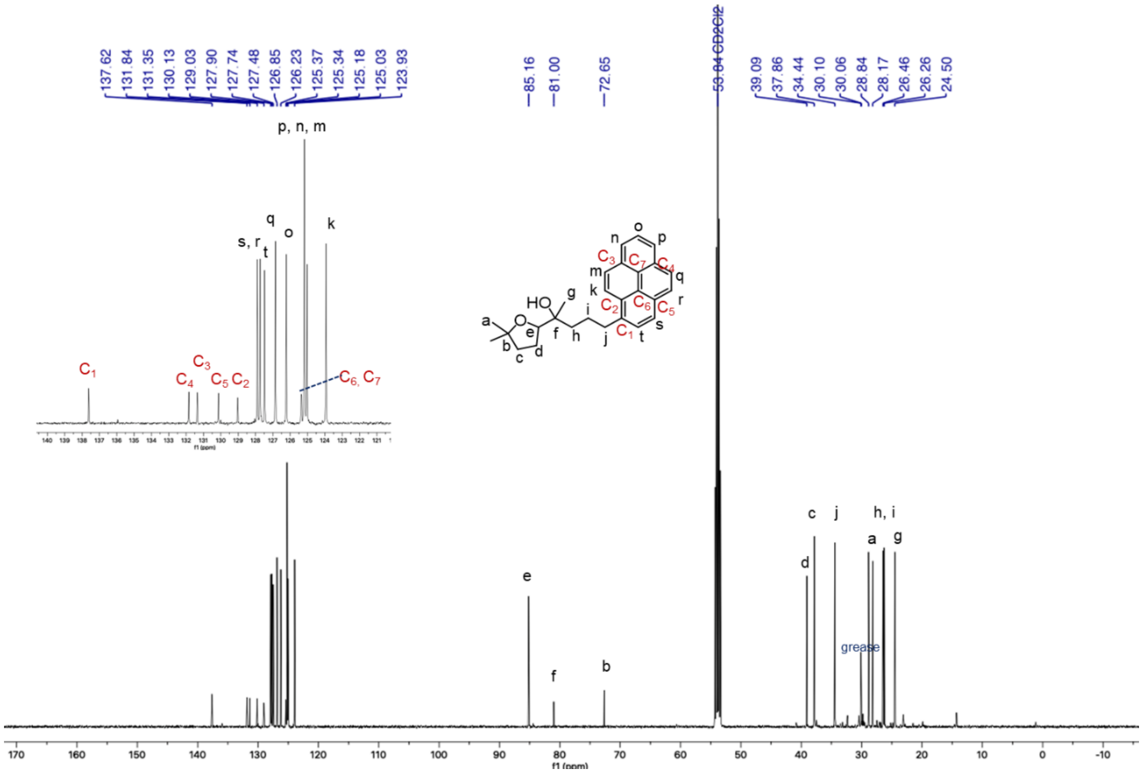

Fig. S67 126 MHz ^{13}C NMR spectrum of **3b** in CD_2Cl_2.

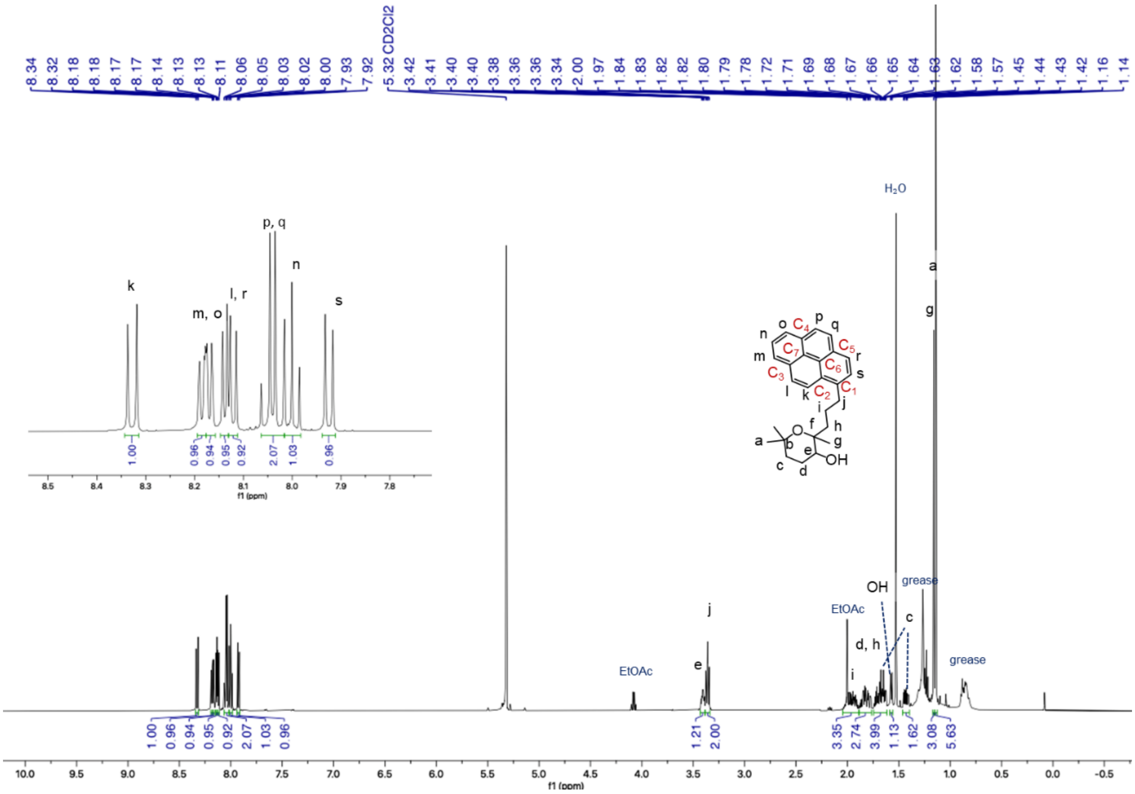

Fig. S68 500 MHz ^1H NMR spectrum of **4a** in CD_2Cl_2.

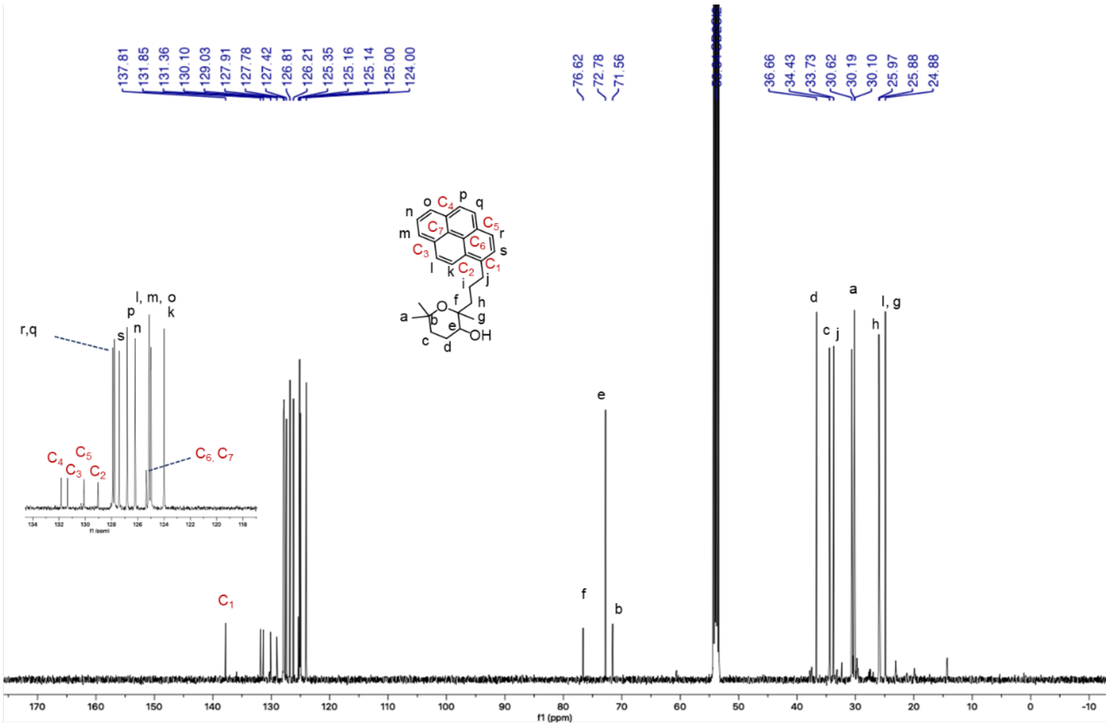

Fig. S69 126 MHz ^{13}C NMR spectrum of **4a** in CD_2Cl_2.

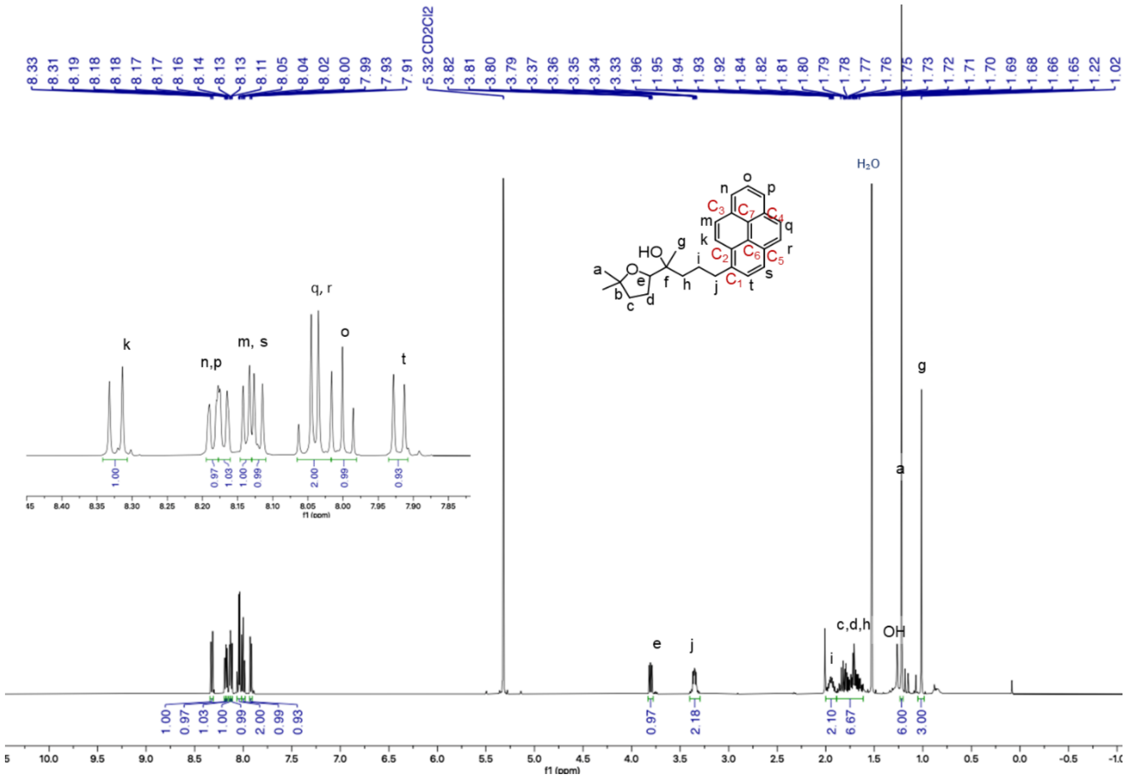

Fig. S70 500 MHz ^1H NMR spectrum of **4b** in CD_2Cl_2.

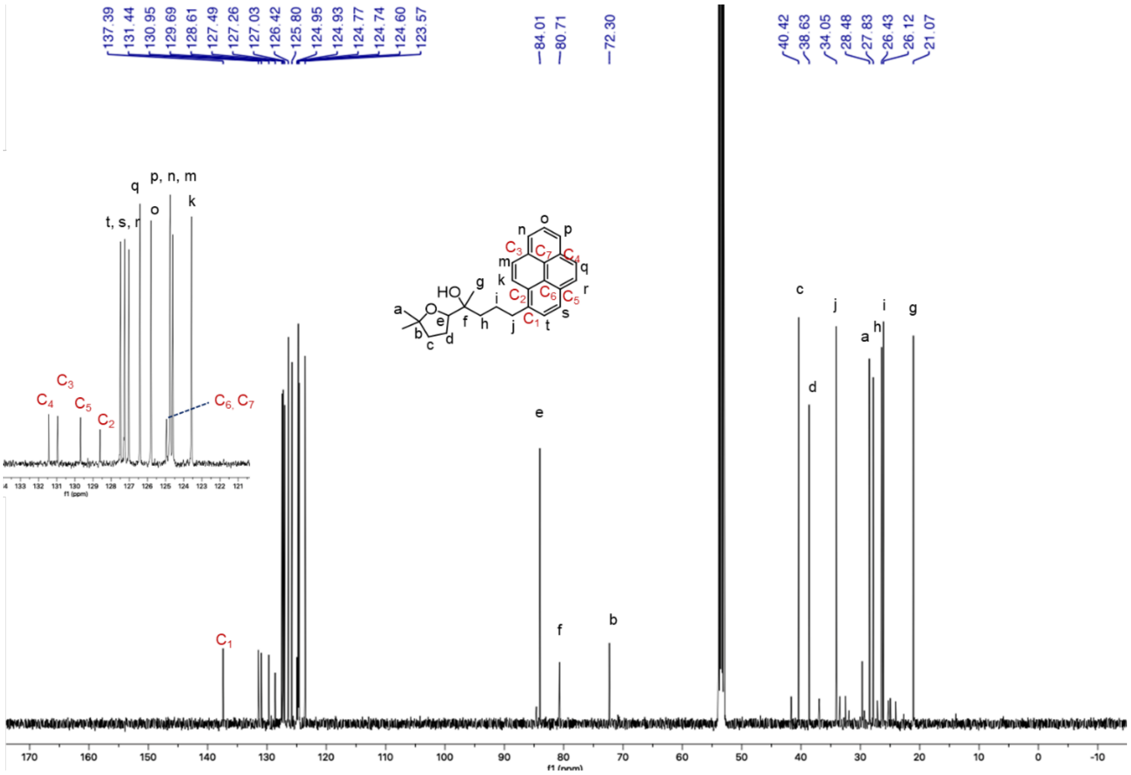

Fig. S71 126 MHz ^{13}C NMR spectrum of **4b** in CD_2Cl_2.

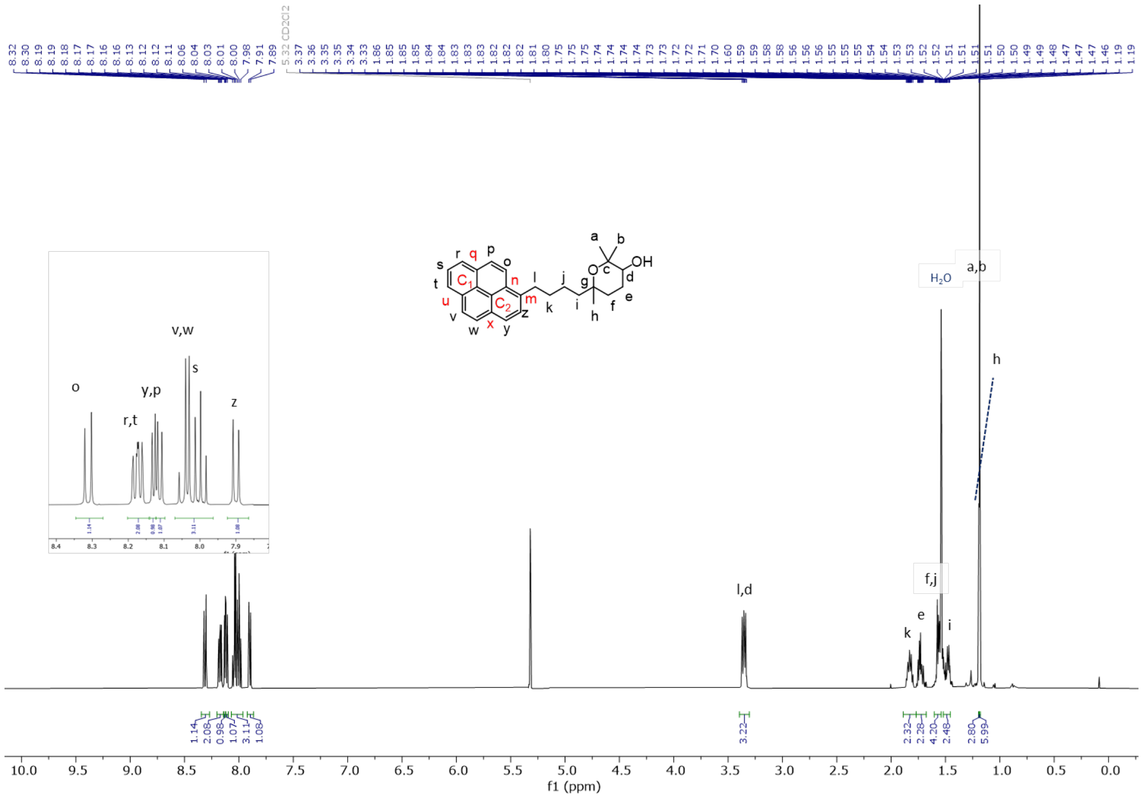

Fig. S72 500 MHz ^{1}H NMR spectrum of **5a1** in CD_2Cl_2.

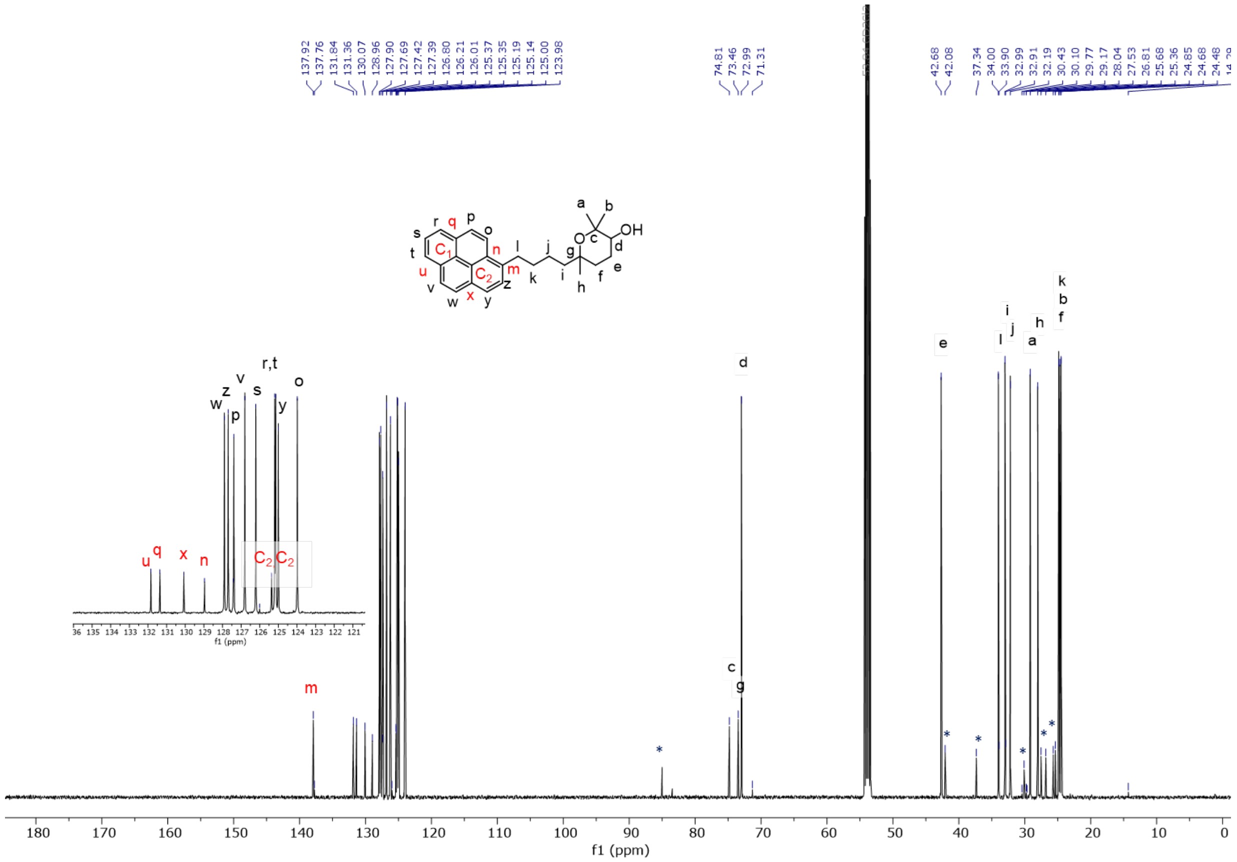

Fig. S73 126 MHz ^{13}C NMR spectrum of **5a1** in CD_2Cl_2, * **5b2** residual peaks.

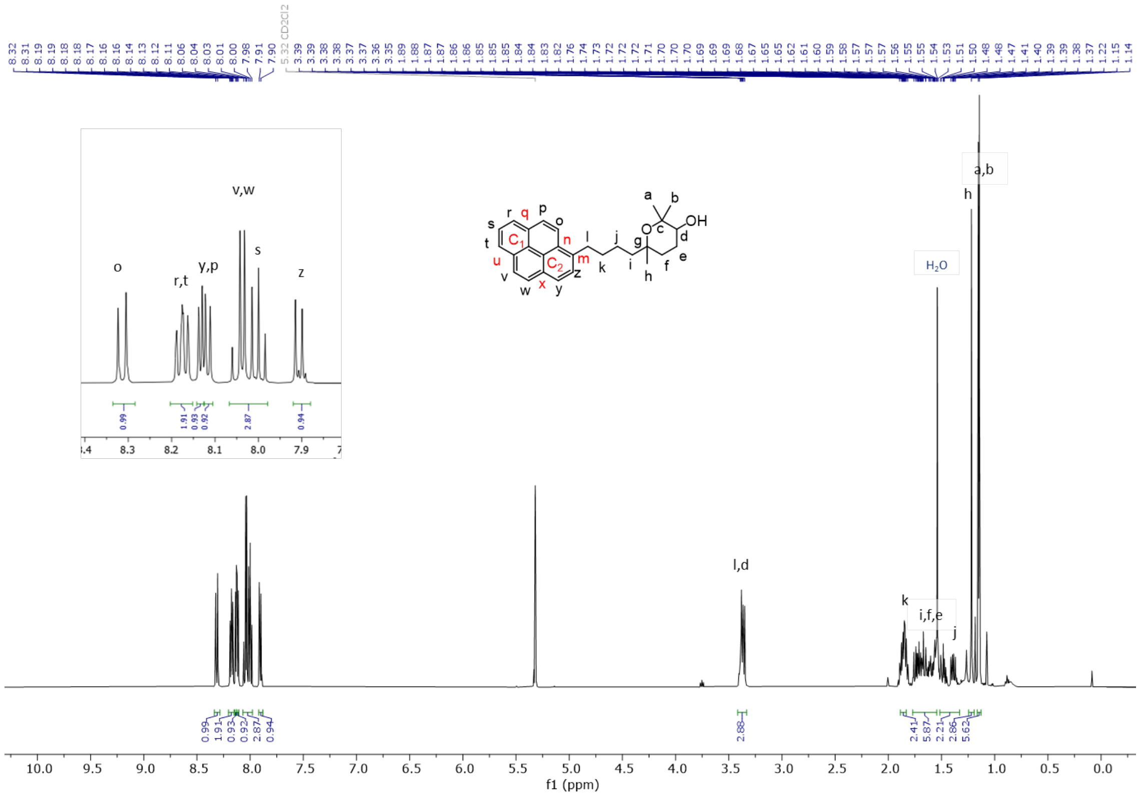

Fig. S74 500 MHz ^{1}H NMR spectrum of **5a2** in CD_2Cl_2.

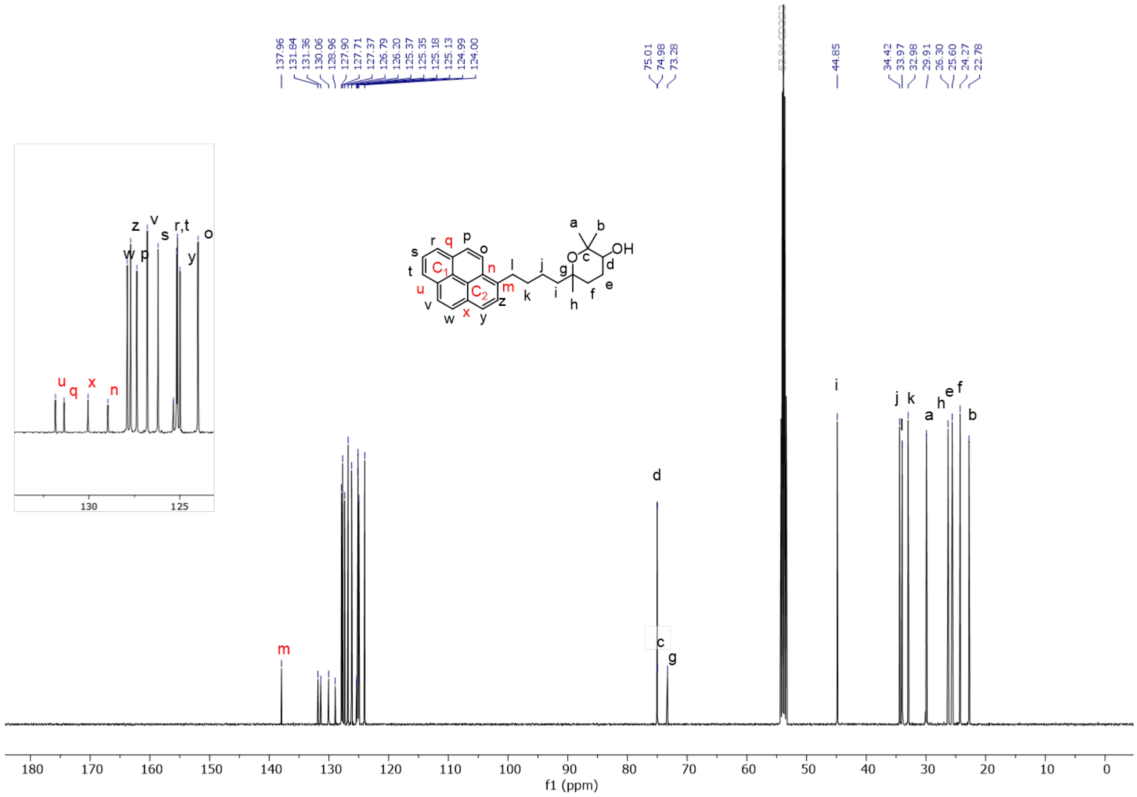

Fig. S75 126 MHz ^{13}C NMR spectrum of **5a2** in CD_2Cl_2.

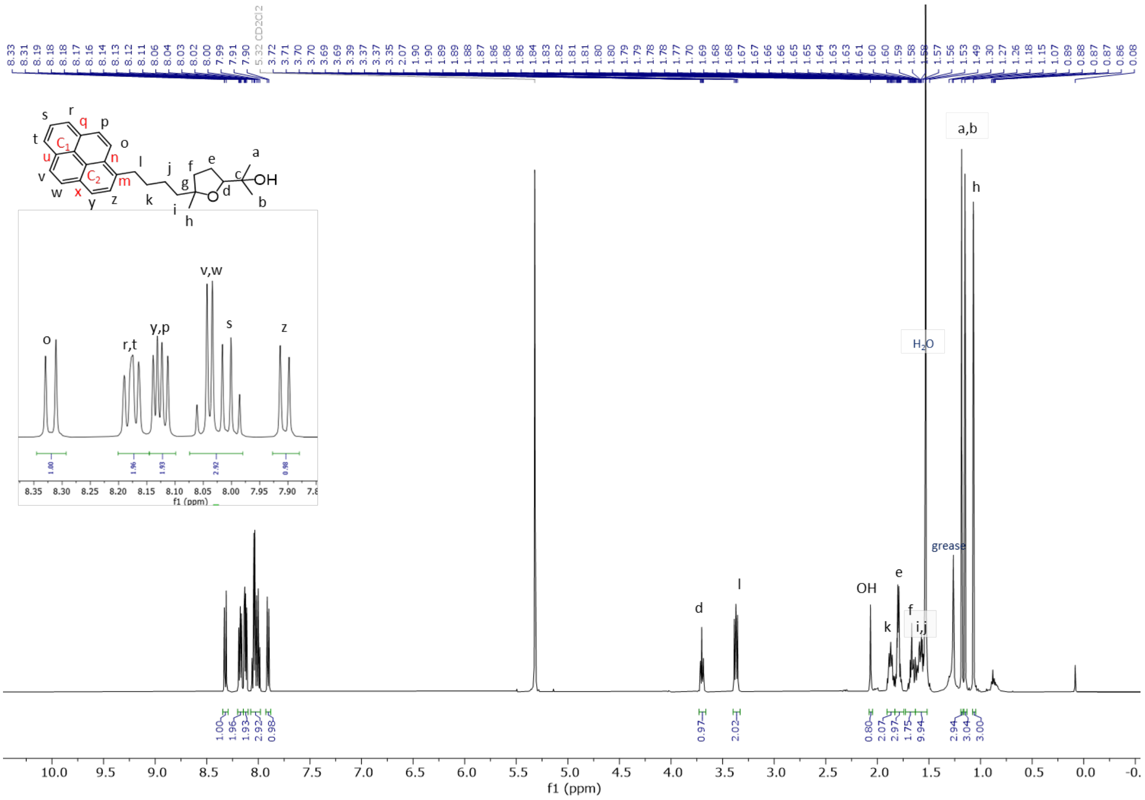

Fig. S76 500 MHz ^{1}H NMR spectrum of **5b1** in CD_2Cl_2.

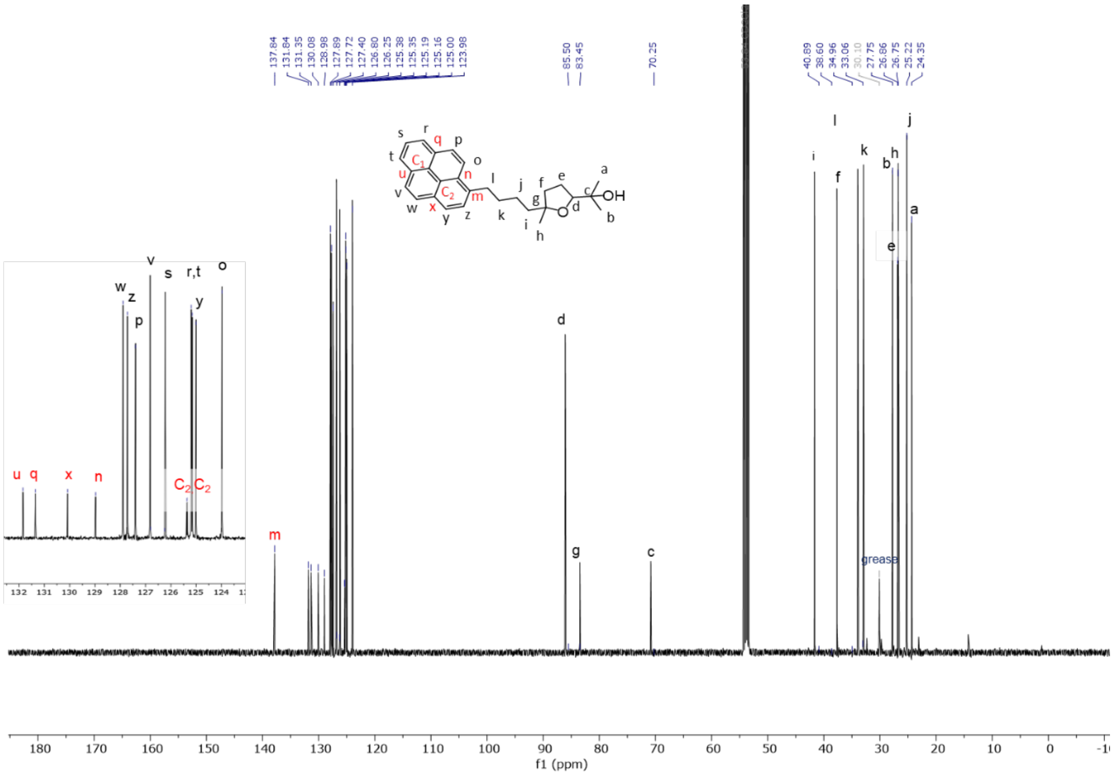

Fig. S77 126 MHz ^{13}C NMR spectrum of **5b1** in CD_2Cl_2.

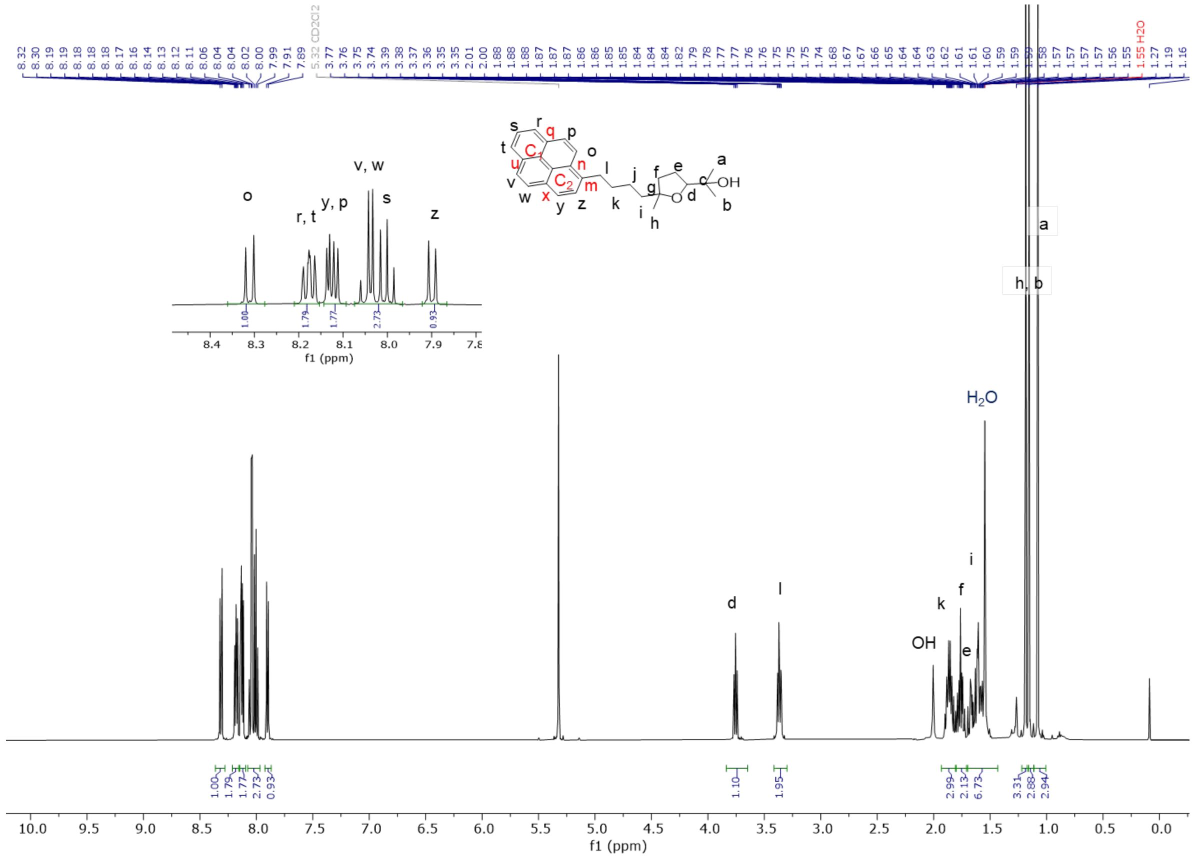

Fig. S78 500 MHz ^{1}H NMR spectrum of **5b2** in CD_2Cl_2.

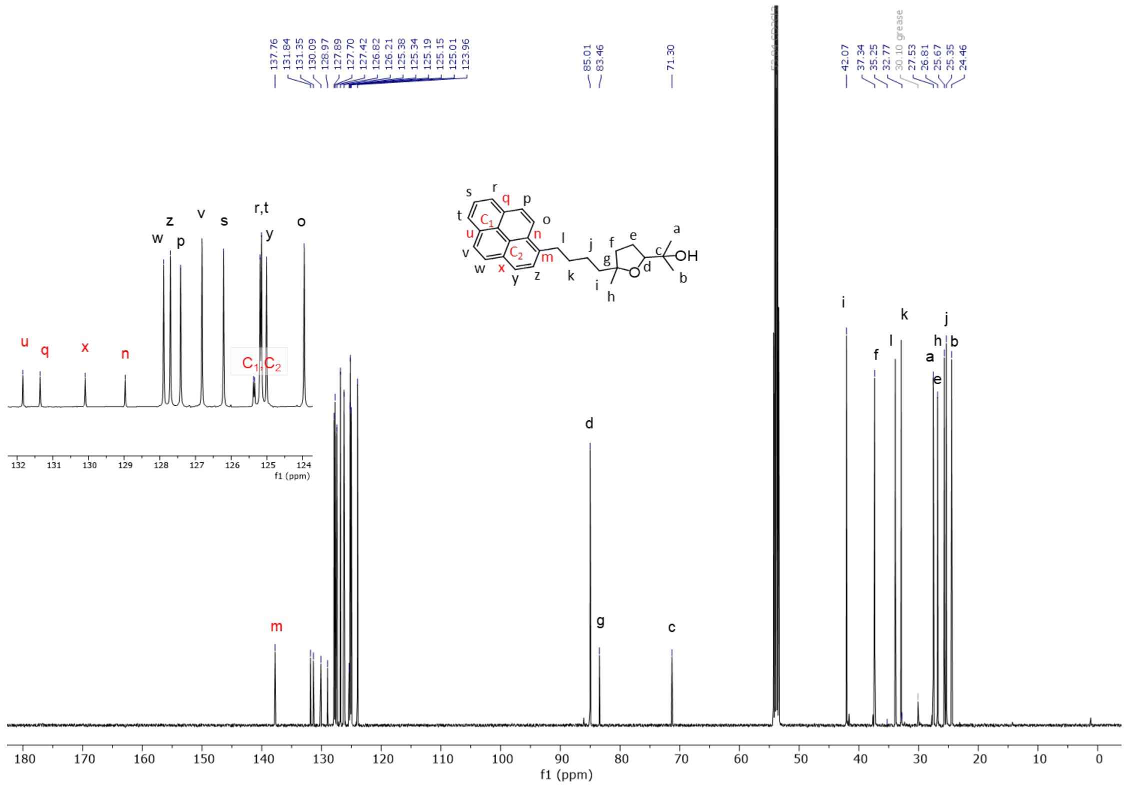

Fig. S79 126 MHz ^{13}C NMR spectrum of **5b2** in CD_2Cl_2.
